# The advantage of periodic over constant signalling in microRNA-mediated regulation

**DOI:** 10.1093/nar/gkaf867

**Published:** 2025-09-09

**Authors:** Elsi Ferro, Candela L Szischik, Alejandra C Ventura, Carla Bosia

**Affiliations:** Department of Applied Science and Technology, Politecnico di Torino, Corso Duca degli Abruzzi 24, 10129 Torino, Italy; IIGM Foundation—Italian Institute for Genomic Medicine, c/o IRCCS, SP 142 km 3,95, Candiolo, 10060 Torino, Italy; Universidad de Buenos Aires, Facultad de Ciencias Exactas y Naturales, Departamento de Física, Ciudad Universitaria, 1428 Buenos Aires, Argentina; Instituto de Fisiología, Biología Molecular y Neurociencias (IFIBYNE UBA-CONICET), Consejo Nacional de Investigaciones Científicas y Técnicas Argentina, Universidad de Buenos Aires, 1428 Buenos Aires, Argentina; Universidad de Buenos Aires, Facultad de Ciencias Exactas y Naturales, Departamento de Física, Ciudad Universitaria, 1428 Buenos Aires, Argentina; Instituto de Fisiología, Biología Molecular y Neurociencias (IFIBYNE UBA-CONICET), Consejo Nacional de Investigaciones Científicas y Técnicas Argentina, Universidad de Buenos Aires, 1428 Buenos Aires, Argentina; Department of Applied Science and Technology, Politecnico di Torino, Corso Duca degli Abruzzi 24, 10129 Torino, Italy; IIGM Foundation—Italian Institute for Genomic Medicine, c/o IRCCS, SP 142 km 3,95, Candiolo, 10060 Torino, Italy

## Abstract

Cells may exploit oscillatory gene expression to encode biological information. Temporal features of oscillations, such as pulse frequency and amplitude, are determinant for the outcome of signalling pathways. However, little effort has been devoted to unveiling the role of pulsatility in the context of post-transcriptional gene regulation, where microRNAs act by binding to RNAs and regulate their expression. Here, we study the effects of periodic against constant microRNA synthesis within minimal microRNA–target networks. We find that there is a repressive advantage of pulsatile over constant microRNA synthesis, and that the extent of repression depends on the frequency of pulses, thus uncovering frequency preference behaviours. We show that the preference for specific input frequencies is determined by relative microRNA and target kinetic rates and can lead to exclusive frequency-dependent repression on distinct RNA species, thereby highlighting a potential mechanism of selective dynamical target regulation. Moreover, we show that frequencies observed in periodically expressed microRNAs, such as those involved in circadian rhythms and development, can be selectively favored. Our findings might have implications for experimental studies aimed at understanding how periodic patterns drive biological responses through microRNA-mediated signalling and provide suggestions for validation in synthetic networks.

## Graphical Abstract

**Graphical Abstract:**
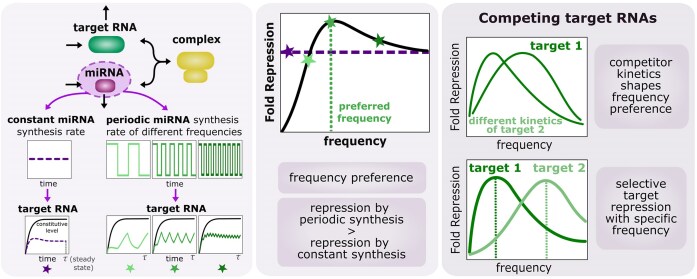

## Introduction

A number of studies have highlighted that living cells are inherently dynamic: the concentrations and activities of many molecules previously assumed to stochastically fluctuate around fixed mean values have been shown to oscillate periodically. Live-cell time-lapse microscopy and fluorescent reporter genes have allowed to track the dynamic temporal behaviour of proteins, thereby uncovering a picture where many regulators undergo periodic pulses of activation and deactivation [1, 2]. Such pulses occur through temporal changes in their concentration or localization on timescales that can span from minutes to hours. How these oscillations are generated has been addressed by single-cell experiments, revealing that genetic circuits actively generate pulses of expression of key regulators and modulate pulse features such as frequencies and amplitudes [1]. It has been shown that these features can determine the behaviour of signalling pathways by driving crucial decision-making processes such as DNA repair, cell death, and differentiation [1]. A prime example of how biological systems exploit pulse frequencies for regulatory purposes is given by [3], where the authors showed that the gonadotropin-releasing hormone GnRH is secreted in pulses whose frequency allows for bell-shaped frequency-response curves of gonadotropin secretion and gene expression characteristic of frequency decoders.

In parallel, theoretical studies have mathematically investigated the implications of pulsatility in signalling circuits and motivated its emergence in terms of biological functionality [3–5]. Furthermore, they provided quantitative tools to predict biological outcomes based on temporal signal features. For instance, mathematical modelling predicted that simple circuits that involve binding and unbinding of two molecular species, such as ligand–receptor interaction, can behave as band-pass filters by selectively responding to certain frequencies of input signals [6–8]. Another example is provided by the work by Rahi and coworkers [9], which shows how oscillatory stimuli can be used to distinguish different circuit behaviours.

Nonetheless, periodic expression of regulatory molecules has also been observed in the epigenetic context, as some post-transcriptional regulatory circuits were found to function in pulses: the interaction between microRNAs (miRNAs) and their target genes was evidenced to shape gene expression oscillations in a number of studies [10–14]. MiRNAs are evolutionarily conserved non-coding RNAs with a length of ∼22 nucleotides that act as post-transcriptional regulators of gene expression. They play fundamental roles in tumorigenesis, viral infection, and neurological diseases [15–17], as well as in key decision-making processes concerning development and differentiation [18, 19]. Their action is achieved by Watson–Crick base pairing with target RNAs, which can be either coding or non-coding. If the target is coding, the regulation can result in either prevented translation or enhanced degradation [20] depending on the degree of miRNA–RNA complementarity [21, 22]. Under certain conditions, the outcome of miRNA–mRNA interaction can be the degradation of the miRNA, unlike what occurs in the canonical pathway [23]. If the target is a non-coding RNA—such as circular or long non-coding RNAs—there is little or no effect on their stability. These non-coding RNAs may function as miRNA sponges, thus affecting their potential to regulate other targets [24, 25].

miRNA-mediated regulation is combinatorial, meaning that a single miRNA may regulate multiple targets, and a single target is typically regulated by different miRNAs [21, 26]. This combinatorial regulation has been shown to generate crosstalk between different RNAs competing for interacting with the same miRNA species, a scenario also known as competing endogenous RNA (ceRNA) effect [27–30].

miRNAs are involved in periodic signalling circuits in diverse organisms and biological contexts, ranging from development and differentiation to circadian rhythms [31–34]. Some miRNAs have been shown to exhibit ultradian oscillatory expression patterns during the cell cycle, as reported by [35]. Numerous miRNAs display circadian rhythmicity [36–42]. Moreover, some miRNAs undergo periodic transcription: miR-9 is rhythmically transcribed through a feedback loop with Hes1 [11], lin-42 negatively regulates the rhythmic biogenesis of key miRNAs such as let-7 and lin-4 [43], and in mouse hepatic miRNAs oscillate in coordination with feeding cycles [44]. In *Caenorhabditiselegans*, developmental timing is linked to rhythmic accumulation of mature miRNAs, such as lin-4 and let-7, driven by both periodic transcription and regulated degradation [45]. Kim and co-workers showed that the lin-4 miRNA can oscillate with a precise frequency to mask regulatory pulses of a transcription factor, thus maintaining a temporal gradient of its target functional for *C. elegans* development [10].

However, the functional role of pulsatile post-transcriptional regulation is poorly understood—with theoretical predictions focusing mainly on small perturbations at the steady state [46]—and miRNAs have been largely overlooked by mathematical studies on periodic signalling.

Here, we theoretically address pulsatile miRNA expression as opposed to constant expression, focusing on the functional role of the oscillation frequency. Using a minimal model where miRNA and RNA species undergo simple binding and unbinding, we simulate pulsatile miRNA synthesis with variable frequency. We find that pulsatile miRNA synthesis can achieve a stronger average target repression than a constant synthesis with identical miRNA-to-target dose. Furthermore, we show that the extent of repression depends on the frequency of pulses and thus uncover potential frequency preference behaviours. We then show that the repressive advantage of pulsatile over constant synthesis and the system’s ability to be selective for specific frequency bands is determined by the relative miRNA/target kinetic features, suggesting that periodic miRNA signalling might provide a way to select targets with specific kinetics.

Since the ability to selectively regulate different targets can be particularly meaningful in the context of post-transcriptional repression, where many different RNA species are regulated by the same miRNA, we build a modified minimal model where an miRNA targets distinct RNA species. We show that the kinetic features of a competitor RNA can affect and shift a target’s frequency-dependent repression. Eventually, we show that each competing RNA can be selectively repressed using distinct miRNA synthesis frequencies. Our findings thus highlight periodic miRNA expression as a versatile mechanism of differential and dynamical target regulation.

Overall, our findings suggest that periodicity might confer a functional advantage to post-transcriptional regulation and provide indications on experimental parameter tuning for validation in live cells.

## Materials and methods

### Modelling miRNA–target RNA interaction: model M1

**Figure 1.**
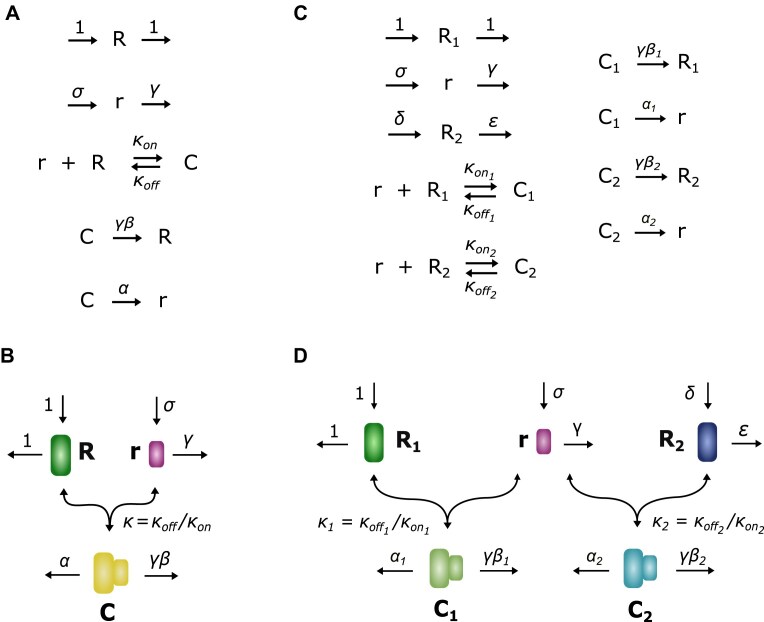
miRNA–RNA interaction models. (**A**) Biochemical reactions describing miRNA–RNA interaction in model M1. (**B**) Schematic of miRNA–RNA interaction model M1. (**C**) Biochemical reactions describing miRNA interaction with multiple target RNAs in model M2. (**D**) Schematic of miRNA–RNA interaction model M2.

The miRNA–RNA model M1 is based on mass-action kinetics and considers a single target RNA species [47]. Both the RNA and the miRNA are synthesized through transcription. The two molecular species can bind to form a complex, and each of them can be degraded both when unbound and when in complex. The model assumes that RNA and miRNA are degraded independently when in complex, as supported by previous data [48–50]: either the RNA is degraded and the miRNA is recycled into the system or vice versa (Fig. 1A and B).

Our model includes eight chemical reactions (see Supplementary Section S1): synthesis and degradation of each species, binding and unbinding, and degradation of each species in complex. The latter reactions imply that the species that does not undergo degradation—either the miRNA or the RNA—is recycled back into the system, and are thus often called recycling reactions. Similarly to what is done in [47], ordinary differential equations were nondimensionalized by scaling variables and parameters with the RNA degradation rate constant and its synthesis rate constant (see Supplementary Section S1). This nondimensionalization choice makes the equations only dependent on relative parameters of miRNA and RNA, and thus allows us to derive more general predictions that rely on relative kinetic properties of regulator and target rather than on the specific features of each species.

The dimensionless form of the mass-action model M1 is described by the following ODE system:


(1) \begin{eqnarray*}
\left\lbrace \begin{array}{@{}l@{\quad }l@{}}&\frac{dR}{dt} = 1 - R - \kappa _{\rm {on}} r R + \kappa _{\rm {off}} C + \gamma \beta C, \\ & \frac{dr}{dt} = \sigma -\gamma r - \kappa_{\rm {on}} r R + \kappa _{\rm {off}}C + \alpha C, \\ & \frac{dC}{dt} = \kappa _{\rm {on}} r R - \kappa _{\rm {off}} C - \alpha C - \gamma \beta C, \end{array}\right.
\end{eqnarray*}

where *R*, *r*, and *C* represent dimensionless concentrations of unbound RNA, unbound miRNA, and RNA–miRNA molecular complex, respectively. Due to the scaling choice (see Supplementary Section S1), the RNA synthesis and degradation rates are scaled to 1. The parameter *σ* represents the synthesis rate of miRNA relative to RNA; *κ*_on_ and *κ*_off_ represent, respectively, the scaled association and dissociation rates, thus yielding a dimensionless dissociation constant *K* = *κ*_off_/*κ*_on_; *γ* is the degradation rate of miRNA relative to RNA; and *α* and *β* represent, respectively, the scaled degradation rates of RNA and miRNA in complex, which describe how fast each species is degraded in complex relative to its unbound form, and thus how fast the other species is instead recycled. As a result of nondimensionalization, in this model one time unit corresponds to ∼1/*ln*(2) ≈ 1.44 RNA half-lives (see Supplementary Section S1).

This model has a single positive steady state (see an analytical proof in Supplementary Section S1.1).

### Modelling multiple miRNA targets: model M2

Model M2 is based on assumptions identical to model M1, but considers two target RNA species. Similar to the first model, both the miRNA and the RNAs are synthesized via transcription and each RNA species can form a complex with the miRNA. Each RNA species has the capability to interact with the miRNA to form a complex. All species, whether unbound or in complex form, can undergo degradation. Degradation occurs independently for the miRNA and RNA, resembling the degradation dynamics outlined by model M1 (Fig. 1C and D). Equations were nondimensionalized by scaling variables and parameters as in model M1, using the first RNA’s degradation rate constant and its synthesis rate constant (see Supplementary Section S2). Similar to model M1, nondimensionalization allows us to deal with relative kinetic features of the involved molecular species rather than absolute ones. This allows our predictions to span more general kinetic conditions than in a dimensional framework. The dimensionless form of the mass-action model M2 is described by the following ODE system:


(2) \begin{eqnarray*}
\left\lbrace \begin{array}{@{}l@{\quad }l@{}}&\frac{dR_1}{dt} = 1 - \kappa _{{\rm on}_1} r R_1 + \kappa _{{\rm off}_1} C_1 - R_1 + \gamma \beta _1 C_1, \\ &\frac{dR_2}{dt} = \delta - \kappa _{{\rm on}_2} r R_2 + \kappa _{{\rm off}_2} C_2 - \epsilon R_2 + \gamma \beta _2 C_2, \\ &\frac{dr}{dt} = \sigma -\gamma r -\kappa _{{\rm on}_1} r R_1 + \kappa _{{\rm off}_1} C_1 + \alpha _1 C_1 \\ & \qquad -\, \kappa _{{\rm on}_2} r R_2 + \kappa _{{\rm off}_2} C_2 + \alpha _2 C_2, \\ &\frac{dC_1}{dt} = \kappa _{{\rm {\rm on}}_1} r R_1 - \kappa _{{\rm off}_1} C_1 - \gamma \beta _1 C_1 - \alpha _1 C_1, \\ &\frac{dC_2}{dt} = \kappa _{{\rm on}_2} r R_2 - \kappa _{{\rm off}_2} C_2 - \gamma \beta _2 C_2 - \alpha _2 C_2, \end{array}\right.
\end{eqnarray*}

where *R*_1_ and *R*_2_ represent dimensionless concentrations of the two unbound RNA species, *r* is the dimensionless concentration of unbound miRNA, and *C*_1_ and *C*_2_ represent the two molecular complexes formed with the miRNA by the two distinct RNA species. Here, *σ* represents the synthesis rate constant of miRNA relative to that of the first RNA target. $\kappa _{{\rm on}_i}$ and $\kappa _{{\rm off}_i}$ represent miRNA binding and unbinding rates of the *i*th RNA species. *α*_*i*_ describes the degradation rate of the *i*th RNA in complex relative to that of its unbound form. *β*_*i*_ is the degradation rate of miRNA in the *i*th complex relative to its unbound form. *γ* is the degradation rate of unbound miRNA relative to that of the first RNA species. *δ* and *ε* describe, respectively, the synthesis and the degradation rate of the second RNA species with respect to the first one.

### Modelling periodic miRNA synthesis

Periodic miRNA synthesis is fed to the nondimensional ODE systems by modelling the regulator’s transcription rate *σ* as a square wave (see Supplementary Section S3 for nondimensionalization details):


(3) \begin{eqnarray*}
\sigma _{\rm {pulse}} = \left\lbrace \begin{array}{@{}l@{\quad }l@{}}\sigma & \text{if } nT < \; t \le (n+d)T, \quad n = 0, 1, 2, \ldots, \\ 0 & \text{if } (n+d)T < \; t \le (n+1)T, \quad n = 0, 1, 2, \ldots, \end{array}\right.
\end{eqnarray*}

where *σ* represents the square wave’s amplitude, *d* represents its duty cycle, and *T* represents its period.

Note that the scaling of time resulting from model nondimensionalization (Supplementary Section S1) implies that the square wave’s frequency is also scaled: a nondimensional frequency of 1 corresponds to pulses occurring every ≈1.44 half-lives of the RNA *R* in model M1 (or *R*_1_ in model M2).

### Relative miRNA–RNA dose conservation

Since we are interested in effects driven solely by the frequency of periodic miRNA synthesis, we need to ensure that the dose of regulator fed to the system does not change as we vary the frequency of pulses. To quantify this dose, we computed molecule amounts synthesized in an input pulse period (see Supplementary Section S4). In this way, we determined that the amount of miRNA produced relative to target *R* in model M1 (*R*_1_ in model M2) in one period is


(4) \begin{eqnarray*}
\mathrm{Relative\ miRNA -RNA1\ dose} = \sigma \cdot d,
\end{eqnarray*}

whereas the miRNA amount synthesized relative to target *R*_2_ in one period is


(5) \begin{eqnarray*}
\mathrm{Relative\ miRNA - RNA2\ dose} = \frac{\sigma }{\delta } \cdot d.
\end{eqnarray*}

These quantities are independent from the input pulse frequency. Therefore, considering an integer number of periodic miRNA synthesis pulses, it is sufficient to keep constant the nondimensional miRNA synthesis rate *σ*, the duty cycle *d*, and in model M2 also the relative synthesis rate of the two targets *δ*, to ensure that the amount of miRNA synthesized with respect to its targets is maintained constant across different frequencies (see Supplementary Section S4 for computation details).

Despite alternative strategies of dose conservation rely on varying the duty cycle *d* and appropriately compensating the amplitude σ, we keep both values fixed as we vary frequency. Indeed, we adopt a fixed duty cycle *d* = 0.5 relying on the observation that endogenous gene expression pulses commonly exhibit comparable ON and OFF pulse durations even beyond the context of circadian rhythms [51, 52]. Conversely, as the magnitude of gene expression pulses characterizes the specific biochemical process, we still explore the role of amplitude σ by parameter sensitivity analysis in the “Results” section.

To compare periodic and constant syntheses, we also model a constant rate of miRNA synthesis that satisfies dose conservation. By imposing that the relative miRNA–RNA doses produced by constant and periodic synthesis rates are identical (see Supplementary Section S4), we determined that a constant miRNA synthesis rate


(6) \begin{eqnarray*}
\sigma _{\rm {const}} = \sigma \cdot d
\end{eqnarray*}

ensures the same relative miRNA–RNA amount produced in an integer number of periodic pulses, in both models M1 and M2.

### Model analysis: fold repression

To analyze model responses, we use target gene fold repression as the primary output. Fold repression is a commonly used measurement in the experimental quantification of miRNA-mediated regulation [53], defined as the fold change between the average constitutive level of target gene expression and its average level resulting from miRNA action:


(7) \begin{eqnarray*}
{\rm Fold \ repression \, = \, \frac{\langle constitutive \ RNA \ level \rangle }{\langle repressed \ RNA \ level \rangle }}.
\end{eqnarray*}

To compare the fold repression induced by periodic and constant miRNA syntheses, we compute the average fold repression achieved by both kinds of stimuli over the same timespan. Such timespan ranges from the start of the stimulus to a generic timepoint *τ*, i.e. [0, *τ*]. Thus, for a given target *R*_*i*_ (be it the only target *R* of model M1 or one of the two targets *R*_1_ and *R*_2_ in model M2) we indicate ${\rm FR}_{\rm {const}}^{i}$ and ${\rm FR}_{\rm {pulse}}^{i}$ as its average fold repression values induced, respectively, by constant and periodic miRNA synthesis rates, i.e. *σ*_const_ and *σ*_pulse_, in the time interval [0, *τ*]:


(8) \begin{eqnarray*}
{\rm FR}_{\rm {const}}^{i} = \frac{\langle R_{\rm {basal}}^{i}(t) \rangle _{[0, \tau ]}}{\langle R_{\rm {const}}^{i}(t) \rangle _{[0, \tau ]}},
\end{eqnarray*}

(9) \begin{eqnarray*}
{\rm FR}_{pulse}^{i} = \frac{\langle R_{basal}^{i}(t) \rangle _{[0, \tau ]}}{\langle R_{pulse}^{i}(t) \rangle _{[0, \tau ]}},
\end{eqnarray*}

where $R_{\rm {basal}}^{i}(t)$ represents the temporal concentration of the *i*th target RNA obtained in the absence of miRNA (i.e. its constitutive concentration), $R_{\rm {const}}^{i}(t)$ represents its repressed concentration induced by the constant miRNA synthesis rate *σ*_const_, and $R_{\rm {pulse}}^{i}(t)$ represents its repressed concentration induced by the periodic miRNA synthesis *σ*_pulse_. While the value of ${\rm FR}_{\rm {const}}^{i}$ depends only on the system’s kinetic parameters, ${\rm FR}_{\rm {pulse}}^{i}$ will depend also on the frequency of pulses and will be thus indicated as ${\rm FR}_{\rm {pulse}}^{i}(f)$. Note that both fold repression measurements—${\rm FR}_{\rm {const}}^{i}$ and ${\rm FR}_{\rm {pulse}}^{i}$—are ratios between variable values and are thus independent from the scaling of variables used for model nondimensionalization.

To compute ${\rm FR}_{\rm {pulse}}^{i}(f)$ as a function of the frequency *f*, we numerically solve the ODE system for different frequencies of the periodic miRNA synthesis rate *σ*_pulse_. Since in our nondimensional models conservation of the relative miRNA-to-RNA dose is ensured for complete pulses, we consider time intervals [0, *τ*] containing an integer number of periods, thus posing a constraint on the minimum frequency value that can be explored for a fixed value of τ: the lowest possible frequency will be *f*_min_ = 1/*τ*, whereas we fix the maximum frequency to *f*_max_ = 10^2^/*τ*.

### Quantification of frequency-dependent responses

To compare fold repression responses generated by constant and periodic miRNA synthesis rates, we define different metric quantities.

The metric “advantage” *A* quantifies the repressive advantage of periodic over constant miRNA synthesis, comparing fold repression values achieved by the two kinds of input. For a generic target *R*_*i*_, our advantage metric reads as:


(10) \begin{eqnarray*}
A_i = \frac{\int _{f_{\rm {min}}}^{f_{\rm {max}}} [{\rm FR}_{\rm {pulse}}^{i}(f)-{\rm FR}_{\rm {const}}^{i}] \: df}{\int _{f_{\rm {min}}}^{f_{\rm {max}}} {\rm FR}_{\rm {pulse}}^{i}(f) \: df}.\
\end{eqnarray*}

**Figure 2.**
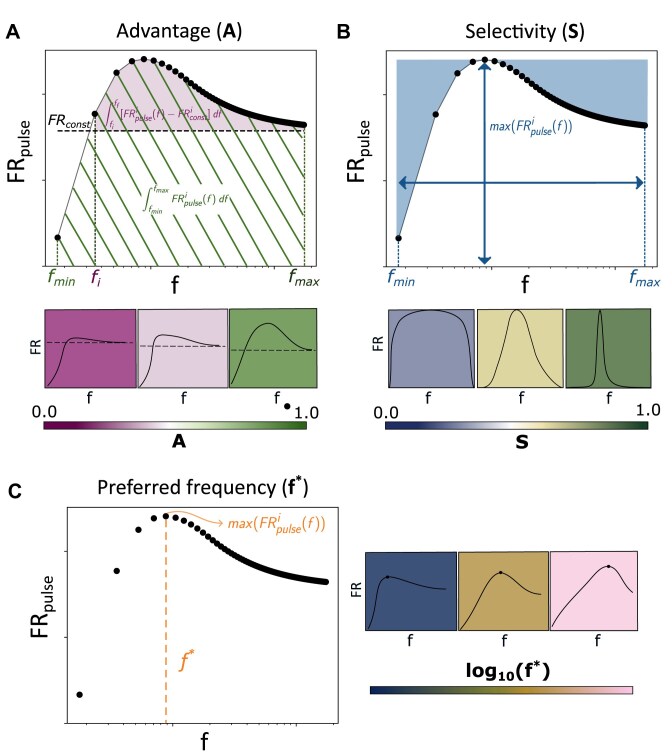
Metrics of frequency-dependent fold repression response. (**A**) Illustrative plot describing the advantage metric *A*. Dots represent fold repression FR_pulse_ as a function of frequency *f*, whereas the dashed line represents FR_const_. The striped area represents the integral of ${\rm FR}_{\rm {pulse}}^{i}$ in [*f*_min_, *f*_max_] (i.e. the denominator of *A*), whereas the shaded area represents its integral above ${\rm FR}_{\rm {const}}^{i}$ (i.e. the numerator of *A*). The three bottom plots depict three cases presenting increasing advantage *A* values. (**B**) Illustrative plot describing the selectivity metric *S*. Dots represent fold repression FR_pulse_ as a function of frequency *f*. The white area represents the integral of FR_pulse_ in [*f*_min_, *f*_max_] (i.e. the numerator of the ratio in *S*), whereas the area spanned by horizontal and vertical arrows represents the square defined by *f*_min_, *f*_max_ and the maximal value of ${\rm FR}_{\rm {pulse}}^{i}$—(i.e. the denominator of the ratio in *S*). The three bottom plots depict three cases presenting increasing selectivity *S* values. (**C**) Illustrative plot describing the preferred frequency f^*i^. Dots represent fold repression FR_pulse_ as a function of frequency *f*. The dashed line indicates f*, i.e. the frequency corresponding to the maximal value of FR_pulse_. The three right plots depict three cases presenting increasing f* values.

Note that *A*_*i*_ is bounded in the range [0, 1]. The broader the range of frequencies whose fold repression response ${\rm FR}_{\rm {pulse}}^{i}(f)$ overcomes that of the constant stimulus ${\rm FR}_{\rm {const}}^{i}$, and the greater the difference between ${\rm FR}_{\rm {pulse}}^{i}(f)$ and ${\rm FR}_{\rm {const}}^{i}$, the higher the resulting advantage *A*_*i*_ will be (see Fig. 2A).

Since the frequency range where ${\rm FR}_{\rm {pulse}}^{i}(f)$ peaks may shift and/or narrow depending on the model’s kinetic parameters, we use a metric proposed by Romano and coworkers [54] that quantifies the “selectivity” *S* for a specific range of frequencies, given by


(11) \begin{eqnarray*}
S_{i} = 1-\frac{\int _{f_{\rm {min}}}^{f_{\rm {max}}} {\rm FR}_{\rm {pulse}}^{i}(f) \:df}{[log(f_{\rm {max}})-log(f_{\rm {min}})]\: {\rm max}({\rm FR}_{\rm {pulse}}^{i}(f))},
\end{eqnarray*}

where *i* = 1, 2. *S*_*i*_ is also bounded in the range [0, 1]. This metric considers the area and the height of the *i*th target’s fold repression curve ${\rm FR}_{\rm {pulse}}^{i}$ in relation to the frequency range covered, and thus measures its frequency preference (see Fig. 2B).

The frequency value where ${\rm FR}_{\rm {pulse}}^{i}(f)$ peaks—i.e. the *i*th target’s “preferred frequency” f*—may also vary depending on model parameters, and we indicate it as


(12) \begin{eqnarray*}
f^{*}_{i} = {\rm argmax}({\rm FR}_{\rm {pulse}}^{i}(f))
\end{eqnarray*}

(see Fig. 2C). Note that since model M1 involves a single target, the index *i* will be omitted in the analysis. We would like to emphasize that these metrics are intended to compare periodic versus constant miRNA transcription in terms of relative target fold repression, rather than to indicate absolute levels of repression (whether strong or weak) exerted by miRNAs.

## Results

### Periodic miRNA synthesis can achieve higher fold repression than constant synthesis

To compare the fold repression generated by periodic and constant miRNA syntheses (i.e. by *σ*_pulse_ and *σ*_const_), we chose a fixed parameter set of model M1 with realistic kinetic rate values (see Supplementary Section S5), and we computed FR_const_ and FR_pulse_(*f*), in the timespan [0, *τ*] (see the “Materials and methods” section). Here, *τ* corresponds to the time needed by the target RNA to reach steady state in the constant synthesis case, i.e. *τ* = *t*_ss_: in this way, we compare the two average fold repression values generated in the target’s transient state under constant miRNA expression. An analysis of how the RNA equilibration time *τ* is related to the main timescales of the regulatory motif can be found in Supplementary Section S6.

**Figure 3.**
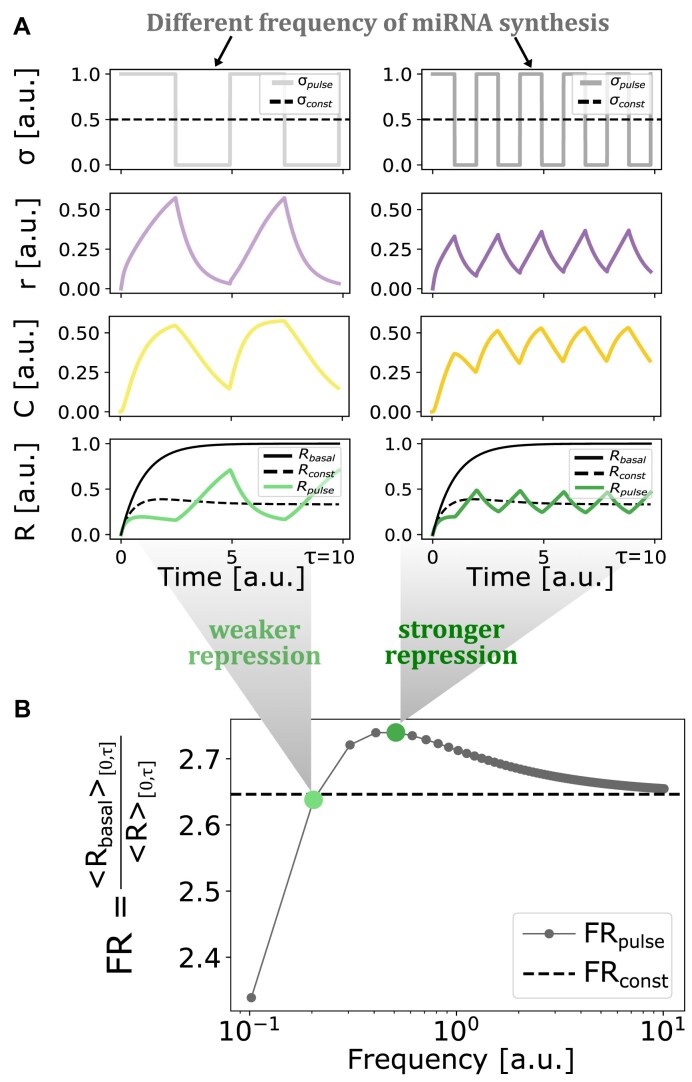
Periodic miRNA synthesis can generate stronger target repression than constant synthesis. (**A**) Example of temporal molecular concentrations resulting from two different input pulse frequencies in model M1. (**B**) Example of fold repression computed in the [0, *τ*] time interval. Dots along the solid line represent FR values resulting from different frequencies of periodic miRNA synthesis [FR_pulse_(*f*)]. Highlighted dots represent FR values resulting from the two frequencies of miRNA synthesis represented in panel c. The dashed line represents the FR value resulting from constant miRNA synthesis with identical miRNA-to-RNA relative dose (FR_const_).

We found that some frequencies of periodic miRNA synthesis lead to higher fold repression than constant synthesis (Fig. 3A and B). Note that for high frequencies the FR_pulse_(*f*) curve approaches FR_const_, showing that the fold repression achieved by closely spaced pulses of miRNA synthesis mimics the one resulting from a constant synthesis rate.

### Sensitivity analysis of model M1 identifies parameters relevant for frequency-dependent response

To investigate how parameter variations impact the frequency dependence of fold repression, 10^4^ parameter sets of model M1 were randomly selected using Latin Hypercube sampling [55]. Parameter ranges were estimated using data extracted from literature (see Supplementary Section S5), with the aim of covering a wide range of biologically plausible scenarios of RNA–miRNA interaction. Since nondimensionalized parameters are ratios of original dimensional parameters and thus can span orders of magnitude, a log-uniform sampling method was employed.

**Figure 4.**
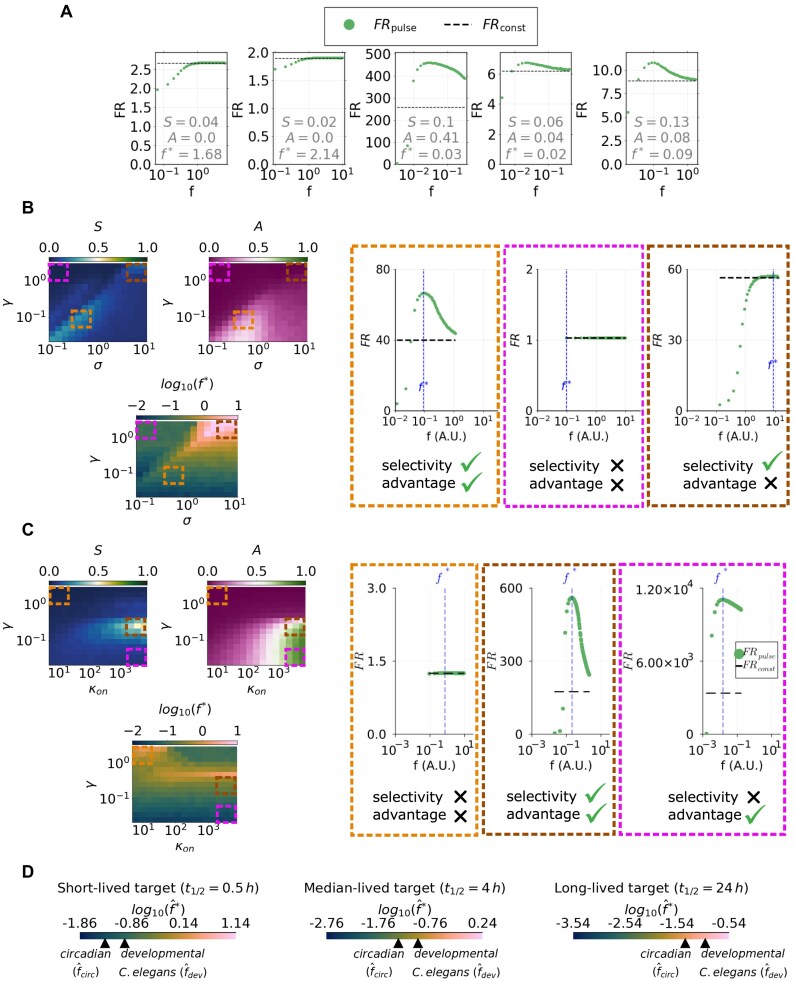
Frequency-dependent fold repression response depends on relative miRNA–target kinetics. (**A**) Random sampling of fold repression computed in the [0, *t*_ss_] interval for either constant or periodic miRNA input synthesis. Dots represent FR_pulse_(*f*) values, whereas black dashed lines represent FR_const_ values. Each subplot refers to a parameter set sampled randomly by Latin Hypercube sampling. For each case, advantage (*A*), selectivity (*S*), and preferred frequency (f*) values are reported in the plot. (**B**) Selectivity *S* (left heatmap), advantage *A* (middle heatmap), and preferred frequency f* (right heatmap) as a function of relative miRNA–RNA synthesis (*σ*) and degradation (*γ*) rates. Underlying plots represent fold repression curves corresponding to parameter values highlighted in heatmaps with dashed squares: dots represent FR_pulse_(*f*) values, whereas horizontal dashed lines represent FR_const_ values. The preferred frequency f* is indicated by the vertical dashed line. (**C**) Selectivity *S* (left heatmap), advantage *A* (middle heatmap), and preferred frequency f* (right heatmap) as a function of the relative miRNA–RNA degradation rate *γ* and the miRNA–RNA binding rate *κ*_on_. Underlying plots represent fold repression curves corresponding to parameter values highlighted in heatmaps with dashed squares: dots represent FR_pulse_(*f*) values, whereas horizontal dashed lines represent FR_const_ values. The preferred frequency f* is indicated by the vertical dashed line. (**D**) Color scales showing ranges of dimensional preferred frequency $\hat{f}^*$ for three values of target RNA half-life: 0.5, 4, and 24 h. Arrows indicate where the circadian miRNA frequency (≈1/24 h) and the miRNA frequency relative to *C. elegans* development (≈1/10 h) fall on the respective ranges.

For each parameter set, we evaluated FR_const_ and FR_pulse_(*f*) in the range of frequencies that ensure dose conservation in the timespan [0, *τ*], where *τ* is the target’s equilibration timescale under constant miRNA synthesis *σ*_const_ (see the “Materials and methods” section). See some examples of sampled FR_const_ and FR_pulse_(*f*) in Fig. 4A. For each sample, we subsequently computed the maximally preferred frequency f*, the selectivity *S*, and the advantage *A*. Then, to determine which parameters most greatly impact each metric, we computed the partial rank correlation coefficients [56] (see Table 1).

**Table 1.** Partial rank correlation coefficients between nondimensional M1 model parameters and the advantage *A*, the selectivity *S*, and the frequency of maximum fold repression f*

	*σ*	*κ*_on_	*κ*_off_	*α*	*β*	*γ*
f*	0.24	0.04	0.04	−0.20	0.09	0.79
*S*	0.13	0.25	−0.10	0.38	−0.25	−0.27
*A*	0.10	0.27	−0.14	0.60	−0.24	−0.57

The preferred frequency, i.e. f*, resulted mostly correlated with the relative miRNA–RNA degradation rate *γ*, showing that the reciprocal decay timescales of repressor and target primarily dictate the privileged timescale of miRNA synthesis pulses. Specifically, a faster decay of miRNA compared to the target results associated with higher preferred frequencies. This is consistent with the fact that an unstable miRNA requires temporal accumulation—which is enabled by higher synthesis frequencies—to achieve its maximal repression.

The analysis also showed that the scaled RNA decay in complex, *α*, and the relative miRNA/RNA degradation rate, *γ*, are highly correlated both with the advantage *A* and the selectivity *S*, followed by the binding constant *κ*_on_ and the scaled miRNA decay in complex, *β*. Precisely, *S* and *A* resulted positively correlated with *α*: we suggest that an enhanced target decay in complex amplifies the nonlinearity intrinsic to the modelled miRNA–target interaction, resulting in an increased fold repression and thus in higher advantage and selectivity. Instead, the similar but negative correlation values found for *A* and *S* with *γ* suggest a shrinking of such nonlinear amplification when the regulator is less stable than the target. In line with this interpretation, parameters *κ*_on_ and *β* present, respectively, positive and negative correlations with both *A* and *S*, consistent with the role that is played by these parameters in either amplifying or limiting miRNA-mediated repression in our model.

To highlight that nonlinear amplification drives the emergence of advantage and frequency selectivity, we also investigated through analytical and computational methods a simplified model that lacks nonlinear terms, showing that it does not exhibit these features (see Supplementary Section S7.1). Moreover, by incorporating nonlinearity, we could recover selectivity and advantage behaviours, although less pronounced than in our full model M1 (see Supplementary Section S7.2).

### Relative miRNA–RNA kinetics controls frequency-dependent response

To explore in more detail the role of influential parameters in model M1 in determining the dependence of fold repression on frequency, we computed FR_const_ and FR_pulse_(*f*) in the timespan [0, *τ*], where *τ* is the RNA equilibration time under constant miRNA synthesis, by varying pairs of parameters and keeping the remaining fixed to mean range values (see parameter estimation in Supplementary Section S5). In this way, we obtained the three metric quantities, i.e. advantage *A*, selectivity *S*, and preferred frequency f*, as functions of pairs of parameters (Fig. 4B and C, and Supplementary Figs S3, S4, and S5). Note that while the sampling described in the previous section provides insight into the global role played by each rate constant, these results capture the role of parameters in a typical range of miRNA and target kinetics—as here all rates are set to median values except for the two that vary at a time.

The paired variation of miRNA synthesis and degradation rates *σ* and *γ* showed that a trade-off between these parameters controls the target’s selectivity: high *S* values are either found for concomitantly low or concomitantly high *σ* and *γ* values (see Fig. 4B, orange and brown delimited areas, with the corresponding fold repression curves depicted below).

Similarly, *A* displays maximal values for concomitantly slow miRNA synthesis and degradation, i.e. low *σ* and *γ* (see orange delimited area in Fig. 4B), showing that a slowly but stably expressed miRNA leads to the highest advantage of periodic over constant regulator synthesis. However, with fast miRNA synthesis and degradation (brown delimited area in Fig. 4B), the advantage is lost, and the target’s preferred frequency f* is concomitantly shifted to higher values—a behaviour coherent with the fact that high-frequency pulses mimic constant synthesis rates (as shown by the corresponding FR_pulse_(*f*) curve approaching FR_const_).

If instead the regulator is both weakly expressed and unstable (low *σ* and high *γ*; pink delimited area in Fig. 4B), both advantage and selectivity are lost, with no repression in either the constant or in the periodic synthesis case.

Another interesting scenario emerges by concomitantly varying miRNA stability and its affinity to the target (i.e. varying kinetic rates γ and κ_*on*_): the maximal selectivity *S* emerges at a specific relative half-life of miRNA and target, and becomes more evident for high miRNA–target binding rates (see Fig. 4C, brown delimited area). This suggests that an optimal relative stability of regulator and target might make frequency preference emerge more clearly. The same parameter region also presents a high advantage *A*. However, *A* is always high below a certain relative half-life of regulator and target (see pink delimited area in Fig. 4C), even where selectivity is lost. Conversely, if the miRNA is both unstable and weakly interacting with its target, i.e. high *γ* and low *κ*_on_ (orange delimited area in Fig. 4C), both selectivity and advantage are lost. Note that here the preferred frequency f* takes higher values, consistent with a poorly expressed and weakly interacting miRNA privileging higher synthesis frequencies that allow its accumulation.

Overall, we found that the system’s synthesis and degradation rates control frequency preference behaviours in a trade-off-like manner, meaning that reciprocally balanced conditions of such parameters generate the highest selectivity and/or advantage. Instead, more constrained kinetic conditions are required for maximal selectivity and/or advantage when varying miRNA–RNA binding and unbinding rates.

Note that the emergence of a bell-shaped fold repression curve is expected to be more frequent in the observed time interval [0, *τ*]—which corresponds to the transient state of the constantly stimulated system—than in a timespan corresponding to its steady state (e.g. [*τ*, 2*τ*]): the later the time window of observation, the more privileged the higher frequencies—as they allow a cumulative effect of miRNA action. Indeed, if we compare fold repression responses computed in timespans [0, *τ*] and [*τ*, 2*τ*] for identical parameter values, we notice that the later stage prevalently presents high-pass filtering behaviours (see Supplementary Section S9).

### Response to circadian and developmental miRNA synthesis frequencies

To interpret our results in light of known periodic patterns of miRNA expression—such as the circadian and the *C. elegans* developmental rhythms—we examined how the system responds to these characteristic frequencies.

Since time is scaled by the target’s half-life, the dimensional frequency axis in our results varies with the target’s stability: $\hat{f}$ = $k_{R}^0f = \frac{{\rm ln}2}{t_{1/2}}f$, where $\hat{f}$ is the dimensional frequency with units h^−1^. We therefore chose three scenarios of target stability—short-lived (half-life *t*_1/2_ = 0.5 h), median-lived (*t*_1/2_ = 4 h), and long-lived (*t*_1/2_ = 24 h)—and for each case, we analyzed the system’s response to the circadian frequency ($\hat{f}_{\rm {circ}}\approx 1/24$ h^−1^) and the *C. elegans* developmental one—$\hat{f}_{\rm {dev}}\approx 1/10$ h^−1^, as a rough estimation from [10].

Figure 4D reports preferred frequency scales corresponding to the three stability cases, indicating where the circadian and developmental frequencies fall. By comparing such scales with preferred frequency values in Fig. 4B and C, we observed that depending on the target’s stability, the kinetic conditions where either $\hat{f}_{\rm {circ}}$ or $\hat{f}_{\rm {dev}}$ is preferred change, and thus also selectivity and advantage behaviours differ.

In the short- and median-lived target cases, $\hat{f}^{*}$ aligns with a 24-h period of miRNA synthesis $\hat{f}_{\rm {circ}}$, for instance, if the regulator is more stable than the target (low *γ*) (Fig. 4B). Similar observations hold if $\hat{f}^{*}$ corresponds to the developmental 10-h pulse timing $\hat{f}_{\rm {dev}}$, however, for slightly higher *γ*. In these conditions, selectivity *S* and advantage *A* can present high values (Fig. 4B, orange delimited area). This indicates that for targets with short enough lifetime—slightly shorter in the case of the 10-h cycle than the 24-h cycle—regulators expressed with developmental or circadian timing may not only enable dynamic control but also enhance average repression compared to a constantly expressed regulator.

Conversely, a long-lived target is maximally repressed by the circadian frequency $\hat{f}_{\rm {circ}}$ only under high *σ* and *γ* conditions (Fig. 4B, brown delimited area), where the advantage *A* is lost. Similarly, the regions defined by *γ* and *κ*_on_ in which long-lived targets favor circadian oscillations display low advantage and selectivity (Fig. 4C, orange delimited area). Comparable trends—albeit slightly shifted—are observed for the *C. elegans* developmental rhythm $\hat{f}_{\rm {dev}}$. Hence, for excessively stable targets, circadian and developmental miRNA synthesis timings lose their repressive benefit relative to constant expression.

These observations suggest that, for sufficiently unstable targets, developmental and circadian miRNAs may exert stronger average repression over time compared to constant synthesis, in addition to serving as mechanisms of dynamic regulation during development and day/night rhythmic control.

### Frequency-dependent response is affected by initial miRNA and RNA conditions

Examining the extent of repression due to constant versus pulsatile miRNA synthesis rate during the equilibration time means that transient dynamics may be captured. Therefore, the fold repression response may also be affected by initial concentrations of molecules. We thus fixed model M1 parameters to median values of log-uniform distributions in the estimated ranges (see Supplementary Section S5) and varied the starting miRNA and RNA concentrations. For each initial condition we evaluated FR_const_ and FR_pulse_(*f*) in the time interval [0, *τ*], with *τ* corresponding to the target’s equilibration time in the constant synthesis case. With this approach, we aim to mimic different situations where either the regulator or the target, or both, start being expressed after a specific cellular signal and interacting with the cognate species. In particular, we focus on four different initial conditions where: (i) both the miRNA and the target RNA start being synthesized from zero: *R*_0_ = 0 and *r*_0_ = 0; (ii) miRNA synthesis starts from zero and acts on a previously unrepressed RNA target, i.e. the initial RNA concentration corresponds to the noninteracting RNA steady state, which is always 1 due to nondimensionalization: *R*_0_ = 1 and *r*_0_ = 0; (iii) conversely, the initial miRNA concentration corresponds to its maximal steady state level in the absence of target interaction, i.e. *σ*/*γ*: *R*_0_ = 0 and *r*_0_ = *σ*/*γ*; and (iv) both the miRNA and the RNA are initially expressed to their noninteracting steady state levels, corresponding respectively to *σ*/*γ* and 1, and start interacting afterward: *R*_0_ = 1 and *r*_0_ = *σ*/*γ*. By varying initial miRNA and RNA conditions in the respective ranges *r*_0_ = [0, *σ*/*γ*] and *R*_0_ = [0, 1], we explore all four situations.

**Figure 5.**
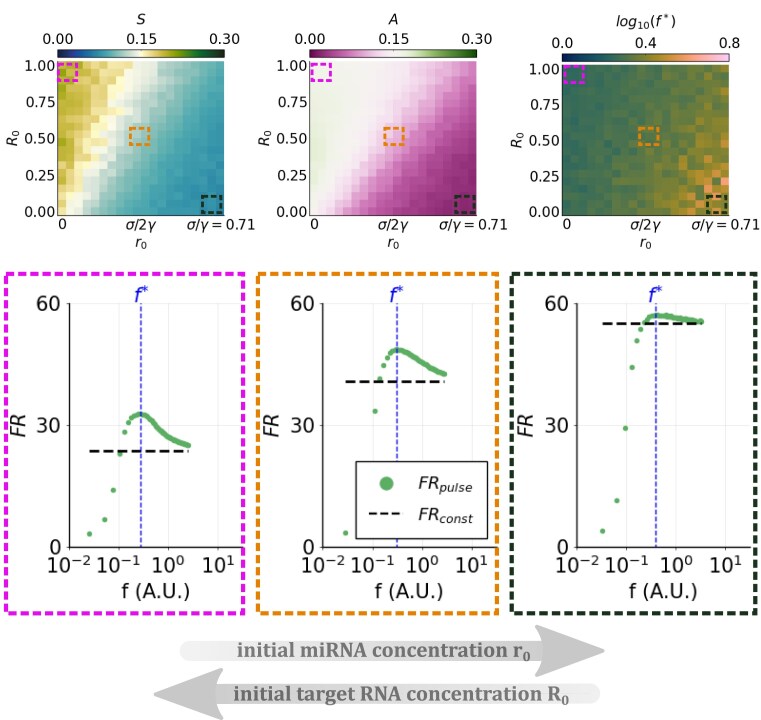
Frequency-dependent fold repression response depends on initial miRNA and RNA concentrations. Fold repression computed in the [0, *t*_ss_] interval for varying initial concentrations of miRNA and target RNA. Heatmaps represent selectivity *S* (left), advantage *A* (middle), and preferred frequency f* (right) as functions of initial miRNA (*r*_0_) and target RNA (*R*_0_) concentrations. Underlying plots represent fold repression curves corresponding to initial miRNA and RNA conditions highlighted in heatmaps with dashed squares of corrsponding color: dots represent FR_pulse_(*f*) values, whereas dashed lines represent FR_const_ values. The preferred frequency f* is indicated by the vertical dashed line.

We found that both selectivity *S* and advantage *A* increase with the initial RNA concentration and decrease with the initial miRNA concentration (Fig. 5A). Despite lower absolute values of fold repression, frequency preference is more pronounced if the target is initially abundant and conversely the miRNA is not yet present—as shown by the curves corresponding to different initial miRNA and target conditions (pink, orange, and black delimited areas in Fig. 5A). This means that if the target RNA is already expressed when the regulator starts being synthesized, its frequency preference behaviour is enhanced. Concomitantly, the target’s preferred frequency f* shifts to slightly higher values for high initial miRNA and low initial RNA concentrations, privileging temporal miRNA accumulation over oscillation.

These results indicate that the selective response to a periodic pattern of miRNA synthesis might be more relevant if the regulator acts on a constitutively expressed target.

### Sensitivity analysis of model M2 shows that competitor RNA kinetics affects a target’s frequency-dependent response

**Figure 6.**
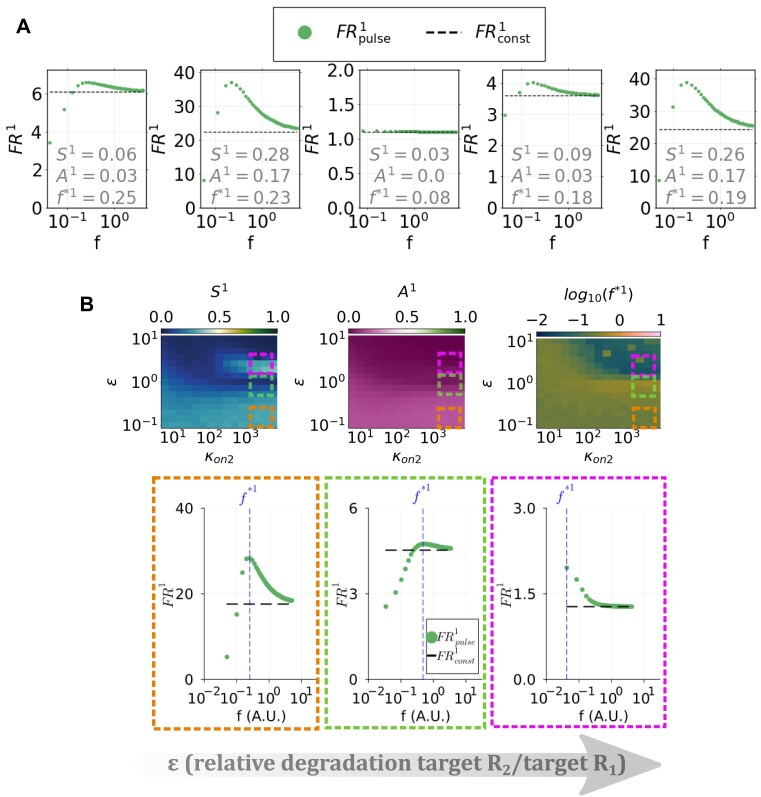
Competitor RNA kinetics affects frequency-dependent fold repression response. (**A**) Random sampling of target *R*_1_ fold repression computed in the $[0, t_{{\rm ss}_1}]$ interval for constant and periodic miRNA synthesis. Dots represent ${\rm FR}_{\rm {pulse}}^{1}(f)$. Dashed lines represent ${\rm FR}_{\rm {const}}^{1}$. Each subplot refers to a set of parameters of the competitor target, i.e. *R*_2_, sampled randomly by Latin Hypercube sampling. For each case, values of target *R*_1_ advantage (*A*^1^), selectivity (*S*^1^), and preferred frequency (f^*1^) are reported in the plot. (**B**) Fold repression selectivity *S*^1^ (left plot), advantage *A*^1^ (middle plot), and preferred frequency f^*1^ (right plot) as functions of the miRNA–RNA binding rate, *κ*_on_, and the competitor’s decay rate relative to *R*_1_, *ε*. Underlying plots represent fold repression curves corresponding to parameter values highlighted in heatmaps with dashed squares of corresponding color: dots represent FR_pulse_(*f*) values, whereas dashed lines represent FR_const_ values. The preferred frequency f^*1^ is indicated by the vertical dashed line.

We are interested in studying how the kinetics of a competitor can affect the frequency-dependent behaviour of a target RNA. We thus performed a random sampling of the second target’s parameters in model M2, and for each sample, we computed fold repression for the first target. To this purpose, we fixed parameters relative to target *R*_1_ and we randomly sampled parameters of the competitor *R*_2_, i.e. $\kappa _{{\rm on}_2}$, $\kappa _{{\rm off}_2}$, *α*_2_, *β*_2_, *δ* and *ε*, using Latin Hypercube sampling [55]. Fixed parameters correspond to median values of the log-uniformly distributed values in ranges estimated for model M2, whereas for the remaining parameters we drew 10^4^ sets from log-uniform distributions in the estimated ranges (see Supplementary Sections S5 and S10). Then, we evaluated ${\rm FR}_{\rm {const}}^{1}$ and ${\rm FR}_{\rm {pulse}}^{1}(f)$ in the timespan [0, *τ*], with *τ* corresponding to the first target’s equilibration time, for each permitted frequency (see the “Materials and methods” section). In this way, we computed the three metric quantities for the *R*_1_ target, i.e. the maximally preferred frequency f^*1^, the selectivity *S*^1^, and the advantage *A*^1^, associated with each sampled parameter set of the competitor RNA *R*_2_. Figure 6A shows examples of target *R*_1_ fold repression as a function of frequency for randomly selected parameter sets of the competitor target *R*_2_.

To determine how the parameters of the competitor impact the frequency-dependent behaviour of the first target, we calculated partial rank correlation coefficients [56] of f^*1^, *S*^1^, and *A*^1^ with each sampled parameter. Correlation results are summarized in Table 2.

**Table 2.** Partial rank correlation coefficients between parameters of competitor target RNA *R*_2_ and metric quantities relative to the first target *R*_1_: fold repression advantage (*A*^1^), fold repression selectivity (*S*^1^), and frequency of maximum fold repression (f^*1^)

	$\kappa _{{\rm on}_2}$	$\kappa _{{\rm off}_2}$	*α*_2_	*β*_2_	*δ*	*ε*
*A* ^1^	−0.01	0.11	0.53	−0.61	−0.08	−0.51
*S* ^1^	0.01	0.09	0.51	−0.62	−0.13	−0.48
*f* ^*1^	−0.27	0.04	0.10	0.48	0.22	−0.21

We found that both *S*^1^ and *A*^1^ are negatively correlated with β_2_ and positively with α_2_: a faster miRNA decay in complex with the competitor reduces the first target’s possibility to be repressed, whereas a faster complex-mediated decay of the competitor causes the opposite effect. Interestingly, both metrics are also anticorrelated with the parameter *ε*, showing that selectivity and advantage of target *R*_1_ are enhanced in case it is less stable than the competitor (low *ε*): we interpret this as due to the fact that a reduced amount of target makes it more easily inhibited by miRNA action. Note that since parameters of *R*_1_ are fixed to median range values, these results capture the global role of competitor kinetics in a scenario where *R*_1_ represents a typical target RNA and might vary for a target with different kinetics.

On the other hand, we found that the preferred frequency value of target *R*_1_ (i.e. f^*1^) is strongly correlated with *β*_2_ and $\kappa _{{\rm on}_2}$. Specifically, a faster miRNA degradation in complex with target *R*_2_ (i.e. a higher value of *β*_2_) leads the first target to prefer higher frequencies, as shown by the positive correlation value. This effect is in line with observations emerging from the analysis of model M1: a reduced availability of miRNA causes preference for higher synthesis frequencies. Instead, f^*1^ is negatively correlated with the competitor’s rate of binding to the miRNA $\kappa _{{\rm on}_2}$, meaning that a strongly interacting competitor shifts the first target’s preference to lower frequency pulses. Similar observations stem from correlation results obtained for the competitor’s synthesis and decay rates relative to *R*_1_, i.e. *δ* and *ε*: a faster synthesis of *R*_2_ causes a reduced availability of the miRNA to the first target, thus favoring again higher synthesis frequencies that lead to the regulator’s accumulation. Conversely, a faster decay of target *R*_2_ shifts the preference of *R*_1_ to lower frequencies of miRNA synthesis, as displayed by the negative correlation between *ε* and f^*1^.

Overall, these results indicate that a target RNA’s response to miRNA synthesis pulses of different frequencies is determined not only by the kinetic features of the target itself but also by those of competitors that share the same regulator. This suggests that by pairing targets with appropriate kinetics, one might be able to tune each one’s preference to a specific temporal pattern of miRNA expression.

### Competitor RNA degradation and binding kinetics shapes a target’s frequency-dependent response

**Figure 7.**
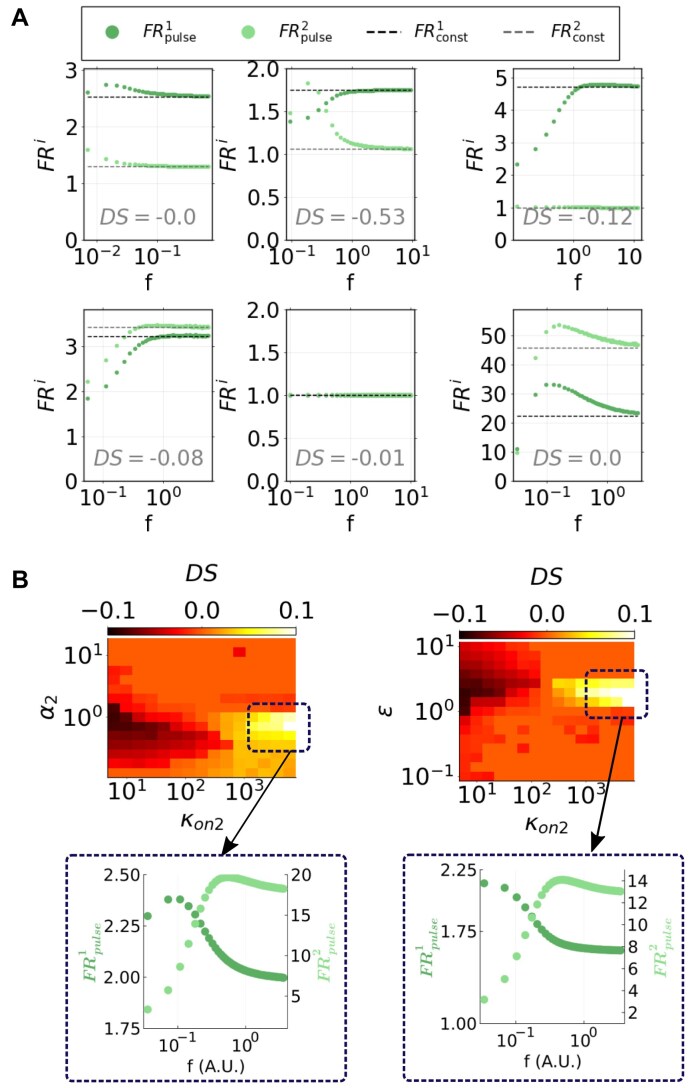
Periodic miRNA synthesis enables selective target regulation. (**A**) Random sampling of fold repression of the two competing targets *R*_1_ and *R*_2_ in the $[0, t_{{\rm ss}_1}]$ interval for constant and periodic miRNA synthesis. Dark and light green dots represent, respectively, ${\rm FR}^{1}_{\rm {pulse}}(f)$ and ${\rm FR}^{2}_{\rm {pulse}}(f)$. Black and gray dashed lines represent, respectively, ${\rm FR}^{1}_{\rm {const}}$ and ${\rm FR}^{2}_{\rm {const}}$. Each subplot refers to a randomly sampled set of parameters. For each case, the value of differential selectivity (DS) is reported in the plot. (**B**) Differential selectivity DS as a function of parameter pairs. The left heatmap represents DS as a function of the miRNA binding rate of target *R*_1_, $\kappa _{{\rm on}_2}$, and its degradation rate in complex, α_2_; the underlying plot represents fold repression curves ${\rm FR^{1}_{pulse}}$ and ${\rm FR^{2}_{pulse}}$ resulting from parameter values highlighted in the heatmap with a dashed square. The right heatmap represents DS as a function of the miRNA–RNA binding rate of target *R*_2_, $\kappa _{{\rm on}_2}$, and its decay rate relative to *R*_1_, *ε*. The underlying plot represents fold repression curves ${\rm FR}^{1}_{\rm {pulse}}$ and ${\rm FR}^{2}_{\rm {pulse}}$ resulting from parameter values highlighted in the heatmap with a dashed square.

To further explore the role of competitor RNA kinetics in shaping a target’s frequency-dependent response, we computed ${\rm FR}_{\rm {const}}^{1}$ and ${\rm FR}_{\rm {pulse}}^{1}(f)$ in the timespan [0, *τ*], *τ* corresponding to the equilibration time of *R*_1_, for different values of parameters of *R*_2_, keeping the remaining fixed to mean range values. From these, we obtained metrics relative to the first target, i.e. the selectivity *S*^1^, the advantage *A*^1^, and the preferred frequency f^*1^, as functions of pairs of competitor’s parameters (Fig. 7B and Supplementary Figs S7, S8, and S9).

Note that since rate constants are all fixed to median values except for each varying rate pair of the competitor, these results capture the role of these parameters restricted to the case where the two targets and their shared regulator all present typical kinetic features.

In line with parameter sampling results, we found that if the competitor is more stable than the first target (i.e. low *ε*), the latter presents higher selectivity and advantage, as shown by *S*^1^ and *A*^1^ values (Fig. 7B, orange delimited area). If the competitor’s miRNA binding rate $\kappa _{{\rm on}_2}$ is larger than that of the first target, a high *S*^1^ area emerges also where *R*_2_ is less stable than *R*_1_ (7B, pink delimited area)—however, with significantly lower fold repression values, as shown by the corresponding curve below. Here, the first target prefers lower frequency values—i.e. behaves as a low-pass filter—consistent with its slower kinetics given by its stability. Note that if instead the two targets have similar stability, the selective behaviour is less pronounced (green delimited area). This indicates that when stability is skewed in favor of one of the two target RNAs, frequency preference behaviours might emerge more evidently.

These findings suggest that tuning the relative degradation kinetics of distinct targets and their affinities of binding to a shared regulator might allow them to reciprocally modulate their frequency-dependent responses.

### Sensitivity analysis of model M2 shows that the relative kinetics of competing target RNAs enables their exclusive frequency-driven regulation

We aim to understand whether different RNA targets sharing the same miRNA could be regulated through distinct pulse frequencies, and how the occurrence of this exclusive regulation scenario depends on their relative kinetics.

To search for conditions where the two competing targets might show a preference for different input frequencies, we sampled their fold repression response resulting from constant and periodic miRNA synthesis. To this purpose, we explored the complete parameter space—including parameters of both targets—by Latin Hypercube sampling [55], drawing 10^4^ sets from log-uniform distributions in the estimated ranges (see Supplementary Sections S5 and S10). For each parameter set, we evaluated ${\rm FR}_{\rm {pulse}}^{1}(f)$, ${\rm FR}_{\rm {pulse}}^{2}(f)$, ${\rm FR}_{\rm {const}}^{1}$ and ${\rm FR}_{\rm {const}}^{2}$ in the time interval [0, *τ*], for each permitted frequency (see the “Materials and methods” section). Here, *τ* corresponds to the equilibration timespan of target *R*_1_ under constant miRNA synthesis: in this way, we address the frequency-dependent behaviour of both RNA species in the same time interval, which is characteristic to the repression of one of them. Randomly sampled examples of fold repression for both targets are shown in Fig. 7A.

To address situations where the two targets respond maximally to distinct frequencies, we defined the metric quantity “differential selectivity” DS, which captures cases where their selectivities *S*^1^ and *S*^2^ are concomitantly high but their preferred frequencies are distant as


(13) \begin{eqnarray*}
{\rm DS} = \frac{2}{\frac{1}{S^1} + \frac{1}{S^2}} \cdot [\log _{10}(f^{*2}) - \log _{10}(f^{*1})].
\end{eqnarray*}

The harmonic mean of selectivities, given by $\frac{2}{\frac{1}{S^1} + \frac{1}{S^2}}$, privileges cases where both *S*^1^ and *S*^2^ are high, and penalizes cases where only one of the two targets presents a high selectivity. Given the factor log_10_(f^*2^) − log_10_(f^*1^), we expect negative or positive DS values depending on the relative position of two target RNAs’ preferred frequencies.

Thus, to investigate how the exclusive regulation of the two targets in frequency is affected by their relative kinetics, we calculated partial rank correlation coefficients (see results in Table 3) between our metric DS and the following parameter ratios: $\frac{K_1}{K_2} = \frac{\kappa _{{\rm on}_2}}{\kappa _{{\rm on}_1}} \cdot \frac{\kappa _{{\rm off}_1}}{\kappa _{{\rm off}_2}}$, $\frac{\alpha _2}{\alpha _1}$, $\frac{\beta _2}{\beta _1}$, δ and ε.

**Table 3.** Partial rank correlation coefficients between parameter ratios of the two target RNAs and the differential selectivity DS

Parameter	*K*_2_/*K*_1_	*α*_2_/*α*_1_	*β*_2_/*β*_1_	*δ*	*ε*
DS	0.40	−0.19	−0.04	0.06	−0.12

Interestingly, we found that DS is mostly correlated with the relative dissociation constant of the two targets, *K*_2_/*K*_1_, showing that the separation of miRNA–target interaction timescales is important for the exclusive target repression controlled by frequency. Moreover, we found both the ratio between parameters *α*_2_ and *α*_1_ as well as the relative decay of the two targets, *ε*, slightly anticorrelated with DS.

This result highlights the possibility of exclusive target regulation driven by periodic miRNA synthesis, and suggest that experimentally tunable kinetic rates—such as the relative miRNA–target affinity of the two competitor RNAs—can be adapted to achieve this exclusivity.

### Target regulation by distinct frequencies is enabled by hierarchical binding affinities and degradations

Given the correlation of relative kinetic rates of the two targets with the differential selectivity DS, we explore the behaviour of such metric by varying relevant parameters in pairs. Since all rate constants are fixed to the estimated median values except for the varying parameter pair, these results refer to situations where both targets and the miRNA have typical kinetic features.

We thus computed DS as a function of $\kappa _{{\rm on}_2}$ and α_2_, keeping the remaining parameters fixed to the estimated median range values (Supplementary Sections S5 and S10). In this way we found that when target *R*_2_ has either lower or higher miRNA affinity than target *R*_1_ (i.e. low or high $\kappa _{{\rm on}_2}$), a specific range of α_2_ values gives the greatest negative or positive values of DS (Fig. 7B, left heatmap). This suggests that targets whose affinities to the regulator and whose complex-mediated stabilities differ enough, are likely to exhibit preference for distinct frequencies.

Similarly, by varying $\kappa _{{\rm on}_2}$ and the relative decay rate of the two targets ε, we observed that in a narrow range of *ε* values the differential selectivity DS takes the highest absolute values, either negative or positive (Fig. 7B, right heatmap). Note that this behaviour appears in a range of *ε* (or *α*_2_) values where the kinetic rate of the competitor does not differ greatly from that of the first target. These results suggest that small differences in the half-lives or in the decay induced by miRNA binding of the two targets can guarantee their exclusive regulation in frequency, as long as their affinities to the regulator are dissimilar enough.

These insights might provide suggestions for an experimental kinetic tuning that allows targets sharing the same miRNA to respond exclusively to regulatory stimuli of specific frequency: we suggest that such tuning may focus on the binding affinity between an miRNA and its targets and/or on the relative decay kinetics of the two competitors.

## Discussion

### Tuning miRNA and RNA kinetics to reveal frequency-dependent repression

The role of oscillatory gene expression has been extensively studied in the context of transcriptional regulation [1, 2], where periodic temporal concentrations of molecules have been shown to underlie the functionality of genetic pathways. Despite the tight connection between transcriptional and posttranscriptional regulatory interactions, the potential implications of periodic miRNA expression have been largely disregarded. Nevertheless, some experimental findings revealed the existence of periodically expressed miRNAs [35–42].

In this work, we theoretically investigated the implications of periodic miRNA synthesis by focusing on its comparison with constant miRNA synthesis. We considered a dynamical model wherein an miRNA binds an RNA, resulting in a complex that can undergo unbinding or degradation through two pathways: either recycling the miRNA and degrading the RNA or vice versa. We estimated the system’s timescale as the time it takes the RNA to reach steady state when fed with a constant miRNA synthesis rate. This enabled us to make a comparison between constant and periodic synthesis in inducing target RNA repression.

We showed that periodic miRNA synthesis can achieve a higher average fold repression than constant miRNA synthesis. This result directly aligns with the concept of oscillatory stimuli being used to differentiate circuit behaviours, as done in [9], and in line with [3], provides a further example of how biological systems exploit pulse frequencies for regulatory purposes.

According to our analysis, the maximal advantage of periodic over constant miRNA synthesis is found when the miRNA presence is overall stronger (i.e. longer half-life relative to the target, low degradation in complex). This is due to the inherent nonlinearity of the system, which in favorable conditions amplifies the repression gap between periodic and constant miRNA synthesis. Our additional analyses of simplified miRNA–target interaction models (Supplementary Section S7) support nonlinearity as a necessary element for the emergence of frequency preference behaviours. To highlight that nonlinear amplification drives the emergence of advantage and frequency selectivity, we analyzed a simplified model lacking nonlinear terms using both analytical and computational methods. This analysis confirmed that the model does not exhibit these features (Supplementary Section S7.1). Furthermore, by incorporating nonlinearities, we were able to recover selectivity and advantage behaviours (Supplementary Section S7.2), as also shown in Supplementary Fig. S2 where the simplified model retains frequency preference but with reduced advantage compared to the full model.

Thus, our results suggest that revealing a repressive advantage of periodic over constant miRNA synthesis might be less challenging by combining stable miRNAs and unstable target RNAs and thus privileging messenger RNAs (mRNAs) over long non-coding RNAs (lncRNAs) and circular RNAs (circRNAs)—which are known for their higher stability. This was also supported by our focused analysis of the circadian and *C. elegans* developmental rhythms, which revealed median-/short-lived targets as potentially exhibiting frequency preference and repressive advantage of such periodic timings over constant miRNA synthesis. Some periodically expressed miRNAs [36–38, 41, 62] are known to target relatively unstable RNAs [57–61]] and thus could fall into this behaviour. Table 4 reports examples of such target RNAs associated with circadian miRNAs, either experimentally observed or computationally predicted by TargetScan.

**Table 4.** Targets with respective half-lives and their associated circadian miRNAs

Target RNA	Half-life (*t*_1/2_)	Circadian miRNAs
CLOCK	3–4 h [57, 58]	miR-96 [36]
		miR-182 [36]
		miR-17-5p [41]
XBP1	2 h [58]	miR-219-1 [37]
UBP1	3.4 [58]	miR-219-1 [37]
MRPL20	7.9 h [58]	miR-132 [37]
MECP2	5 h [58, 59]	miR-132 [37]
dbt	1.4–6.7 h [60]	dme-miR-263a [38]
clk	0.5 h [61]	dme-miR-263a [38]
		dme-miR-263b [38]
mCRY1	4.4 h [59]	miR-185 [62]

Interestingly, we showed that a high strength of binding between the miRNA and its target generates optimal advantage and frequency selectivity when the two are produced to equimolar concentrations (i.e. *σ* = 10^0^). This effect could be due to the maximal nonlinearity of target response in this condition [53]. For experimental aims, this suggests that it might be possible to considerably strengthen the repression of a target RNA with high affinity to the miRNA by using periodic synthesis of the regulator—if their relative stoichiometries are properly tuned. We thus speculate that an artificial target with multiple miRNA-binding sites might enable detecting this effect.

Our study addresses inhibitory effects of miRNAs by focusing on a fold repression response—an experimentally accessible observable that is of particular interest especially when dealing with mRNAs, whose levels are reduced by miRNA action. However, the biochemical reactions involved in the employed model enable as well the investigation of scenarios involving other kinds of RNAs that are not necessarily affected by the interaction with miRNAs in terms of repression. For instance, scenarios where miRNA–RNA binding does not result in an enhanced decay of the RNA but conversely of the regulator [48, 63] are included in our model.

### Frequency-dependent repression might be shaped by upstream constraints to miRNA and target RNA concentrations

Our results highlighted that the initial concentrations of target RNA and miRNA play a role in determining the response of the system to a frequency of regulator synthesis. This can be put in relation with the recently demonstrated “burden effect”—i.e. the coupling between otherwise independently produced molecules that results from the intrinsic limitation of synthesis machineries. Mathematical modelling predicted, for instance, that protein synthesis might be constrained when ribosomes are saturated by other species being translated [64]. In line with these predictions, it was experimentally revealed that transiently expressed genes compete for finite transcriptional and translational resources [65, 66]. These effects might thus potentially affect frequency preference behaviours by posing an upstream constraint on miRNA and target RNA concentrations.

### Frequency-dependent repression is affected by cross talk between target RNA species

We explored a few implications of competition between RNA targets for miRNA binding. By adding a competitor RNA with proper kinetics, we observed that tuning of its rate constants can not only affect the repression of the first target driven by periodic miRNA synthesis, but most interestingly, it can shift its preference for a specific range of frequencies. The idea that not only the single miRNA–target interaction but also the entire competitive system including the multiple species at play might be kinetically tuned to achieve a precise target response is coherent with the well-known ceRNA hypothesis [67]. For instance, our predictions show that the frequency-dependent response of a target in the presence of a competitor mainly depends on their relative half-lives: this suggests that the observed concomitant targeting of RNA types that differ in their stability—such as circRNAs, lncRNAs, and mRNAs—might represent a way to precisely tune repression driven by periodic miRNA expression. It is known, for instance, that both lncRNAs and circRNAs can serve as miRNA sponges that indirectly regulate miRNA target genes [68, 69]: according to our analysis, their action might be able to shift and/or sharpen the frequency-dependent response of a target in a way that depends on their degradation kinetics.

### miRNA–RNA binding affinity as driver of differential frequency-dependent target regulation

Our finding that a large gap between affinities of RNAs sharing the same miRNA can lead to their exclusive repression in the same timespan indicates that specific input frequencies might enable the selective regulation of single targets despite the presence of multiple RNA species in the network. Again, miRNA–target binding kinetics emerges as a crucial element for the tuning of frequency-driven regulation. This scenario would be compatible with the fact that, in principle, a single miRNA can target RNA species with highly variable affinity—as the degree of complementarity is determined by the binding site sequence(s) located on the specific target transcript [20]. Moreover, the moderate differences in target half-lives, which are predicted to generate exclusive repression are compatible with differences in degradation kinetics detected among the main RNA types targeted by miRNAs (lncRNAs, circRNAs, mRNAs). For instance, the half-lives of circRNAs were shown to be on average 2.5 times longer than those of mRNAs [70]—a difference compatible in our predictions with the possibility of exclusive regulation in frequency.

### Potential model extensions

The models proposed in this study describe the interaction between miRNAs and their target RNAs by accounting for essential processes at play, condensing complex biochemical interactions into simplified, effective reactions. However, more intricate models that delve deeper into miRNA biogenesis and/or into biochemical details of miRNA-mediated repression might provide additional insight into the role of periodicity in such regulatory interactions. Potential model extensions might involve intermediate miRNA processing steps that start from the synthesis of primary transcripts (pri-miRNAs) and result in the incorporation of the mature miRNA molecule into the RNA-induced silencing complex, which mediates interaction with the target [71].

Interestingly, the presence of multiple miRNA-target binding sites was predicted to generate natural oscillations in the target’s concentration [47] and lead to high-order multistability [72]. Therefore, extending our models to include cooperative miRNA binding might reveal whether and how target oscillations interact with the forced ones given by periodic miRNA synthesis.

Besides adding complexity to the interaction network, one might also consider different and more realistic approaches to model the periodicity of miRNA expression. Thus, for the sake of completeness, we show that using a sinusoidal miRNA synthesis rate instead of a square wave does not qualitatively alter our results (Supplementary Section S12).

However, as the vast majority of RNA species targeted by miRNAs are protein-coding [73], the inclusion of a protein product represents perhaps one of the most meaningful extensions to our model. In the protein-coding context, miRNAs serve as fine-tuners of protein expression [20]. Therefore, whether periodicity of miRNA synthesis might play a regulatory role also at the protein level is a meaningful question. We tackle this issue by addressing how frequency preference behaviours of miRNA-mediated repression translate from the RNA level to the protein level, extending model M1 with the addition of a protein product species (Supplementary Section S13). With this extended model, we show that frequency preference behaviours at the protein level can mirror those detected at the RNA level with varying degrees of fidelity, depending on the protein’s kinetic properties and on the timespan considered. Specifically, we show that protein products with faster synthesis and degradation are more likely to display frequency-dependent repression. This suggests that rapid enough kinetics might allow the detection of frequency preference behaviours also at the protein level.

Another natural extension would be the embedding of the basic miRNA-target interaction into more intricate motifs where oscillating miRNAs are incorporated, such as iFFLs. The *C. elegans* iFFL studied in [10], where the miRNA *lin-4* dampens oscillations of its target *lin-14*, is a relevant example. As this particular iFFL displays synchronous periodicity of miRNA and target expression under the control of a hypothetical master regulator, we briefly explore the possibility of frequency-dependent behaviours in such a scenario (Supplementary Section S14). Our analysis shows that when miRNA and RNA are transcribed in synchronous pulses, the target’s fold repression and the dampening of its oscillations can exhibit opposed frequency preference behaviours: at an optimal input frequency, the target RNA exhibits maximal reduction of its oscillations while maintaining the mildest repression extent. This preliminary finding—which warrants further investigation—aligns with studies on miRNA-mediated iFFLs, which support their role in buffering fluctuations while exerting moderate repression on their targets [74]. We thus speculate that specific frequencies might have been selected within periodically forced iFFLs thanks to their oscillation dampening potential.

When adding a second, independently transcribed target regulated by the same miRNA, we observe opposite frequency-dependent repression patterns: the iFFL-embedded target shows a repression minimum, while the second target displays a maximum at similar frequencies. At this point, miRNA repression of the iFFL target is less effective in reducing mean expression but maximally dampens fluctuations while strongly repressing the second target’s mean expression. More broadly, it is possible that frequency plays a functional role within composite motifs beyond the simple miRNA–target interaction—where these regulators are typically involved.

### Potential roles of noise in miRNA-mediated regulation driven by frequency

Our study is still limited by the use of a deterministic framework, which excludes the role of noise from our analyses—an element thought to be central in miRNA-mediated regulation. Indeed, theoretical and experimental studies suggested that the action of miRNAs might contribute to buffering gene expression noise, thereby working as fine tuners of genetic regulation [75, 76]. This idea is supported by the pervasive presence of miRNA-mediated motifs—for instance, incoherent feedforward loops and toggle switches [74, 77]—which have been both theoretically [74, 78, 79] and experimentally [10, 80] shown to decrease target variability. However, it was also shown that miRNAs may amplify their targets’ noise when embedded in other kinds of regulatory circuits, and cells exploit this expression variability to commit to different developmental programs [81–83]. Given the tight connection between miRNAs and the managing of gene expression noise, it remains, for instance, to be determined whether target RNAs regulated by a periodically expressed miRNA might respond in a frequency-dependent manner not only at a mean value level but also in terms of variability. Moreover, given that fluctuations of targets regulated by the same miRNA can result as coupled, it would be of interest to understand how this crosstalk is affected by frequency. The interplay between stochasticity and oscillation has attracted particular interest in the broader context of genetic regulation, where it has been predicted that the response of a nonlinear system to a weak periodic input signal can be amplified by the addition of optimal levels of noise. It is speculated that this phenomenon, also known as “stochastic resonance”, might be able to drive the switching between distinct states of gene expression [84, 85]. Interestingly, it was observed that the periodic binding and unbinding of an siRNA to mRNA in a live cell displays stochastic resonance [86]. Overall, it would be of interest to investigate whether stochasticity might influence the repression of RNA targets driven by periodic miRNA synthesis in a biologically meaningful way.

## Supplementary Material

gkaf867_Supplemental_File

## Data Availability

No new data were generated or analyzed in support of this research.
