# Supplementary material for: The advantage of periodic over constant signalling in microRNA-mediated regulation
Source: Nucleic Acids Res. 2025 Sep 9;53(17):gkaf867. doi: 10.1093/nar/gkaf867 (PMC12418391; doi:10.1093/nar/gkaf867)
Supplement: gkaf867_Supplemental_File [file gkaf867_supplemental_file.pdf]

Supplementary Data
The advantage of periodic over constant signalling in microRNA-mediated regulation

Ferro Elsi [1,2], Szischik Candela L [3,4], Ventura Alejandra C [3,4], and Bosia Carla [1,2]

[1] *Department of Applied Science and Technology, Politecnico di Torino, Corso Duca degli Abruzzi 24, Torino, 10129, Italy.*
[2] *IIGM Foundation - Italian Institute for Genomic Medicine, c/o IRCCS - SP 142 km 3,95, Candiolo, 10060, Torino, Italy.*
[3] *Universidad de Buenos Aires, Facultad de Ciencias Exactas y Naturales, Departamento de Física, Ciudad Universitaria, 1428, Buenos Aires, Argentina.*
[4] *Instituto de Fisiología, Biología Molecular y Neurociencias (IFIBYNE UBA-CONICET), Consejo Nacional de Investigaciones Científicas y Técnicas Argentina, Universidad de Buenos Aires.*

1. M1 model description and nondimensionalization

To achieve our goals, we closely follow the framework presented in [1] for describing miRNA-RNA interactions, with only minor modifications introduced where explicitly noted. This model considers a single miRNA and RNA species, both synthesized through transcription. The two species can bind to form a complex, and each of them can be degraded both when unbound and when in complex independently. The chemical reactions included in the model are:

$$\varnothing \xrightarrow{S_R} \hat{R} \;,\; \hat{R} \xrightarrow{k_R^0} \varnothing \;,\; \varnothing \xrightarrow{S_r} \hat{r} \;,\; \hat{r} \xrightarrow{k_r^0} \varnothing \;,\; \hat{R} + \hat{r} \xrightarrow{k_{on}} \hat{C} \;,\; \hat{C} \xrightarrow{k_{off}} \hat{R} + \hat{r} \;,\; \hat{C} \xrightarrow{k_r^1} \hat{R} \;,\; \hat{C} \xrightarrow{k_R^1} \hat{r} \tag{1}$$

where $\hat{R}$, $\hat{r}$ and $\hat{C}$ represent respectively the RNA, the miRNA and the RNA-miRNA complex. These names refer both to the molecular species and to their concentrations.
The first two reactions represent respectively RNA synthesis and degradation, which occur with rate constants S_R and k_R^0. Similarly, the third and fourth reactions describe miRNA synthesis and degradation along with their rates S_r and k_r^0. The next two reactions represent miRNA-RNA binding and dissociation, whose rate constants are k_{on} and k_{off}. The last two reactions describe respectively miRNA and RNA degradation when the two species are in complex, occurring at rates k_r^1 and k_R^1; these reactions imply that the species that does not undergo degradation - either the miRNA or the RNA - is recycled back into the system, and are thus often called *recycling reactions*.
Assuming the law of mass action kinetics, we describe the dynamics of the three molecular species concentrations by the following system of ordinary differential equations (ODEs):

$$\begin{cases} \frac{d\hat{R}}{d\hat{t}} = S_R - k_{on}\hat{R}\hat{r} + k_{off}\hat{C} - k_R^0\hat{R} + k_r^1\hat{C} \\ \frac{d\hat{r}}{d\hat{t}} = S_r - k_{on}\hat{R}\hat{r} + k_{off}\hat{C} - k_r^0\hat{r} + k_R^1\hat{C} \\ \frac{d\hat{C}}{d\hat{t}} = k_{on}\hat{R}\hat{r} - k_{off}\hat{C} - k_R^1\hat{C} - k_r^1\hat{C} \end{cases} \tag{2}$$

To nondimensionalize the ODEs, we deviate from the approach used in [1] and instead apply the following change of variables:

$$\hat{t} = \frac{t}{k_R^0} \;,\; \hat{R} = \frac{RS_R}{k_R^0} \;,\; \hat{r} = \frac{rS_R}{k_R^0} \;,\; \hat{C} = \frac{CS_R}{k_R^0} \;. \tag{3}$$

That yield the dimensionless ODEs:

$$\begin{cases} \frac{dR}{dt} = 1 - \kappa_{on}Rr + \kappa_{off}C - R + \beta\gamma C \\ \frac{dr}{dt} = \sigma - \kappa_{on}Rr + \kappa_{off}C - \gamma r + \alpha C \\ \frac{dC}{dt} = \kappa_{on}Rr - \kappa_{off}C - \alpha C - \beta\gamma C \end{cases} \tag{4}$$

where dimensionless parameters are ratios of original parameters:

$$\sigma = \frac{S_r}{S_R} \,,\, \gamma = \frac{k_r^0}{k_R^0} \,,\, \kappa_{on} = \frac{k_{on}S_R}{{k_R^0}^2} \,,\, \kappa_{off} = \frac{k_{off}}{k_R^0} \,,\, \alpha = \frac{k_R^1}{k_R^0} \,,\, \beta = \frac{k_r^1}{k_r^0}. \tag{5}$$

Here, σ represents the synthesis rate constant of miRNA relative to that of the RNA, γ is the degradation rate constant of unbound miRNA relative to that of unbound RNA. α represents the degradation rate of RNA in complex relative to degradation in its unbound form. Analogously, β represents the degradation rate of miRNA in complex relative to the one in its unbound form. Since the nondimensional time is $t = k_R^0 \hat{t}$, it is inversely proportional to the RNA half-life $h = \ln(2)/k_R^0$. Thus, if we consider the median value of k_R^0 (see Supplementary Data section 5) one unit of nondimensional time t corresponds to approximately 1.44 RNA half-lives, as $t = 1$ corresponds to $\hat{t} = h/\ln(2) \approx 1.44\, h$.

1.1. Stability analysis

Building upon the framework established in [1], we examine model M1. While our nondimensionalization differs from that of Nordick et al., the core structure of the model and the steady-state analysis presented here closely follow their approach. According to the Deficiency One Theorem under the Chemical Reaction Network Theory [2], we recall that the species involved are r, R, C, and we calculate the number of complexes- reactants and products in this model, which is *n=5* $(R, r, C, r + R, \varnothing)$. According to the reactions in Eq. 1, the stoichiometric subspace for r, R, C is:

$$\mathbf{S} = span\left\{\begin{bmatrix}-1\\-1\\1\end{bmatrix}, \begin{bmatrix}1\\1\\-1\end{bmatrix}, \begin{bmatrix}1\\0\\0\end{bmatrix}, \begin{bmatrix}-1\\0\\0\end{bmatrix}, \begin{bmatrix}0\\1\\0\end{bmatrix}, \begin{bmatrix}0\\-1\\0\end{bmatrix}, \begin{bmatrix}1\\0\\-1\end{bmatrix}, \begin{bmatrix}0\\1\\-1\end{bmatrix}\right\} = span\left\{\begin{bmatrix}1\\1\\-1\end{bmatrix}, \begin{bmatrix}1\\0\\0\end{bmatrix}, \begin{bmatrix}0\\1\\0\end{bmatrix}\right\}$$

Thus, the rank of the stoichiometric subspace is $s = 3$. The number of linkage classes is $l = 1$ since all reactions and products sets are connected. Therefore, the network deficiency is $\delta = n - s - l = 1$. Then, according to the Deficiency One Theorem, the mass action system described by Eq. 2 or equivalently Eq. 4, has a single positive steady state. To assess the local stability of a steady state, we examine the locations of the eigenvalues of the Jacobian matrix in the complex plane. If all eigenvalues have negative real parts, the steady state is asymptotically stable. One approach to determine the location of the eigenvalues is the Routh-Hurwitz stability criterion [3]. The Jacobian matrix for M1 model is:

$$J = \begin{pmatrix} -1 - \kappa_{on} r & -\kappa_{on} R & \beta\gamma + \kappa_{off} \\ -\kappa_{on} r & -\gamma - \kappa_{on} R & \alpha + \kappa_{off} \\ \kappa_{on} r & \kappa_{on} R & -\alpha - \beta\gamma - \kappa_{off} \end{pmatrix} \tag{6}$$

The characteristic polynomial $p(\lambda) = det(J - \lambda) = \lambda^3 + a_2\lambda^2 + a_1\lambda + a_0$, where:

$$a_0 = \alpha\gamma + \beta\gamma^2 + r\alpha\gamma\kappa_{on} + R\beta\gamma\kappa_{on},$$

$$a_1 = \alpha + \gamma + \alpha\gamma + \beta\gamma + \beta\gamma^2 + \kappa_{off} + \gamma\kappa_{off} + R\kappa_{on} + r\alpha\kappa_{on} + r\gamma\kappa_{on} + R\beta\gamma\kappa_{on}$$

$$a_2 = 1 + \alpha + \gamma + \beta\gamma + \kappa_{off} + (r + R)\kappa_{on}$$

All roots of the polynomial have negative real parts if and only $a_2, a_0 > 0$ and $u = a_2a_1 - a_3a_0 > 0$. For any positive parameters, with steady state values for R and r, we have that a_0, a_2 are always positive. Then, we check that:

$$\begin{aligned} u = &(1 + \gamma)\left[\alpha^2 + (1 + \beta)\gamma(1 + \beta\gamma) + \alpha(1 + \gamma + 2\beta\gamma)\right] + (1 + \gamma)\kappa_{off}^2 + \Big[R(1 + 2\gamma(1 + \beta) + \beta(2 + \beta)\gamma^2 + \alpha(2 + \gamma + \gamma\beta)) \\ &+ r(\alpha^2 + \gamma(2 + \beta + \gamma + 2\beta\gamma) + \alpha(2 + (2 + \beta)\gamma))\Big]\kappa_{on} + (r + R)(R + \beta\gamma R + r(\alpha + \gamma))\kappa_{on}^2 \\ &+ \kappa_{off}\left[(1 + \gamma)(1 + 2\alpha + \gamma + 2\beta\gamma) + (r(1 + \alpha + 2\gamma) + R(2 + \gamma + \beta\gamma))\kappa_{on}\right] > 0 \end{aligned}$$

According to the Routh-Hurwitz stability criterion [3], we can conclude that for all positive rate constants, every eigenvalue of the system has a negative real part, ensuring that the unique positive steady state is asymptotically stable.

2. M2 model description and nondimensionalization

In this modified model we include a second RNA species able to bind the same miRNA species. We thus have one miRNA and two RNA species. Each RNA species forms a distinct molecular complex with the miRNA. The chemical reactions involved in this model are the following:

$$\varnothing \xrightarrow{S_{R_1}} \hat{R}_1 \quad \hat{R}_1 \xrightarrow{k^0_{R_1}} \varnothing \quad \varnothing \xrightarrow{S_{R_2}} \hat{R}_2 \quad \hat{R}_2 \xrightarrow{k^0_{R_2}} \varnothing \quad \varnothing \xrightarrow{S_r} \hat{r} \quad \hat{r} \xrightarrow{k^0_r} \varnothing \quad \hat{R}_1 + \hat{r} \xrightarrow{k_{on_1}} \hat{C}_1 \quad \hat{R}_2 + \hat{r} \xrightarrow{k_{on_2}} \hat{C}_2$$
$$\hat{C}_1 \xrightarrow{k_{off_1}} \hat{R}_1 + \hat{r} \quad \hat{C}_2 \xrightarrow{k_{off_2}} \hat{R}_2 + \hat{r} \quad \hat{C}_1 \xrightarrow{k^1_{r_1}} \hat{R}_1 \quad \hat{C}_2 \xrightarrow{k^1_{r_2}} \hat{R}_2 \quad \hat{C}_1 \xrightarrow{k^1_{R_1}} \hat{r} \quad \hat{C}_2 \xrightarrow{k^1_{R_2}} \hat{r} \tag{7}$$

where $\hat{R}_1$, $\hat{R}_2$, $\hat{r}$, $\hat{C}_1$ and $\hat{C}_2$ represent respectively the two RNA species, the miRNA and the two RNA-miRNA complexes.

The first two reactions represent the synthesis and degradation of the first RNA species, which occur respectively at rates S_{R_1} and $k^0_{R_1}$. Similarly, the next two reactions describe the synthesis and degradation of the second target, associated with rates S_{R_2} and $k^0_{R_2}$. The fifth and sixth reactions represent miRNA synthesis and degradation along with rates S_r and k^0_r. Next are the reactions of binding and unbinding for both RNA targets, occurring respectively with rates k_{on_1} and k_{off_1}, and k_{on_2} and k_{off_2}. Eventually, reactions of miRNA degradation in complex (i.e. recycling of one of the two targets) occur with rates $k^1_{r_1}$ and $k^1_{r_2}$ for the two complex species, whereas those of target degradation in complex (i.e. miRNA recycling) follow respectively rates $k^1_{R_1}$ and $k^1_{R_2}$.

Considering the law of mass action, we describe the dynamics of the five molecular species using the following ordinary differential equations (ODEs):

$$\begin{cases} \frac{d\hat{R}_1}{d\hat{t}} = S_{R_1} - k_{on_1}\hat{R}_1\hat{r} + k_{off_1}\hat{C}_1 - k^0_{R_1}\hat{R}_1 + k^1_{r_1}\hat{C}_1 \\ \frac{d\hat{R}_2}{d\hat{t}} = S_{R_2} - k_{on_2}\hat{R}_2\hat{r} + k_{off_2}\hat{C}_2 - k^0_{R_2}\hat{R}_2 + k^1_{r_2}\hat{C}_2 \\ \frac{d\hat{r}}{d\hat{t}} = S_r - k_{on_1}\hat{R}_1\hat{r} - k_{on_2}\hat{R}_2\hat{r} + k_{off_1}\hat{C}_1 + k_{off_2}\hat{C}_2 - k^0_r\hat{r} + k^1_{R_1}\hat{C}_1 + k^1_{R_2}\hat{C}_2 \\ \frac{d\hat{C}_1}{d\hat{t}} = k_{on_1}\hat{R}_1\hat{r} - k_{off_1}\hat{C}_1 - k^1_{R_1}\hat{C} - k^1_{r_1}\hat{C}_1 \\ \frac{d\hat{C}_2}{d\hat{t}} = k_{on_2}\hat{R}_2\hat{r} - k_{off_2}\hat{C}_2 - k^1_{R_2}\hat{C} - k^1_{r_2}\hat{C}_2 \end{cases} \tag{8}$$

where $\hat{R}_1$ and $\hat{R}_2$ represent concentrations of the two unbound RNA species, $\hat{r}$ describes miRNA concentration, whereas $\hat{C}_1$ and $\hat{C}_2$ describe concentrations of the two RNA-miRNA complex species.

Adopting the same approach as for model M1, we make the following changes of variables using the synthesis and degradation rates of the first RNA, S_{R_1} and $k^0_{R_1}$:

$$\hat{t} = \frac{t}{k^0_R}, \quad \hat{R}_1 = \frac{R_1 S_{R_1}}{k^0_{R_1}}, \quad \hat{R}_2 = \frac{R_2 S_{R_1}}{k^0_{R_1}}, \quad \hat{r} = \frac{r S_{R_1}}{k^0_{R_1}}, \quad \hat{C}_1 = \frac{C_1 S_{R_1}}{k^0_{R_1}}, \quad \hat{C}_2 = \frac{C_2 s_{R_1}}{k^0_{R_1}} \tag{9}$$

which yield the nondimensional ODE system:

$$\begin{cases} \frac{dR_1}{dt} = 1 - \kappa_{on_1}R_1 r + \kappa_{off_1}C_1 - R_1 + \gamma\beta_1 C_1 \\ \frac{dR_2}{dt} = \delta R_2 - \kappa_{on_2}R_2 r + \kappa_{off_2}C_2 - \epsilon R_2 + \gamma\beta_2 C_2 \\ \frac{dr}{dt} = \sigma - \kappa_{on_1}R_1 r + \kappa_{off_1}C_1 - \gamma r + \alpha_1 C_1 - \kappa_{on_2}R_2 r + \kappa_{off_2}C_2 + \alpha_2 C_2 \\ \frac{dC_1}{dt} = \kappa_{on_1}R_1 r - \kappa_{off_1}C_1 - \gamma\beta_1 C_1 - \alpha_1 C_1 \\ \frac{dC_2}{dt} = \kappa_{on_2}R_2 r - \kappa_{off_2}C_2 - \gamma\beta_2 C_2 - \alpha_2 C_2 \end{cases} \tag{10}$$

where:

$$\sigma = \frac{S_r}{S_{R_1}}, \kappa_{on_i} = \frac{k_{on_i}S_{R_1}}{k^0_{R_1}}, \kappa_{off_i} = \frac{k_{off_i}}{k^0_{R_1}}, \alpha_i = \frac{k^1_{R_i}}{k^0_{R_1}}, \beta_i = \frac{k^1_{r_i}}{k^0_r}, \gamma = \frac{k^0_r}{k^0_{R_1}}, \delta = \frac{S_{R_2}}{S_{R_1}}, \epsilon = \frac{k^0_{R_2}}{k^0_{R_1}} \tag{11}$$

with $i = 1, 2$ referring to one of the two target RNA species.

Here, σ represents the synthesis rate constant of miRNA relative to that of the first RNA target. κ_{on_i} and κ_{off_i} represent miRNA binding and unbinding rates of the i-th RNA species. α_i describes the degradation rate of the i-th RNA in complex relative to that of its unbound form. β_i is the degradation rate of miRNA in the i-th complex relative to its unbound form. γ is the degradation rate of unbound miRNA relative to that of the first RNA species. δ and ϵ describe respectively the synthesis and the degradation rate of the second target with respect to the first one.

3. Nondimensionalization of periodic miRNA synthesis rate

Working in the dimensional framework of model M1 (Eq. 2), we can introduce periodic miRNA expression into the system by representing its synthesis rate S_r as a square wave, which we denote as $S_{r_{pulse}}$:

$$S_{r_{pulse}}(\hat{t}) = \begin{cases} S_r & \text{if } n\hat{T} < \hat{t} \leq (n+d)\hat{T}, \quad n = 0,1,2,\dots \\ 0 & \text{if } (n+d)\hat{T} < \hat{t} \leq (n+1)\hat{T}, \quad n = 0,1,2,\dots \end{cases} \tag{12}$$

where S_r is now the amplitude of the square wave, $\hat{T}$ represents its period and d represents its duty cycle ($0 < d < 1$). Conversely, the RNA synthesis rate is kept constant:

$$S_R(\hat{t}) = S_R \tag{13}$$

and thus, following the nondimensionalization approach illustrated in section 1 (see Eqs. 3), the nondimensional periodic rate of miRNA synthesis is given by the ratio between $S_{r_{pulse}}(\hat{t})$ and the RNA synthesis rate $S_R(\hat{t})$, which results as:

$$\sigma_{pulse}(t) = \frac{S_{r_{pulse}}(\hat{t})}{S_R} = \begin{cases} \frac{S_r}{S_R} = \sigma & \text{if } nT < t \leq (n+d)T, \quad n = 0,1,2,\dots \\ 0 & \text{if } (n+d)T < t \leq (n+1)T, \quad n = 0,1,2,\dots \end{cases} \tag{14}$$

where σ is the nondimensional pulse amplitude and T is its nondimensional period.
Since time is scaled due to nondimensionalization (see Eqs 3), the pulse frequency $f = 1/T$ is also scaled: one scaled time unit ($t = 1$) corresponds to $k_R^0 \hat{t} = \frac{ln(2)}{h}\hat{t} = 1$ or $\hat{t} = h/\ln(2)$, where $\hat{t}$ is the dimensional time and h is the half-life of the target. Considering the median value of k_R^0 (see section 5), one unit of nondimensional time corresponds to approximately 1.44 target RNA half-lives. Thus, a nondimensional frequency $f = 1$ corresponds to pulses occurring every $\approx 1,44$ RNA half-lives.
Identical reasonings hold for model M2 if we replace R by the first target, R_1. Since both targets R_1 and R_2 have constant synthesis rates:

$$S_{R_1}(\hat{t}) = S_{R_1} \tag{15}$$

$$S_{R_2}(\hat{t}) = S_{R_2} \tag{16}$$

the nondimensional periodic rate of miRNA synthesis will result as:

$$\sigma_{pulse}(t) = \frac{S_{r_{pulse}}(\hat{t})}{S_{R_1}(\hat{t})} = \begin{cases} \frac{S_r}{S_{R_1}} = \sigma & \text{if } nT < t \leq (n+d)T, \quad n = 0,1,2,\dots \\ 0 & \text{if } (n+d)T < t \leq (n+1)T, \quad n = 0,1,2,\dots \end{cases} \tag{17}$$

4. Conservation of relative miRNA-to-RNA dose

In our dimensional version of model M1 (see Eqs. 2), the amounts of miRNA and target RNA produced in a period $\hat{T}$ are obtained by integrating in the time interval $[0, \hat{T}]$ respectively the square wave rate given by Eq. (12) and the constant rate given by Eq. (13). Thus, the relative amount of miRNA and target RNA produced in a period will be the ratio of such two integrals:

$$\frac{\int_0^{\hat{T}} S_{r_{pulse}}(t)\,dt}{\int_0^{\hat{T}} S_R(t)\,dt} = \frac{S_r \cdot \hat{T} \cdot d}{S_R \cdot \hat{T}} = \sigma \cdot d \tag{18}$$

where σ is the nondimensional amplitude of periodic miRNA synthesis rate and d is its duty cycle. The quantity $\sigma \cdot d$ - which we can refer to as "relative miRNA-RNA$_1$ dose" - results independent from the period size and thus from the input's frequency. It is thus sufficient to keep the duty cycle d and the amplitude σ fixed to guarantee that the relative dose of miRNA and RNA is maintained constant for any frequency, as long as we consider an integer number of pulses.
In the case of model M2, which involves two different target RNAs, the same equation as (18) holds by replacing the rate S_R with that of the first target RNA, S_{R_1}: the relative dose of periodically produced miRNA with respect to $\hat{R}_1$ will be $\sigma \cdot d$. With a similar approach we can derive the miRNA dose produced with respect to the second target, $\hat{R}_2$. Considering that the dimensional amounts of miRNA and target $\hat{R}_2$ produced in a period are obtained by integrating in the time interval $[0, \hat{T}]$ rate constants given respectively by equations (12) and (16), their ratio would result as:

$$\frac{\int_0^{\hat{T}} S_{r_{pulse}}(t)\,dt}{\int_0^{\hat{T}} S_{R_2}(t)\,dt} = \frac{S_r \cdot \hat{T} \cdot d}{S_{R_2} \cdot \hat{T}} = \frac{\sigma}{\delta} \cdot d \tag{19}$$

where σ and δ are nondimensional parameters describing respectively the amplitude of periodic miRNA synthesis rate and the synthesis rate of target R_2 relative to the first, R_1. Thus, model M2 requires conserving both quantities $\sigma \cdot d$ and $\frac{\sigma}{\delta} \cdot d$ - which we refer to as respectively "relative miRNA-RNA$_1$ dose" and "relative miRNA-RNA$_2$ dose" - in order to maintain constant the amount of regulator synthesized relative to each target in a complete pulse. Therefore, since we assume a fixed duty cycle value of 0.5, we fix parameters σ and δ while varying the frequency of pulses.

To model also a constant rate of miRNA synthesis $S_{r_{const}}$ that conserves the miRNA-to-RNA dose produced in a period, we impose using the dimensional model M1 (Eqs. 2, that the miRNA amount produced with such constant synthesis rate with respect to the target RNA equals $\sigma \cdot d$:

$$\frac{\int_0^T S_{r_{const}}(t)\,dt}{\int_0^T S_R(t)\,dt} = \sigma \cdot d \tag{20}$$

which yields, considering that $S_{r_{const}}(t)$ and $S_R(t)$ are both constant:

$$\frac{S_{r_{const}} \cdot T}{S_R \cdot T} = \sigma \cdot d \tag{21}$$

where the left-hand side represents our nondimensional constant miRNA synthesis rate σ_{const}:

$$\sigma_{const} = \sigma \cdot d \tag{22}$$

Thus, it is sufficient to multiply the amplitude σ by the duty cycle d to obtain the constant miRNA synthesis rate value with dose equivalent to the periodic one.

If we now consider model M2, identical reasonings (Eqs. (20), (21), (22)) can be applied by replacing R with R_1: a constant miRNA synthesis rate $\sigma \cdot d$ guarantees the conservation of the miRNA dose relative to R_1.

To ensure conservation of the miRNA dose with respect to the second target, with a similar approach we impose:

$$\frac{\int_0^T S_{r_{const}}(t)\,dt}{\int_0^T S_{R_2}(t)\,dt} = \frac{\sigma}{\delta} \cdot d \tag{23}$$

which yields, considering that both $S_{r_{const}}$ and S_{R_2} are constant:

$$\frac{S_{r_{const}} \cdot T}{S_{R_2} \cdot T} = \frac{\sigma}{\delta} \cdot d \tag{24}$$

and thus, if we simultaneously multiply and divide the left-hand side by S_{R_1}:

$$\frac{S_{r_{const}} \cdot T}{S_{R_2} \cdot T} \cdot \frac{S_{R_1}}{S_{R_1}} = \frac{\sigma}{\delta} \cdot d \tag{25}$$

recalling that $\delta = \frac{S_{R_2}}{S_{R_1}}$ (Eqs. 11), we confirm that the nondimensional constant miRNA synthesis rate

$$\sigma_{const}(t) = \sigma \cdot d \tag{26}$$

guarantees relative miRNA-to-RNA dose conservation for both targets also in model M2.

5. Estimation of biological rate constants in model M1

In this section, we determine biologically realistic ranges of parameters for model M1. Following the general approach in [1], we begin by estimating dimensional rate constants to subsequently derive corresponding ranges for the nondimensional parameters under our chosen scaling, which differs slightly from that used by Nordick et al.

The median mammalian mRNA half-life in absence of post-transcriptional regulation, which corresponds to k_R^0 in our model, is estimated to be 4 hours [4]. The average diameter of a mammalian cell is 13 μm [5], and thus the corresponding cell volume - considering the cell as a sphere - is $1.15 \times 10^{-10} L$. Since the number of mRNA molecules per gene in a single cell can range from a few copies to tens of thousands of copies, we adopt 200 molecules as mean value, in agreement with [6] and [7]. We can therefore calculate the mean RNA molar

concentration as $\bar{R} = n_R/N_A/V = 2.9{\times}10^{-10}\ M$, and estimate the RNA transcription rate as $S_R = k_R^0\bar{R} = 1.4{\times}10^{-14}\ Ms^{-1}$.
MiRNA half-lives are observed to be approximately four times the mRNA half-lives [8]. Therefore, we estimate the median of the scaled degradation rate $\gamma = \frac{k_r^0}{k_R^0}$ to be 1/4. However, as miRNA half lives can vary from about 4 hr up to 48 hr [9], it is possible that some miRNAs may have shorter half lives than their target RNAs, and we thus sample γ log-uniformly in the range $[10^{-1}/4 - 10/4]$ as suggested by [1].
For the estimation of the miRNA-RNA association constant k_{on} we adopt ranges spanning the orders of magnitude reported in [10]: $[10^6 - 10^9]\ M^{-1}s^{-1}$. Since the dissociation constant $K = k_{off}/k_{on}$ was estimated to be $3.7pM$ [11], we derive a suitable range for the miRNA-RNA dissociation rate constant as k_{off} as $[3.7 \times 10^{-6}, 3.7 \times 10^{-3}]s^{-1}$. Then using the estimated values of s_R and k_R^0 for scaling, we obtain ranges for nondimensional association and dissociation parameters: $\kappa_{on} = [6, 6 \times 10^3]$ and $\kappa_{off} = [7.7 \times 10^{-2}, 7.7 \times 10^1]$. In this way, using ranges for nondimensional parameters, we also explore scenarios where the number of RNA molecules per cell spans smaller and greater orders of magnitude with respect to the estimated mean value of 200 molecules.
Parameters α and β were both sampled in the interval [1/8, 16], estimated based on previous experimental data reported in [12; 13].
Eventually, to explore scenarios where miRNA synthesis is either faster, comparable or slower than synthesis of its target, we adopted σ values ranging from 10^-1 to 10^1. Tables S1 and S2 report respectively estimated values of dimensional and nondimensional model parameters.

Parameter	Biological meaning	Estimated median values / ranges
k_R^0	RNA degradation rate	$4.8 \times 10^{-5}s^{-1}$
S_R	RNA transcription rate	$1.4 \times 10^{-14}Ms^{-1}$
K	Dissociation constant	3.7 pM
k_{on}	Binding rate	$[10^6 - 10^9]\ M^{-1}s^{-1}$
k_{off}	Unbinding rate	$[3.7 \times 10^{-6} - 3.7 \times 10^{-3}]\ s^{-1}$

Table S1: Dimensional M1 model's parameter ranges.

Nondimensional parameter	Biological meaning	Range
σ	Scaled miRNA transcription rate	$[10^{-1}, 10^1]$
κ_{on}	Scaled binding rate	$[6, 6 \times 10^3]$
κ_{off}	Scaled unbinding rate	$[7.7 \times 10^{-2}, 7.7 \times 10^1]$
α	Bound relative to unbound RNA degradation rate	[1/8, 16]
β	Bound relative to unbound miRNA degradation rate	[1/8, 16]
γ	Scaled miRNA degradation rate	$[10^{-1}/4, 10/4]$

Table S2: Nondimensional M1 model's parameter ranges.

6. RNA equilibration time in model M1

In this section we analyze the equilibration time τ in model M1, i.e. the time it takes the RNA species to reach steady state in the presence of constant miRNA synthesis (σ_{const}). τ results from the interaction of the multiple timescales involved in the regulatory motif, and thus depends on all model parameters. To understand which reaction rate constants most greatly determine the value of τ, we randomly sampled the parameter space of model M1 using Latin Hypercube Sampling [14] and we computed Partial rank correlation coefficients between τ and each parameter. These correlation results - summarized in Table S3 - show that the miRNA degradation rate relative to the target (γ) and the miRNA degradation rate in complex with the target (β) are the parameters most highly correlated with τ. In particular, τ is strongly anticorrelated with γ, showing that the RNA equilibration time is mostly dictated by its stability relative to the miRNA.

σ	γ	κ_{on}	κ_{off}	α	β
0.0807	-0.8926	0.0026	-0.0037	-0.0132	-0.2112

Table S3: Partial rank correlation coefficients between nondimensional M1 model parameters and τ, i.e. the RNA equilibration time under constant miRNA synthesis σ_{const}.

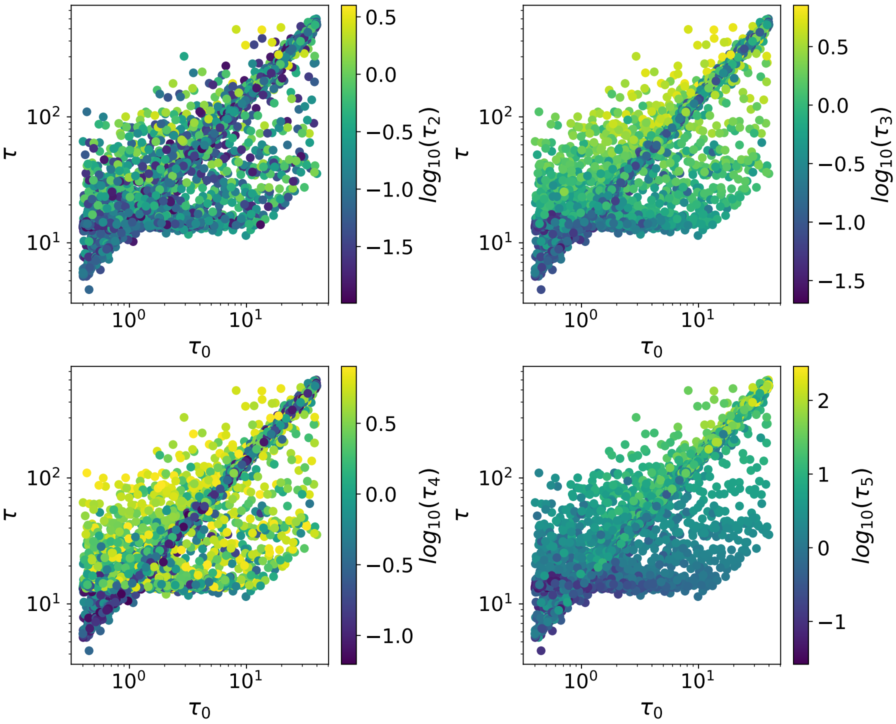

Figure S1: Time τ as a function of the relative RNA-miRNA degradation rate constant ($\tau_0 = \gamma^{-1}$). The different dot colors indicate the log-scale magnitude of times τ_2 (top left plot), τ_3 (top right plot), τ_4 (bottom left plot), τ_5 (bottom right plot).

In addition to relating τ to single rate constants, we can also examine its relationship with significant timescales that arise from a combination of parameters. In particular, we might consider timescales related to the lifetimes of molecular species and those linked to complex processing. For instance, the value $\tau_0 = \gamma^{-1}$ corresponds to the RNA decay rate relative to the miRNA, while the RNA half life alone is related to one unit of time (see model M1 nondimensionalization in section 1). The following times are related to complex processing: $\tau_2 = (\kappa_{off} + \alpha + \gamma\beta)^{-1}$ is the average lifetime of the complex C before it either unbinds or is degraded; $\tau_3 = (\alpha + \gamma\beta)^{-1}$ represents the average time needed for the complex to be degraded and recycled into either the miRNA or the RNA in absence of unbinding; $\tau_4 = \alpha^{-1}$ and $\tau_5 = \gamma\beta^{-1}$ are the times needed for the complex to be degraded and to be recycled respectively into miRNA and RNA, in absence of all other processes. Note that $\tau_2 < \tau_3 < \tau_4$ and $\tau_3 < \tau_5$.
To relate τ with such timescales, we randomly sampled the model M1 parameters in their estimated range (see section 5), and for each sample we computed both τ and the times τ_0, τ_2, τ_3, τ_4, τ_5. In Figure S1 we show τ as a function of τ_0, i.e. the timescale of RNA degradation relative to the miRNA, and we describe the contribution of the remaining timescales using color scales. The distribution of sampled (τ_0, τ) data points is consistent with the positive correlation found between τ and γ (Table S3). Timescales τ_2, τ_3, τ_4, τ_5 appear all positively related with τ, in line with the fact that a slower kinetics for any reaction implies an overall longer equilibration time. However, as previously highlighted, RNA half-life is the timescale that mostly affects the overall equilibration time τ: unless complex processing timescales are short, τ is tightly related to τ_0 and therefore tends to align along the linear axis where correlation is maximal.

7. Alternative models: loss and retention of frequency preference

In this section, we outline analytical and numerical approaches used to identify mathematical features required for the emergence of frequency preference in a miRNA-target interaction model.

7.1. MiRNA-mediated regulation modelled as effective target degradation lacks frequency preference behaviors

In this section we consider the simplest model for miRNA-mediated regulation of a target RNA, inspired by the framework studied in [15]. This model adopts an effective RNA degradation rate to model miRNA-induced repression, thus the effect of miRNA is simply an increased degradation of the target. To mimic constant and pulsatile miRNA synthesis cases, we model such effective degradation as either a constant rate or a periodic one given by a train of square pulses. Due to these assumptions, the model is one-dimensional and linear; thus, an analytical

solution is possible.
We start by considering the constitutive expression for the RNA species. Following the same nondimensionalization choice outlined in section 1, the dimensionless model for unregulated RNA is represented by the following equation:

$$\frac{dR_{basal}}{dt} = 1 - R_{basal} \tag{27}$$

Considering that the initial concentration is zero, the solution for the unrepressed RNA concentration as a function of time is:

$$R_{basal}(t) = 1 - e^{-t} \tag{28}$$

Next, we introduce the effective degradation rate Γ to model miRNA-induced repression. In this case the following equation holds for RNA concentration:

$$\frac{dR}{dt} = S_R - \Gamma R \tag{29}$$

where S_R is the mRNA synthesis rate in this effective model. Let us consider the case of constant degradation, whose rate we denote as Γ_c. Assuming a null initial RNA concentration, its temporal solution is:

$$R_c(t) = \frac{S_R}{\Gamma_c}\left(1 - e^{-\Gamma_c t}\right) \tag{30}$$

Next, we consider the pulsatile degradation case, modelling the effective rate Γ as a square wave:

$$\Gamma_p(t) = \begin{cases} \Gamma_1 & \text{if } t \in [nT, (n+d)T) \\ \Gamma_2 & \text{if } t \in [(n+d)T, (n+1)T) \end{cases} \tag{31}$$

where Γ_1 and Γ_2 are constants, T represents the period, and $0 \leq d \leq 1$ is the duty cycle.
In this case, the temporal RNA concentration R_p follows this expression:

$$R_p(t) = \begin{cases} \frac{S_R}{\Gamma_1} + \left[R_p(nT) - \frac{S_R}{\Gamma_1}\right] e^{-\Gamma_1(t-nT)} & \text{for } t \in [nT, (n+d)T) \\ \frac{S_R}{\Gamma_2} + \left[R_p((n+d)T) - \frac{S_R}{\Gamma_2}\right] e^{-\Gamma_2(t-(n+d)T)} & \text{for } t \in [(n+d)T, (n+1)T) \end{cases} \tag{32}$$

Now, assuming that the initial RNA concentration is zero, for the first period (*n=0*) the solution is:

$$R_p(t) = \begin{cases} \frac{S_R}{\Gamma_1}\left(1 - e^{-\Gamma_1 t}\right) & \text{if } t \in [0, dT) \\ \frac{S_R}{\Gamma_2} + \left(-\frac{S_R}{\Gamma_1} e^{-\Gamma_1 dT} + \frac{(\Gamma_2-\Gamma_1)S_R}{\Gamma_1\Gamma_2}\right) e^{-\Gamma_2(t-dT)} & \text{if } t \in [dT, T) \end{cases} \tag{33}$$

The fold repression in the first period of the square wave is calculated following the definition given in the Materials and Methods section, as:

$$FR_p = \frac{\frac{1}{T}\int_0^T R_{basal}(t)dt}{\frac{1}{T}\int_0^T R_p(t)dt} \tag{34}$$

Where the numerator results as:

$$\frac{1}{T}\int_0^T R_{basal}(t)dt = \int_0^T 1 - e^{-t}dt = \frac{T + e^{-T} - 1}{T} \tag{35}$$

And the denominator is:

$$\frac{1}{T}\int_0^T R_p(t)dt = \frac{1}{T}\left\{\frac{S_R}{\Gamma_1^2}\left(-1 + d\Gamma_1 T + e^{-d\Gamma_1 T}\right) + \frac{S_R}{\Gamma_1\Gamma_2^2} e^{-(d\Gamma_1+\Gamma_2)T}\left[(\Gamma_1 - \Gamma_2)e^{d(\Gamma_1+\Gamma_2)T} - \Gamma_2 e^{\Gamma_2 T} + \Gamma_2 e^{d\Gamma_2 T} \right.\right.$$
$$\left.\left. + e^{(d\Gamma_1+\Gamma_2)T}(\Gamma_2 + \Gamma_1(-1 + \Gamma_2 T - d\Gamma_2 T))\right]\right\} \tag{36}$$

Thus, fold repression over the first period reads:

$$FR_p = \left(T + e^{-T} - 1\right)\left(\frac{S_R}{\Gamma_1^2}\left(-1 + d\Gamma_1 T + e^{-d\Gamma_1 T}\right)\right.$$
$$\left. + \frac{S_R}{\Gamma_1\Gamma_2^2} e^{-(d\Gamma_1+\Gamma_2)T}\left[(\Gamma_1 - \Gamma_2)e^{d(\Gamma_1+\Gamma_2)T} - \Gamma_2 e^{\Gamma_2 T} + \Gamma_2 e^{d\Gamma_2 T} + e^{(d\Gamma_1+\Gamma_2)T}(\Gamma_2 + \Gamma_1(-1 + \Gamma_2 T - d\Gamma_2 T))\right]\right)^{-1} \tag{37}$$

Next, we compare the fold repression achieved over the same time span $[0, T]$ for an equivalent constant effective degradation rate Γ_c, ensuring that the mean amount of RNA degraded over this period matches that of the pulsatile model:

$$\Gamma_c = \frac{1}{T}\int_0^T \Gamma(t)dt = \Gamma_1 d + \Gamma_2(1-d) \tag{38}$$

In this case, also considering that the initial concentration is zero, the RNA concentration R_c as a function of time is:

$$R_c(t) = \frac{S_R}{\Gamma_c}\left(1 - e^{-\Gamma_c t}\right) \tag{39}$$

Thus, the temporal average of RNA over the considered time span is:

$$\frac{1}{T}\int_0^T R_c(t)dt = \frac{S_R}{\Gamma_c T}\left[T + \frac{1}{\Gamma_c}\left(e^{-\Gamma_c T} - 1\right)\right] \tag{40}$$

Therefore, fold repression for a constant effective degradation over the time window $[0, T]$ results:

$$\begin{aligned} FR_c &= \frac{T + e^{-T} - 1}{\frac{S_R T}{\Gamma_c} - \frac{S_R}{\Gamma_c^2}\left(e^{-\Gamma_c T} - 1\right)} \\ &= \frac{T + e^{-T} - 1}{\frac{S_R T}{\Gamma_1 d + \Gamma_2(1-d)}\left(T - \frac{1}{(\Gamma_1 d + \Gamma_2(1-d))^2}\left(e^{-\Gamma_1 dT - \Gamma_2(1-d)T} - 1\right)\right)} \end{aligned} \tag{41}$$

Then, we compare fold repression outputs generated by the two modes of miRNA-mediated degradation - constant and periodic - by taking their ratio. For the sake of simplicity we consider $d = 1/2$ as in our main model M1, thus $\Gamma_c = \frac{\Gamma_1+\Gamma_2}{2}$. Then, the ratio results as:

$$\begin{aligned} \frac{FR_c}{FR_p} &= \frac{\int_0^T R_p(t)dt}{\int_0^T R_c(t)dt} \\ &= \frac{2S_R e^{-(\Gamma_1+\Gamma_2)T/2}\left[2 + e^{(\Gamma_1+\Gamma_2)T/2}(\Gamma_1 T + \Gamma_2 T - 2)\right]}{4S_R(\Gamma_1+\Gamma_2)^2\left[\frac{e^{-(\Gamma_1+\Gamma_2)T/2} - 1}{(\Gamma_1+\Gamma_2)^2} + \frac{T}{2(\Gamma_1+\Gamma_2)}\right]} \\ &= \frac{e^{-(\Gamma_1+\Gamma_2)T/2} - 1 + \frac{(\Gamma_1+\Gamma_2)T}{2}}{e^{-(\Gamma_1+\Gamma_2)T/2} - 1 + \frac{(\Gamma_1+\Gamma_2)T}{2}} \\ &= 1 \end{aligned} \tag{42}$$

Thus, over the first period $[0, T]$, the fold repression given by a periodic effective degradation is equivalent to the one given by a constant effective degradation, meaning that this simplified model exhibits no advantage A.
To determine whether the periodic case exhibits frequency preference, we analyze whether FR_p presents any extrema for some frequency value. Thus, let us consider the fold repression FR_p as a function of the input's period T, and differentiate it with respect to T. If any extrema exist, $\frac{dFR_p}{dT}$ must be equal to zero for some value of T.

$$\begin{aligned} \frac{dFR_p}{dT} = &-((e^{2d\Gamma_1 T}\Gamma_1^2\Gamma_2^2\{S_R\Gamma_1\Gamma_2 e^{-(1+d\Gamma 1+\Gamma_2)T}(1 + e^{d\Gamma_1 T})[\Gamma_1 e^{(d\Gamma_1+\Gamma_2)T} - \Gamma_2 e^{d\Gamma_2 T} + \\ &(\Gamma_2 - \Gamma_1)e^{d(\Gamma_1+\Gamma_2)T} + d(-1 + e^{d\Gamma_1 T})(e^{\Gamma_2 T} - e^{d\Gamma_2 T})(\Gamma_2 - \Gamma_1)] + \\ &S_R e^{-(1+d\Gamma_1)T}(e^T - 1)(-\Gamma_1\Gamma_2 e^{(d-1)\Gamma_2 T} + \Gamma_2(\Gamma_1 - \Gamma_2) + \Gamma_1(\Gamma_2 - \Gamma_1)e^{(-\Gamma_2+d(\Gamma_1+\Gamma_2))T} + \\ &e^{d\Gamma_1 T}(\Gamma_2^2 + \Gamma_1^2(1 + (d-1)\Gamma_2 T) - \Gamma_1\Gamma_2(1 + d\Gamma_2 T))\}))/(S_R^2(\Gamma_1\Gamma_2(-e^{(d-1)\Gamma_1 T} + \\ &\Gamma_2(\Gamma_1 - \Gamma_2) + \Gamma_1(\Gamma_2 - \Gamma_1)e^{(-\Gamma_2+d(\Gamma_1+\Gamma_1)T} + e^{d\Gamma_1 T}(\Gamma_2^2 + \Gamma_1^2(1 + (d-1)\Gamma_2 T) - \\ &\Gamma_1\Gamma_2(1 + d\Gamma_2 T)))^2)) \end{aligned} \tag{43}$$

Considering that all parameters S_R, d, Γ_1 and Γ_2 are positive, we conclude that the derivative $\frac{dFR_p}{dT}$ is never equal to zero for any value of T. Thus, FR_p is monotone with respect to frequency, indicating that there is no frequency preference in the first input period in this simplified model.
We next consider a later stage of the periodic input. Let's thus study the solution over the n-th period, with $n \to \infty$. Considering that the degradation rate is T-periodic, if R is the solution of eq. 29, then *u(t) = R(t+T)-R(t)* satisfies:

$$\frac{du}{dt} = -\Gamma_c(t)u \tag{44}$$

as:

$$\frac{dR(t+T)}{dt} = S_R - \Gamma_c(t+T)R(t+T) = S_R - \Gamma_c(t)R(t+T) \tag{45}$$

and therefore the solution is given by:

$$u(t) = ke^{\int_0^t -\Gamma_c(v)dv} \tag{46}$$

Since Γ_c is bounded, we have that $u(t) = R(t+T) - R(t) \to 0$ as $t \to \infty$. This implies that the solution becomes asymptotically periodic, enabling us to arrive to an solution in the *n-th* period, for n large enough.
In this limit, we expect that $R(nT) \to R((n+1)T)$, then:

$$R(nT) = \frac{S_R}{\Gamma_1} + \left(R(nT) - \frac{S_R}{\Gamma_1}\right)e^{-\Gamma_1(nT-nT)} = R((n+1)T) = \frac{S_R}{\Gamma_2} + \left(R((n+d)T) - \frac{S_R}{\Gamma_2}\right)e^{-\Gamma_2((n+1)T-(n+d)T))} \tag{47}$$

From where we get:

$$R((n+d)T) = \frac{S_R + (\Gamma_2 R(nT) - S_R)e^{\Gamma_2(1-d)T}}{\Gamma_2} \tag{48}$$

which is valid for $n \to \infty$. We recall that the exact expression for $R((n+d)T)$ is given by:

$$R((n+d)T) = \frac{S_R}{\Gamma_1} + \left(R(nT) - \frac{S_R}{\Gamma_1}\right)e^{-\Gamma_1 dT} \tag{49}$$

Then, from eqs. 48 and 49 we derive an expression for $R(nT)$ in the asymptotically periodic limit:

$$R(nT) = \frac{S_R\left[\Gamma_1\left(1 - e^{\Gamma_2(1-d)T}\right) - \Gamma_2\left(1 - e^{-\Gamma_1 dT}\right)\right]}{\Gamma_1\Gamma_2\left(e^{-\Gamma_1 dT} - e^{\Gamma_1(1-d)T}\right)} \tag{50}$$

Note that this expression is independent of n. We can obtain an expression for $R((n+d)T)$ replacing eq. 50 in eq. 49. Under these assumptions and considering $d = 1/2$, we can calculate the integral over the n-th period:

$$\int_{NT}^{(N+1)T} R_{pulse}(t)dt = \frac{S_R}{\Gamma_1}\left[\frac{(-1+e^{\Gamma_1 T/2})(-1+e^{\Gamma_2 T/2})(\Gamma_1-\Gamma_2)}{(-1+e^{(\Gamma_1+\Gamma_2)T/2})\Gamma_1\Gamma_2} + \frac{T}{2}\right] + \\ \frac{S_R}{\Gamma_1\Gamma_2^2}\frac{2(-1+e^{\Gamma_1 T/2})(-1+e^{\Gamma_2 T/2})\Gamma_2 + \Gamma_1\left[-2+2e^{\Gamma_1 T/2}+2e^{\Gamma_2 T/2} - \Gamma_2 T + e^{(\Gamma_1+\Gamma_2)T/2}(-2+\Gamma_2 T)\right]}{2(-1+e^{(\Gamma_1+\Gamma_2)T/2})} \tag{51}$$

The integral for the basal expression is:

$$\int_{nT}^{(n+1)T} = R_{basal}(t)dt = T + e^{-(n+1)T} - e^{-nT} \tag{52}$$

Then, we can compute fold repression FR_{pulse} over the n-th period - for n large enough - by taking the ratio of eqs 51 and 52. We can compute the derivative of FR_{pulse} with respect to T, yet no general conclusions can be derive about its monotonicity without further parameter assumptions.

In conclusion, the effective degradation model fails to capture the dynamic features - present in the full model (M1) - which confer an advantage to periodic over constant miRNA and enable frequency preference. Unlike the original model, the simplified model does not incorporate explicit binding and unbinding processes, resulting in the absence of nonlinearities within the system. As highlighted in Table 1 of the main text, explicit complex formation is crucial, with the binding constant κ_{on} plays a critical role in determining selectivity (S) and advantage (A), the core metrics that characterize these features.

7.2. Simpler models display frequency preference and advantage but lack closed-form solutions

In this section we introduce a simplified model of miRNA-mediated regulation that, while less complex than the one discussed in the main text (model M1), still incorporates nonlinearities. This model assumes that the complex is processed rapidly enough to assume that its concentration always equals its steady state value.
Starting from eq. 4, we assume fast complex processing, leading to the steady state expression:

$$\frac{dC}{dt} = 0 \longrightarrow C_{ss} = \frac{\kappa_{on} rR}{\kappa_{off} + \alpha + \gamma\beta} \tag{53}$$

Thanks to the fast complex processing assumption, we can replace eq. 53 in eq. 4, yielding the following equations for RNA and miRNA dynamics in such regime:

$$\begin{cases} \frac{dr}{dt} = \sigma - \gamma r - \kappa_1 r R \\ \frac{dR}{dt} = 1 - R - \kappa_2 r R \end{cases} \tag{54}$$

where:

$$\kappa_1 = \frac{\gamma\beta\kappa_{on}}{\kappa_{off} + \alpha + \gamma\beta}, \ \kappa_2 = \frac{\alpha\kappa_{on}}{\kappa_{off} + \alpha + \gamma\beta} \tag{55}$$

To explore the possibility of an analytical solution, we further assume that the miRNA is not degraded within the complex (and thus the RNA is not recycled) by posing $\beta = 0$ which implies $\kappa_1 = 0$ in the first equation. This results in a simplified model that accounts only for synthesis and degradation of both miRNA and RNA, with an RNA degradation term that is proportional to the concentrations of both species. Despite the absence of an explicit equation for the complex, this term preserves the nonlinearities introduced by complex formation.
The model is described by the following equations:

$$\begin{cases} \frac{dr}{dt} = \sigma - \gamma r \\ \frac{dR}{dt} = 1 - R - \kappa R r \end{cases} \tag{56}$$

where $\kappa = \frac{\alpha\kappa_{on}}{\kappa_{off}+\alpha}$.
As in previous cases, we model both constant and periodic miRNA synthesis. We begin by considering a constant miRNA synthesis rate, denoted as σ_c. Assuming an initial miRNA concentration of zero, the temporal evolution of miRNA concentration is given by:

$$r_c(t) = \frac{\sigma_c}{\gamma}\left(1 - e^{-\gamma t}\right) \tag{57}$$

Replacing this solution in the equation for R:

$$\frac{dR}{dt} = 1 - R - \kappa\frac{\sigma_c}{\gamma}\left(1 - e^{-\gamma t}\right)R \tag{58}$$

We recall the following result for linear ordinary differential equations:
Given f, g: $\mathbb{R} \to \mathbb{R}$ continuous functions, then all the solutions of the equation

$$\frac{dz}{dt} = f(t) + g(t)z(t) \tag{59}$$

are given by:

$$z(t) = e^{\int_0^t g(v)dv}\left(\int_0^t f(v)e^{-\int_0^v g(w)dw}dv + k\right) \tag{60}$$

with $k \in \mathbb{R}$ constant. If we have an initial datum $z(0) = z_0$, then the unique solution satisfying the equation 59 and the initial datum is:

$$z(t) = e^{\int_0^t g(v)dv}\left(\int_0^t f(v)e^{-\int_0^v g(w)dw}dv + z_0\right) \tag{61}$$

Thus, comparing eqs. 58 and 59 we find that the solution for eq. 58 is:

$$R(t) = e^{\int_0^t(-1-\frac{\kappa\sigma_c}{\gamma}(1-e^{-\gamma v}))dv}\left(\int_0^t 1e^{-\int_0^v(-1-\frac{\kappa\sigma_c}{\gamma}(1-e^{-\gamma w}))dw}dv\right) \tag{62}$$

Thus:

$$R(t) = \left(e^{(-1-\frac{\kappa\sigma_c}{\gamma})t+\frac{\kappa\sigma_c}{\gamma^2}(1-e^{-\gamma t})}\right)\int_0^t e^{-(-1-\frac{\kappa\sigma_c}{\gamma})v}e^{-\frac{\kappa\sigma_c}{\gamma^2}(1-e^{-\gamma v})}dv \tag{63}$$

which does not have a closed solution.
Nevertheless, we can numerically explore this simplified model to assess whether frequency preference and advantage still arise, helping to identify the minimal components necessary for these behaviors to emerge.
For this analysis, we revisit to the model described in eq. 54, which assumes fast complex recycling while retaining miRNA and RNA degradation within complex. To determine suitable parameter ranges for κ_1 and κ_2, we sampled the parameter space of model M1 by Latin Hypercube Sampling [14], imposing that the simplified model reaches the same RNA steady state as M1 under constant miRNA synthesis. Note that while both models share

the same steady state RNA concentration, their dynamics may differ.
Following the methodology outlined for model M1 in the Materials and Methods section, we examined the response of the simplified model. We computed the equilibration time (τ_{noC}) required for the RNA to reach steady state under constant miRNA synthesis, and evaluated fold repression for both constant and periodic synthesis cases. Results are summarized in Figure S2.
We first note that, in most cases, the equilibration time in the model without explicit complex (τ_{noC}) closely matches that of the full model (τ_{M1}), indicating that complex processing in M1 is generally already fast enough (see Figure S2b). Consequently, we expect the explored frequency ranges to be similar between the two models, except when complex processing is slow (i.e., for higher τ_2; see process timescale definition in section 6). In such cases, the explored range extends to lower frequencies than in the full model M1.
Panel S2a shows fold repression as a function of frequency for both the simplified and the full models across three different parameters sets. While the frequency responses of the two models can differ, they align when the assumption of fast complex processing holds in M1 as well (Figure S2a, middle panel). In this scenario, the timescales related to complex processing are at least an order of magnitude smaller than RNA and miRNA half-lives ($\tau_0 = 5.88, \tau_2 = 0.085$). Fold repression may also differ in absolute magnitude, as transient dynamics depends on whether the complex is explicitly included (Figure S2a, left panel).
Despite these differences, the simplified model still exhibits frequency preference, albeit with a more moderate advantage over constant miRNA synthesis compared to the full model. Panels S2c-e compare key frequency response metrics between the "fast complex model" (*no C*) and the original model M1. Selectivity remains similar in both models, particularly when complex degradation is fast. However, unlike the full model, the simplified version does not show a strong advantage across the parameter space.
As highlighted in the main text, advantage is closely linked to the target decay rate within complex (α) (see Table 1 in the main text), which amplifies the nonlinear effects of miRNA-target interaction. Since this amplification is absent in the model without explicit complex formation, the advantage over constant miRNA synthesis remains low. Notably, parameter sets that result in fast complex degradation (i.e., high τ_4; panel S2e) exhibit a similar preferred frequency for target repression in both models, whereas slower degradation in the complex shifts the preferred frequency to lower values. This is consistent with the correlation between f^* and the target decay rate in the complex (α; see Table 1 in the main text).

8. Frequency preference metrics as functions of parameters in model M1

To understand the role of parameters in determining frequency preference behaviors in model M1, we varied parameters in pairs by keeping the rest fixed to mean range values (section 5), and we computed FR_{const} and FR_{pulse} over the timespan $[0, \tau]$, where τ represents the RNA equilibration time under constant miRNA synthesis σ_{const}. We thus computed the three metric quantities S, A and f^* for each parameter combination. Figure S3 shows selectivity S as a function of M1 model parameter pairs, whereas Fig. S4 represents the advantage A and Fig. S5 the preferred frequency f^*.

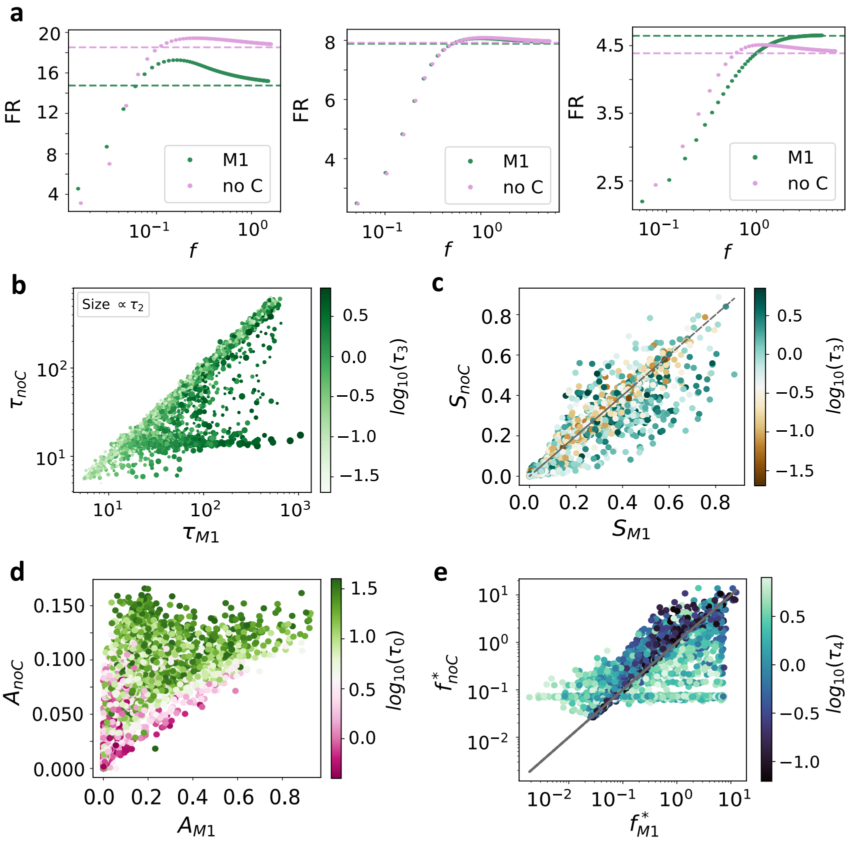

Figure S2: Comparison of frequency preference behaviors of the complete model M1 and the fast complex processing model. textbfa. Fold repression as a function of miRNA synthesis frequency for both models, the complete model outlined in the main text (*M1*) and the fast complex model (*no C*), for three different parameter sets. The horizontal dashed line represents the fold repression value given by constant miRNA synthesis in each model. Left panel: $\sigma = 0.29, \gamma = 0.22, \kappa_{off} = 51, \kappa_{on} = 758, \alpha = 2,55, \beta = 0.18$. Middle panel: $\sigma = 0.34, \gamma = 0.17, \kappa_{off} = 9.90, \kappa_{on} = 74.4, \alpha = 1.77, \beta = 0.29$. Right panel: $\sigma = 1.16, \gamma = 0.68, \kappa_{off} = 40, \kappa_{on} = 69, \alpha = 13, \beta = 5.53$ **b.** Scatter plot of equilibration times for the simpler model (τ_{noC}) and the complete model (τ_{M1}) with constant miRNA synthesis rate. Each scattered point corresponds to a parameter set; its size is related to the average lifetime of the complex τ_2, while its color is given by the average time it takes the complex to be degraded, τ_3. **c.** Scatter plot of selectivity for the fast complex processing model (S_{noC}) and selectivity for the complete model (S_{M_1}). Each scattered point corresponds to a parameter set. Colors of scattered points are proportional to τ_3, i.e. the average time necessary for the complex to be degraded and recycled into either miRNA or RNA. **d.** Scatter plot of advantage for the fast complex processing model (A_{noC}) and advantage for the complete model (A_{M_1}). Each scattered point corresponds to a parameter set. Colors of scattered points are proportional to τ_0, i.e. the time necessary for the RNA to be degraded relative to the miRNA. **e.** Scatter plot of preferred frequency for the fast complex processing model (f^*_{noC}) and preferred frequency for the complete model ($f^*_{M_1}$). Each scattered point corresponds to a parameter set. Colors of scattered points are proportional to τ_4, i.e. the average time necessary for the RNA to be degraded (and thus the miRNA to be recycled).

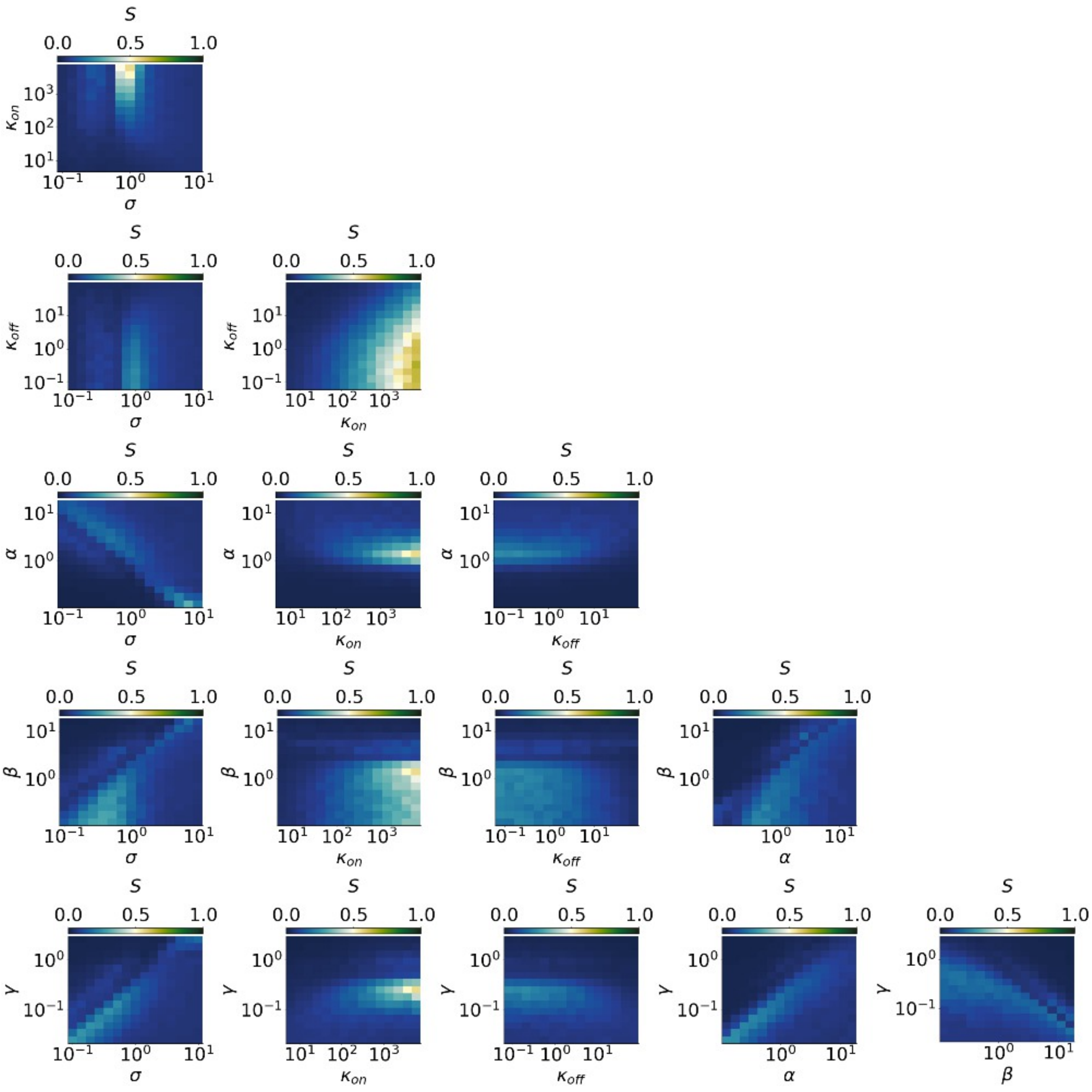

Figure S3: Selectivity S computed as a function of parameter pairs of model M1. Each plot represents S as a function of parameter values for a parameter pair of model M1.

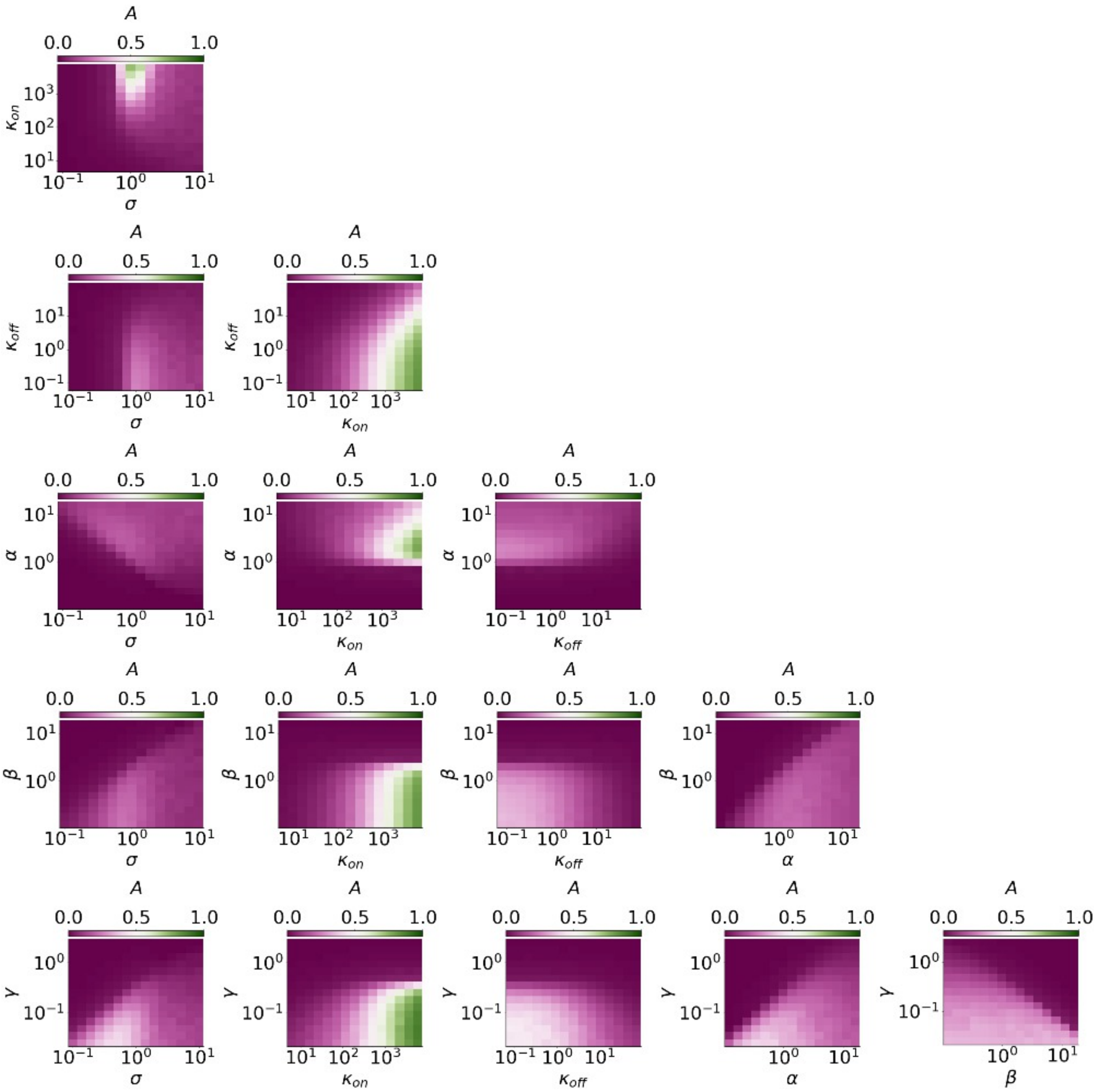

Figure S4: Advantage A computed as a function of parameter pairs of model M1. Each plot represents A as a function of parameter values for a parameter pair of model M1.

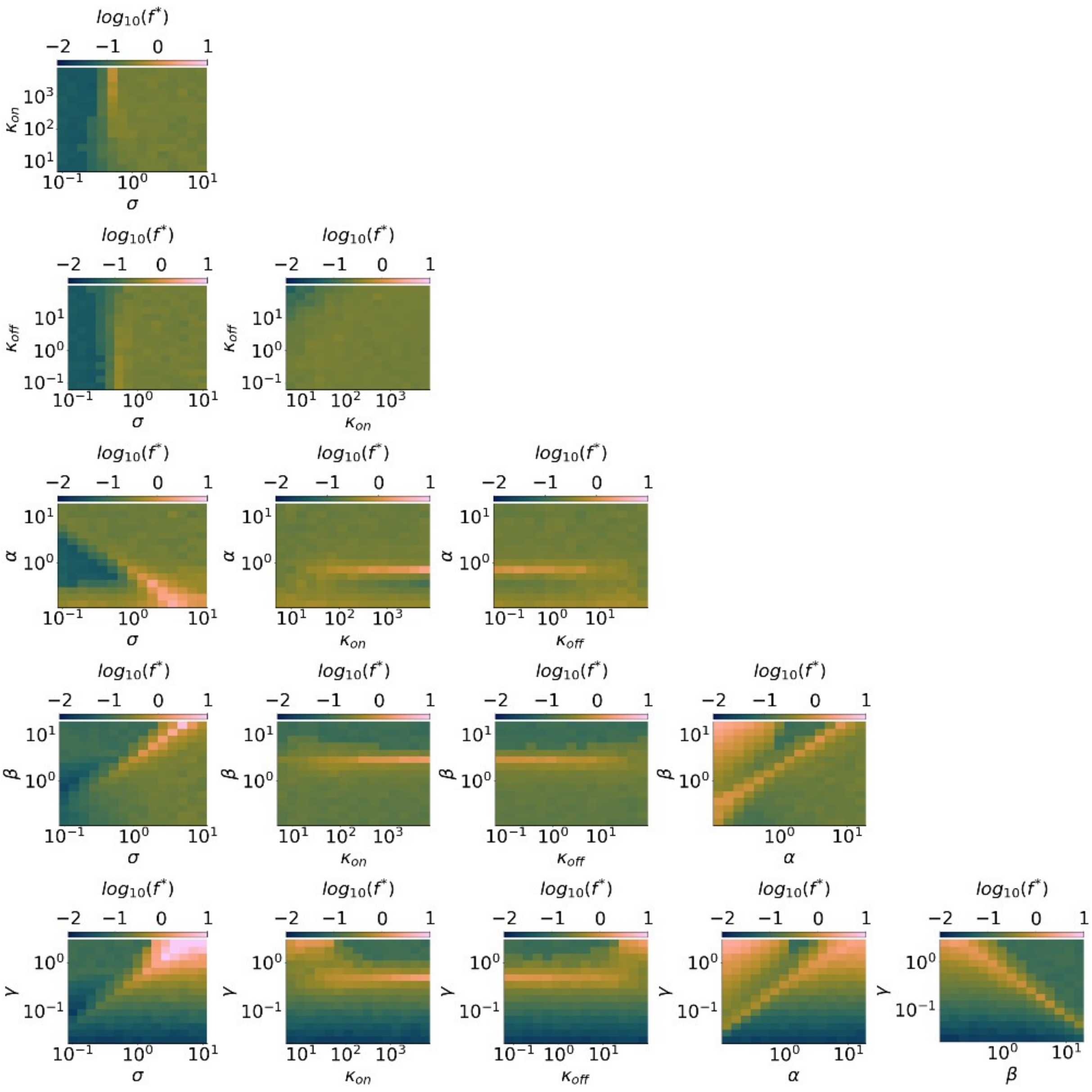

Figure S5: Preferred frequency f^* computed as a function of parameter pairs of model M1. Each plot represents f^* as a function of parameter values for a parameter pair of model M1.

9. Fold repression vs frequency at a later time stage

To understand how the frequency preference behavior changes if we measure fold repression at later time stages (i.e. beyond the RNA equilibration time τ), we computed FR_{const} and $FR_{pulse}(f)$ (see Materials and Methods section) for randomly sampled parameter sets of model M1 in both $[0, \tau]$ and in $[\tau, 2\tau]$ time intervals. Note that the latter timespan corresponds to the target RNA's steady state in the constant miRNA synthesis case (σ_{const}). Parameters were sampled randomly by Latin Hypercube Sampling ([14]).
By comparing FR_{const} and $FR_{pulse}(f)$ computed in the two different timespans (Figure S6) we found that the later timespan of observation, i.e. $[\tau, 2\tau]$, tends to drive the system to high-pass filtering behaviors. This is explained by the fact that a later time window of observation privileges higher frequencies, as miRNA accumulation generates the strongest repression at this stage.

10. Estimation of parameters in model M2

For parameters σ, κ_{on_i}, κ_{off_i}, α_i, β_i and γ we adopted ranges estimated for model M1 (see section 5). For the remaining parameters δ and ϵ - which represent relative synthesis and degradation rates of target R_2 with respect

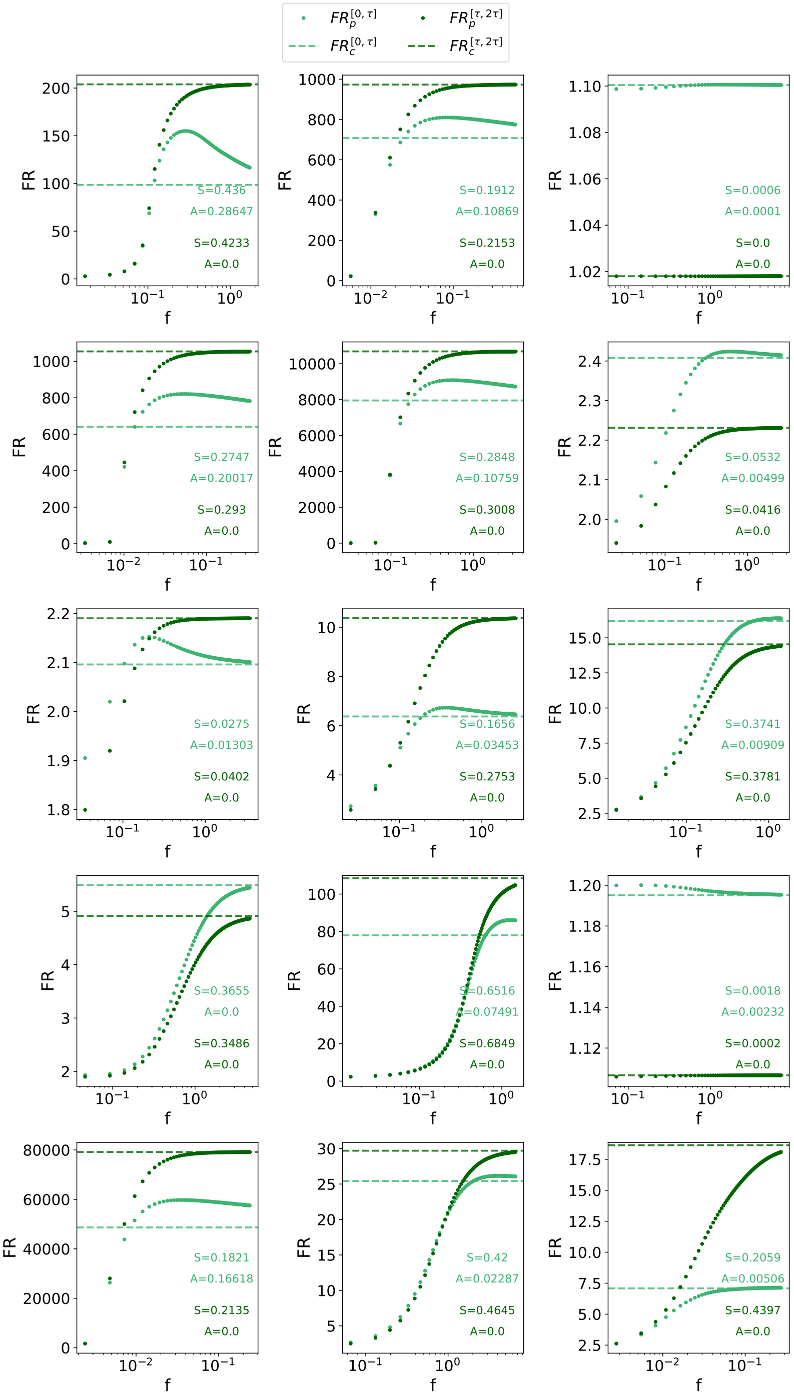

Figure S6: Fold repression as a function of frequency computed at earlier and later time stages. Each plot represents FR_{const} and $FR_{pulse}(f)$ computed for a randomly sampled parameter set of model M1 in time windows $[0, \tau]$ (respectively dark green and dark grey) and $[\tau, 2\tau]$ (respectively light green and light grey). Selectivity S and advantage A values are reported for the respective curves.

to target R_1 - we chose the range $[10^{-1}, 10^{1}]$. This allows to explore scenarios where one of the two targets is characterized by slower or faster synthesis and/or degradation kinetics with respect to the other.

Nondimensional parameter ranges for model M2 are reported in table S4. Note that $i = 1, 2$ indicates either one of the two RNA species.

Nondimensional parameter	Biological meaning	Range
σ	Scaled miRNA synthesis rate	$[10^{-1}, 10^1]$
κ_{on_i}	Scaled binding rate	$[6, 6 \times 10^3]$
κ_{off_i}	Scaled unbinding rate	$[7.7 \times 10^{-2}, 7.7 \times 10^1]$
α_i	Bound relative to unbound RNA degradation rate	[1/8, 16]
β_i	Bound relative to unbound miRNA degradation rate	[1/8, 16]
γ	Scaled miRNA degradation rate	$[10^{-1}/4, 10/4]$
δ	Relative target synthesis rate	$[10^{-1}, 10^1]$
ϵ	Relative target degradation rate	$[10^{-1}, 10^1]$

Table S4: Scaled M2 model's parameter ranges.

11. Frequency preference metrics as functions of competitor RNA parameters in model M2

To understand the role of competitor RNA's parameters in determining frequency preference behaviors of target R_1, we varied parameters of target R_2 in pairs by keeping the rest fixed to mean range values (section 10), and we computed FR^1_{const} and FR^1_{pulse} over the timespan $[0, \tau_1]$, where τ_1 represents the target R_1 equilibration time under constant miRNA synthesis σ_{const}. We thus computed the three metric quantities S^1, A^1 and f^{*1} for each competitor's parameter combination. Figure S7 shows selectivity S^1 as a function of M1 model parameter pairs, whereas Fig. S8 represents the advantage A^1 and Fig. S9 the preferred frequency f^{*^1}.

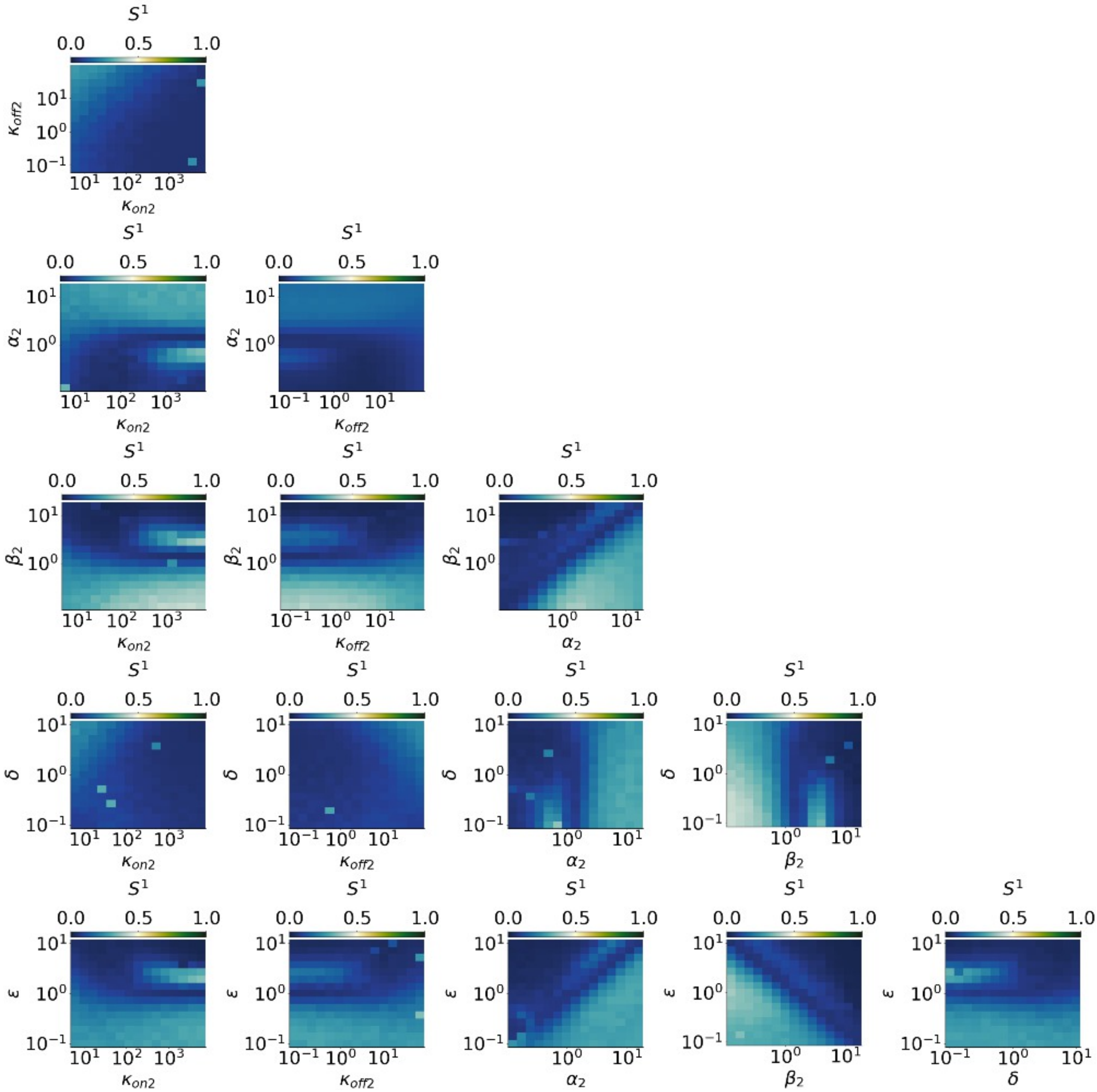

Figure S7: Selectivity of target R^1 computed as a function of parameter pairs relative to target R^2 in model M2. Each plot represents S^1 as a function of parameter values for a parameter pair of target R^2 in model M2.

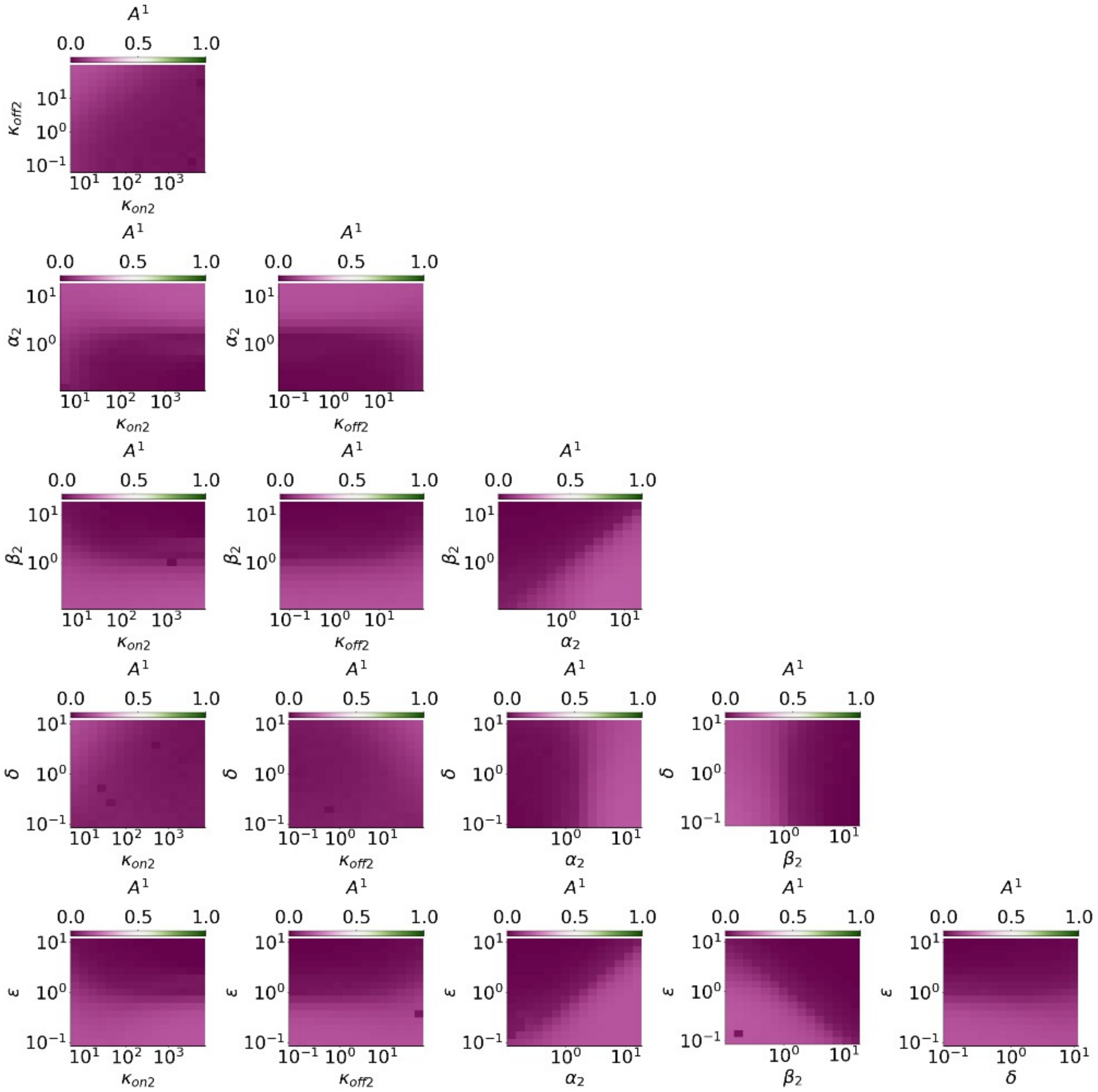

Figure S8: Advantage of target R_1, A^1, computed as a function of parameter pairs relative to target R_2 in model M2. Each plot represents A^1 as a function of parameter values for a parameter pair of target R_2 in model M2.

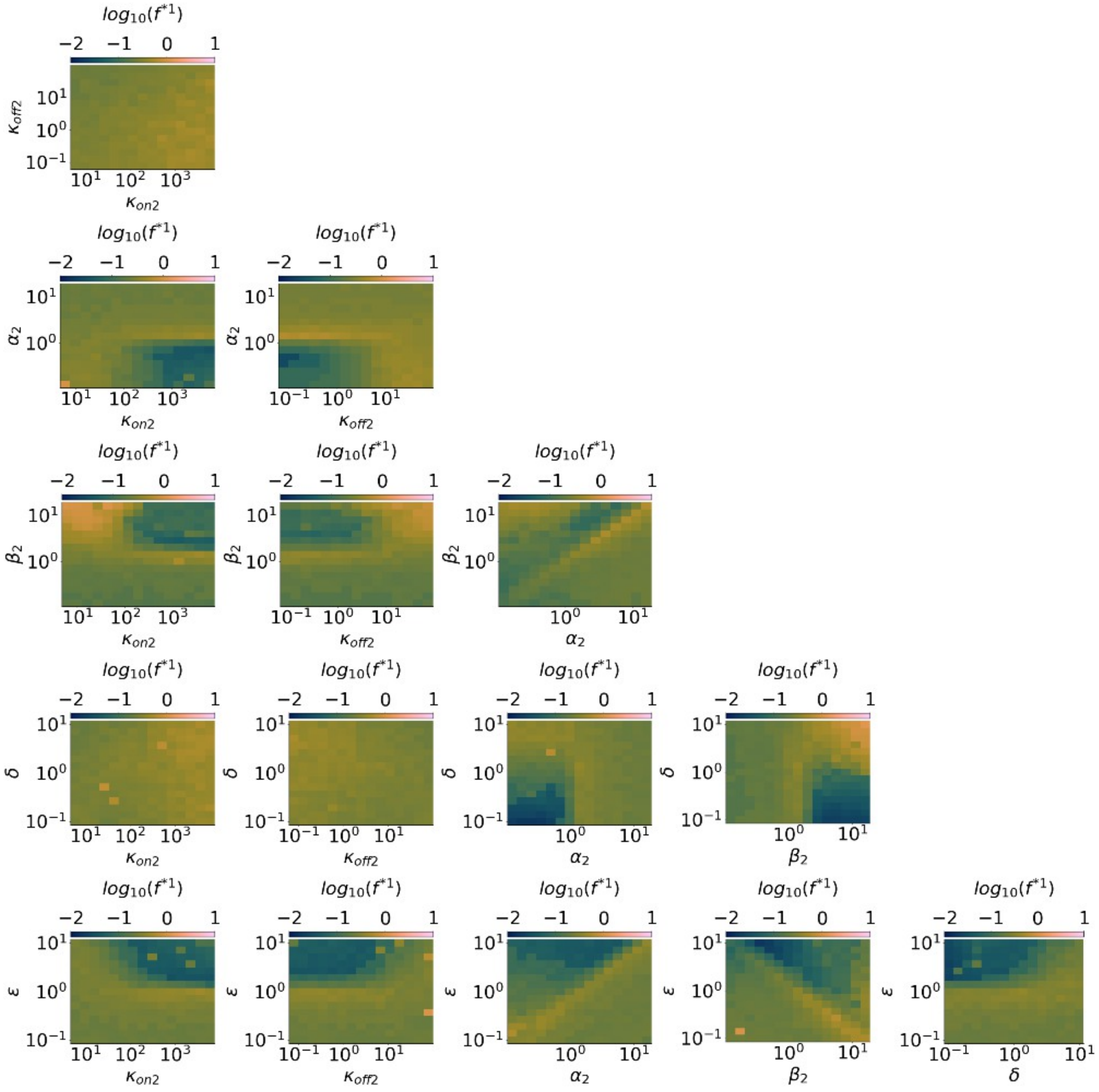

Figure S9: Preferred frequency of target R_1, f^{*1}, computed as a function of parameter pairs relative to target R_2 in model M2. Each plot represents f^{*1} as a function of parameter values for a parameter pair of target R_2 in model M2.

12. Sinusoidal vs square wave miRNA synthesis

In this section we compare the frequency preference response of model M1 obtained by modelling periodic miRNA synthesis as a square wave to the one obtained by modelling it as a sinusoidal function. To distinguish the two functional forms of miRNA synthesis rate, we will refer to them respectively as σ_{sqw} (otherwise named σ_{pulse}) for the square wave, and σ_{sin} for the sinusoidal wave. We recall that the first - defined and nondimensionalized in section 3 - reads as:

$$\sigma_{sqw} = \begin{cases} \sigma & \text{if } nT < t \leq (n+d)T, \quad n = 0, 1, 2, \ldots \\ 0 & \text{if } (n+d)T < t \leq (n+1)T, \quad n = 0, 1, 2, \ldots \end{cases} \tag{64}$$

whereas with analogous nondimensionalization steps we can derive the sinusoidal one which results as:

$$\sigma_{sin} = \sigma(d + d sin(\frac{2\pi}{T}t)) \tag{65}$$

where in both equations T represents the pulse period and d the duty cycle. Both periodic waves oscillate in the range $[0, \sigma]$.

After feeding the model with the two different waveforms, we compute the resulting fold repression as a function of frequency (see Materials and Methods section). We will refer to the two distinct fold repression profiles resulting from the two waveforms of miRNA synthesis as FR_{sqw} and FR_{sin}, whereas the fold repression value given by constant miRNA synthesis is called as usual FR_{const}.

Figure S10a shows an example of temporal target RNA concentration resulting from the different waveforms for a fixed model parameter set ($\sigma = 0.5, \gamma = 0.25, \kappa_{on} = 60, \kappa_{off} = 0.75, \alpha = 1.25, \beta = 1$). Plots from top to bottom refer respectively to: (i) its constitutive concentration, (ii) its concentration resulting from constant miRNA synthesis σ_{const}, (iii) its concentration resulting from periodic miRNA synthesis modelled as a square wave, i.e. σ_{sqw}, (iv) its concentration resulting from periodic miRNA synthesis modelled as a sinusoidal wave (i.e. σ_{sin}).

By computing fold repression as a function of frequency for two randomly chosen model parameter sets, we found that profiles resulting from periodic miRNA synthesis modelled as either a square wave, i.e. FR_{sqw}, or a sinusoidal wave, i.e. FR_{sin}, result similar (Figure S10b). To further confirm that the two modelling choices of periodic miRNA synthesis generate similar results, we also computed metric quantities selectivity S, advantage A and preferred frequency f^* as functions of two model parameters, the relative miRNA/RNA synthesis and degradation rates σ and γ (Figure S10c). The remaining rate constants were kept fixed to the median values of ranges defined in section 5. Despite some differences, profiles obtained for sinusoidal miRNA synthesis qualitatively recover those obtained for the square wave miRNA synthesis adopted for the main results of this article.

13. Fold repression at protein level

To address the case of messenger RNAs (mRNAs), in this section we extend our model M1 by adding protein production. In this extended model, whenever mRNAs are not bound to miRNAs, they can be translated into proteins with translation rate g_P. Moreover, as assumed for the other molecular species, proteins can be degraded with rate q_P. Following the nondimensionalization approach used for the baseline model M1 (see section 1), these additional reactions can be embedded the model by adding the following equation to eqs. 4:

$$\frac{dP}{dt} = gR - qP \tag{66}$$

where P represents the dimensionless protein concentration, g the dimensionless translation rate ($g = \frac{g_P}{k_R^0}$) and q the dimensionless degradation rate ($q = \frac{q_P}{k_R^0}$). Note that g and q have meanings respectively of protein production rate relative to the mRNA's degradation and relative protein-mRNA degradation rate. In agreement with [16], we consider realistic rate values for the protein, and we hereafter use them to compute ranges for the two nondimensional parameters g and q - analogous to the approach described in section 1. Proteins are on average more stable than mRNAs, as the protein median half-life is estimated to be 46 hours. However, half lives can vary from very short (less than 30 minutes) to very long (more than 200 hours). Accounting for parameter nondimensionalization (section 1), we estimate the following range for the dimensionless protein degradation rate q: [0.025, 2.5]. The median translation rate constant was estimated in [16] to be about 140 proteins per mRNA per hour. Consequent to nondimensionalization, we obtain the following range for the dimensionless translation rate g: [50,5000].

To analyze this extended model, we compute fold repression at the protein level for both constant and periodic miRNA synthesis with variable frequency. This analysis is based on identical criteria as those defined in the Materials and Methods section. Thus, we just briefly outline the definition of fold repression at the protein level and describe its computation as a function of frequency.

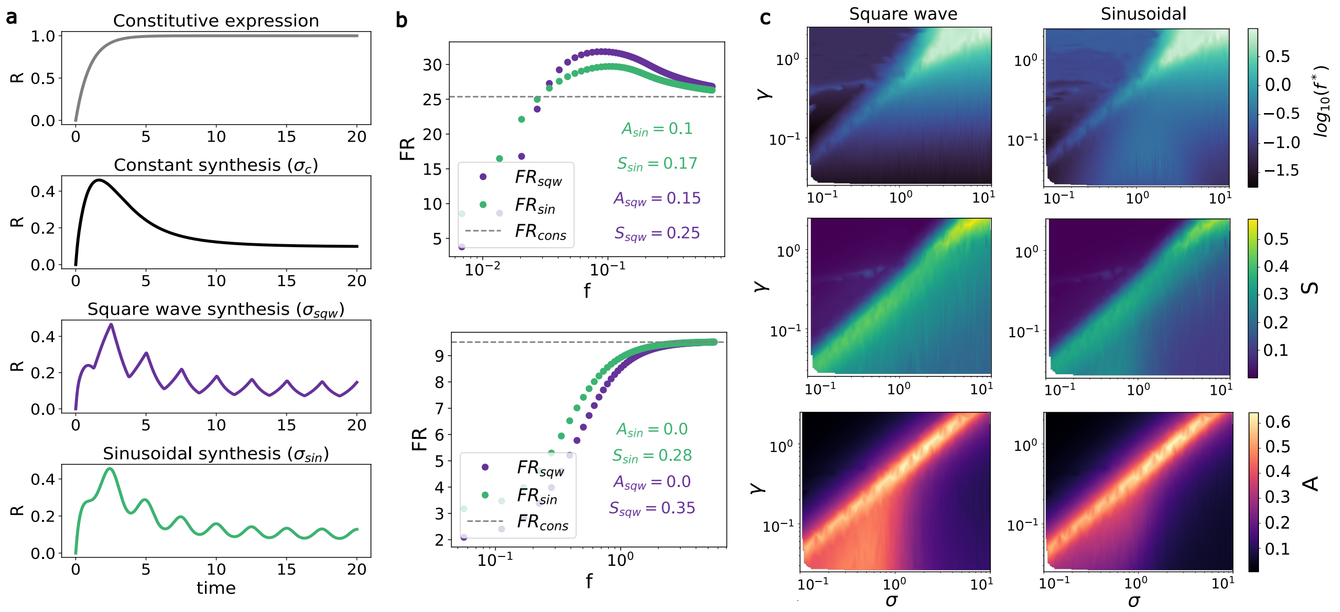

Figure S10: Different functional forms of periodic miRNA synthesis rate: square wave vs sinusoidal wave. **a.** Examples of temporal target RNA concentration given by dimensionless model M1, referred from the top to the bottom plot respectively to: constitutive expression (no miRNA interaction), constant miRNA synthesis (σ_{const}), periodic miRNA synthesis modelled as a square wave (σ_{sqw}), periodic miRNA synthesis modelled as a sinusoidal wave (σ_{sin}). **b.** Fold repression as a function of frequency given by square wave miRNA synthesis (FR_{sqw}, purple dots) and sinusoidal miRNA synthesis (FR_{sin}, green dots). The horizontal dashed line represents the fold repression value given by constant miRNA synthesis, FR_{const}. Top and bottom plots refer to two different model parameter sets (top: $\sigma = 0.25, \gamma = 0.1, \kappa_{on} = 250, \kappa_{off} = 10, \alpha = 2, \beta = 0.5$, bottom: $\sigma = 5, \gamma = 1.5, \kappa_{on} = 100, \kappa_{off} = 5, \alpha = 0.75, \beta = 0.75$). Both plots report advantage and selectivity values resulting from the two miRNA synthesis modelling choices, i.e. A_{sqw}, A_{sin}, S_{sqw} and S_{sin}. Purple and green vertical dashed lines indicate values of preferred frequency resulting from the two respective miRNA synthesis modelling choices. **c.** Metric quantities advantage A (bottom plots), selectivity S (middle plots), and preferred frequency f^* (top plots) as functions of the relative miRNA/RNA synthesis rate σ and the relative miRNA/RNA degradation rate γ, computed for periodic miRNA synthesis modelled as either a square wave (left) or a sinusoidal wave (right).

Mirroring the definition at the RNA level, protein fold repression (FR_p) is defined as the fold change between the average constitutive level of protein expression and its average level resulting from miRNA action:

$$Protein\ Fold\ Repression = \frac{\langle constitutive\ protein\ level \rangle}{\langle repressed\ protein\ level \rangle} \tag{67}$$

Thus, analogous to what defined at the RNA level, the protein fold repression computed in a timespan $[0, \tau]$ for constant (σ_{const}) and periodic miRNA synthesis (σ_{pulse}) is given by the two respective formulas:

$$FR_{P_{const}} = \frac{\langle P_{basal}(t) \rangle_{[0,\tau]}}{\langle P_{const}(t) \rangle_{[0,\tau]}} \tag{68}$$

$$FR_{P_{pulse}}(f) = \frac{\langle P_{basal}(t) \rangle_{[0,\tau]}}{\langle P_{pulse}(t) \rangle_{[0,\tau]}} \tag{69}$$

where $P_{basal}(t)$ represents the temporal concentration of protein obtained in the absence of miRNA (i.e. its constitutive concentration), $P_{const}(t)$ represents its repressed concentration induced by the constant miRNA synthesis rate σ_{const}, and $P_{pulse}(t)$ represents its repressed concentration induced by the periodic miRNA synthesis σ_{pulse}.
To compute $FR_{P_{pulse}}(f)$, we numerically solve the extended ODE system for different frequencies of periodic miRNA synthesis rate σ_{pulse}. Identical to the definition given in the Materials and Methods section, the frequencies suitable for exploration are constrained to the ones yielding an integer number of pulses in the time window of computation, and thus range from $f_{min} = 1/\tau$ to $f_{max} = 10^2/\tau$.
The aim of comparing model responses at the protein level to those at the RNA level poses into question the choice of the time window $[0, \tau]$ to consider for computation: τ, i.e. the time needed by the target to reach steady state in the constant miRNA synthesis case, can be computed either considering the steady state of target RNA concentration (as in the main text) or considering the steady state of the corresponding protein product. We thus refer to these two distinct timescales respectively as τ_R and τ_P, and we compute fold repression responses using both time windows $[0, \tau_R]$ and $[0, \tau_P]$, discussing their differences.
To explore how the protein's kinetics shapes the frequency response at protein fold repression level, we fixed parameters of model M1 to mean range values (see section 5) and we chose rate constants of protein synthesis and

degradation so as to model a "fast protein" case ($g = 12.5, q = 2.5$) and a "slow protein" case ($g = 1.25, q = 0.25$). The ratio between g and q was maintained constant to ensure that the two cases produce the same steady state protein concentration. Thus, we numerically computed $FR_{P_{const}}$ and $FR_{P_{pulse}}(f)$ in both time windows $[0, \tau_R]$ and $[0, \tau_P]$ for the "fast" and "slow" protein cases. As shown in panel S11a, frequency preference behaviors at the protein level can more or less faithfully mirror those detected at the mRNA level, depending on the protein's kinetics and on the timespan considered. As one might expect, rapid protein kinetics (i.e. the "fast" case) reproduces more faithfully the frequency-dependent repression profile detected at the corresponding coding RNA level. However, the fold repression profile depends also on the timespan over which fold repression is calculated - either the one referring to mRNA equilibration $[0, \tau_R]$ or the one referring to protein equilibration $[0, \tau_P]$: in the slow protein case, the greater separation between mRNA and protein timescales leads to a loss of frequency preference behavior in the longer timespan $[0, \tau_P]$ - a behavior that is instead retained in the fast case where the RNA and protein timescales are more similar.
Whether the protein's kinetics is faster or slower - and thus if its equilibration time τ_P is shorter or longer - is largely determined by the protein's degradation timescale, as shown by the relationship between τ_P and q obtained by fixing all model parameters to median range values (section 5) and by randomly sampling the protein synthesis rate g (panel S11b). Coherent with this, selectivity S, advantage A and preferred frequency f^* computed at the protein level tend to resemble those at the mRNA level if the protein is more unstable, i.e. for high q (panels S11c-e). In this situation, protein dynamics mirrors mRNA dynamics, and thus the frequency responses across timescales $[0, \tau_R]$ and $[0, \tau_P]$ become similar. This observation aligns with the fact that τ_P is correlated with the slowest timescale of the system: when the protein is faster than the mRNA, τ_P is constrained by τ_R, whereas when the protein half life is long, it is the latter - and thus the parameter q - that determines the protein's equilibration time τ_P (Figure S11b). As a consequence, longer protein half-lives lead to a shift in the range of frequencies explored and in the fold repression response (Figure S11e). In this case, the response over $[0, \tau_P]$ loses any advantage A, and recovers it for higher q, when the protein mimics the mRNA's behavior (Figure S11d).

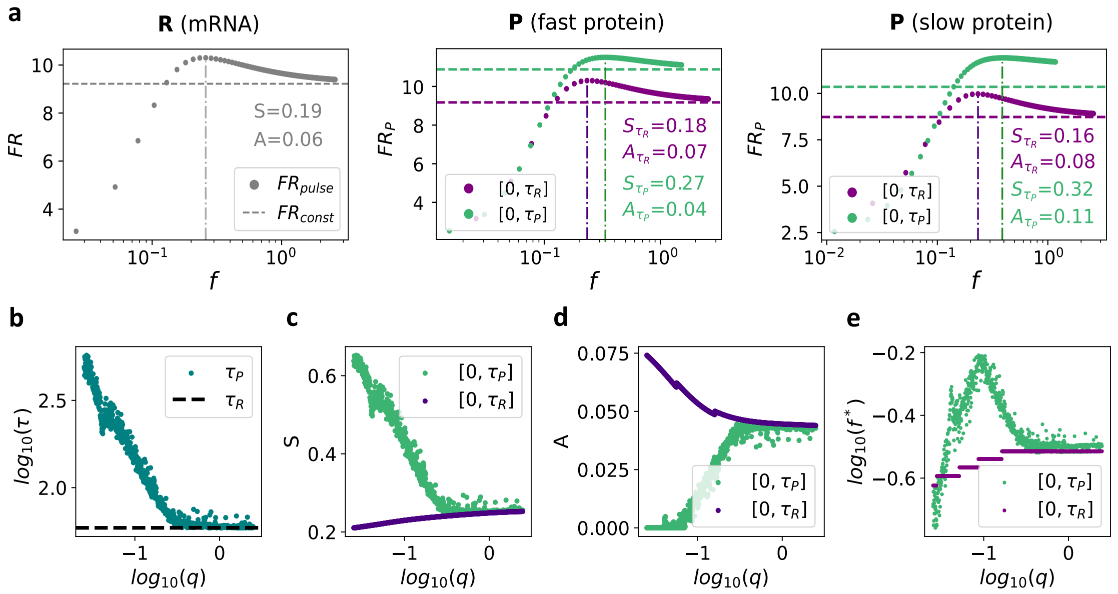

Figure S11: Fold repression at protein level for different levels of temporal separation between mRNA and protein dynamics. **a.** Fold repression at the level of mRNA (left plot), and at protein level, for a protein with fast kinetics - $g = 500$, $q = 2$ - (middle plot) and slow kinetics - $g = 50$, $q = 0.2$ - (right plot). Purple and green curves refer respectively to time intervals of computation $[0, \tau_R]$ (mRNA equilibration time with constant miRNA synthesis) and $[0, \tau_P]$ (protein equilibration time with constant miRNA synthesis). Note that the ratio between g and q is maintained constant in the fast and slow protein kinetics cases to maintain the protein's steady state concentration fixed. **b.** Metric quantities computed in the timespan $[0, \tau_R]$: preferred frequency (f^*), selectivity (S) and advantage (A) as functions of the protein's degradation (q) and translation (g) rates. **c.** Metric quantities computed in the timespan $[0, \tau_P]$: preferred frequency (f^*), selectivity (S) and advantage (A) as functions of the protein's degradation (q) and translation (g) rates. **b.** Protein's equilibration time τ_P with constant miRNA synthesis as a function of the protein's degradation rate constant q. Selectivity (panel **c**) , advantage (A) (**d**), and preferred frequency (f^*) (**e**) at the protein level as functions of the protein's degradation rate q computed over the mRNA equilibration time τ_R (violet) and over the protein equilibration time τ_P (green).

14. Synchronous pulses of miRNA and target RNA: an incoherent feedforward loop in the developing *C. elegans*

Kim and collegues [17] demonstrated that the oscillatory microRNA lin-4 participates in an incoherent feed-forward loop (iFFL) targeting lin-14, with both genes being transcriptionally activated by a shared, hypothetical master regulator. Inspired by their work, we consider a scenario in which both a miRNA and one of its targets are periodically and synchronously transcribed by the same master regulator.

To model the iFFL, we consider a variation of model M1 in which both the miRNA and the RNA target are periodically expressed. These two species are co-transcribed by the same master regulator, meaning their synthesis occurs in synchrony with identical frequency and duty cycle, although their amplitudes may differ. Accordingly, we represent the time-dependent synthesis rates of the miRNA and the target, denoted $S_r(t)$ and $S_R(t)$, as square-wave pulse trains with period $\hat{T}$ (i.e. frequency $\hat{f} = 1/\hat{T}$), fixed duty cycle $d = 0.5$, and amplitudes S_r and S_R:

$$S_r(\hat{t}) = \begin{cases} S_r & \text{if } n\hat{T} < \hat{t} \leq (n+d)\hat{T}, \quad n = 0,1,2,\ldots \\ 0 & \text{if } (n+d)\hat{T} < \hat{t} \leq (n+1)\hat{T}, \quad n = 0,1,2,\ldots \end{cases} \tag{70}$$

$$S_R(\hat{t}) = \begin{cases} S_R & \text{if } n\hat{T} < \hat{t} \leq (n+d)\hat{T}, \quad n = 0,1,2,\ldots \\ 0 & \text{if } (n+d)\hat{T} < \hat{t} \leq (n+1)\hat{T}, \quad n = 0,1,2,\ldots \end{cases} \tag{71}$$

Then, the dimensional iFFL model is described by equations identical to model M1 (eqs. 2), which we report here for ease of reference:

$$\begin{cases} \frac{d\hat{R}}{d\hat{t}} = S_R(t) - k_{on}\hat{R}\hat{r} + k_{off}\hat{C} - k_R^0\hat{R} + k_r^1\hat{C} \\ \frac{d\hat{r}}{d\hat{t}} = S_r(t) - k_{on}\hat{R}\hat{r} + k_{off}\hat{C} - k_r^0\hat{r} + k_R^1\hat{C} \\ \frac{d\hat{C}}{d\hat{t}} = k_{on}\hat{R}\hat{r} - k_{off}\hat{C} - k_R^1\hat{C} - k_r^1\hat{C} \end{cases} \tag{72}$$

To remain consistent with scaling choices made for model M1, we nondimensionalize this system using the RNA degradation rate k_R^0 and its mean synthesis rate over a period S_R^m, which is given by:

$$S_R^m = \frac{1}{\hat{T}}\int_0^{\hat{T}} S_R(t)dt = S_R.d = \frac{S_R}{2}$$

Then, the nondimensionalized system of equations reads:

$$\begin{cases} \frac{dr}{dt} = \sigma_p(t) - \gamma r + \kappa_{on} rR - \kappa_{off}C + \alpha C \\ \frac{dR}{dt} = \Sigma_p(t) - R + \kappa_{on} rR - \kappa_{off}C + \beta\gamma C \\ \frac{dC}{dt} = -\kappa_{on} rR + \kappa_{off}C - \alpha C - \beta\gamma C \end{cases} \tag{73}$$

where σ_p and Σ_p represent nondimensional periodic synthesis rates of the miRNA and the target RNA:

$$\sigma_p(t) = \begin{cases} \frac{S_r}{S_R^m} = \sigma & \text{if } nT < t \leq (n+d)T, \quad n = 0,1,2,\ldots \\ 0 & \text{if } (n+d)T < t \leq (n+1)T, \quad n = 0,1,2,\ldots \end{cases} \tag{74}$$

$$\Sigma_p(t) = \begin{cases} \frac{S_R}{S_R^m} = \frac{1}{d} & \text{if } nT < t \leq (n+d)T, \quad n = 0,1,2,\ldots \\ 0 & \text{if } (n+d)T < t \leq (n+1)T, \quad n = 0,1,2,\ldots \end{cases} \tag{75}$$

with the following definitions: $\sigma = \frac{S_r}{S_R^m}$, $\kappa_{on} = \frac{k_{on}S_R^m}{k_R^0}$ and the remaing dimensionless model parameters are provided in eq. 5. Coherently with model M1, in this nondimensionalized iFFL model the mean RNA transcription rate S_R^m is scaled to 1 and the conservation of the relative miRNA-to-target dose is given by:

$$\frac{\int_0^T S_r(t)dt}{\int_0^T S_R(t)dt} = \frac{S_r d}{S_R^m} = \sigma.d \tag{76}$$

Thus, identical to what was described in the Materials and Methods section for model M1, dose conservation is guaranteed if both σ and d are kept fixed.

To assess how the average target fold repression, FR, responds to different frequencies, we use the same approach described in the Materials and Methods section. In particular, we are interested in comparing FR vs frequency curves resulting from four different scenarios with equivalent parameter values:

(i). the miRNA and the target RNA are part of the periodically forced iFFL, i.e. they are synthesized in synchronous pulses. Thus, fold repression is defined as:

$$FR_{iFFLpulse} = \frac{\langle R_{iFFLbasal}(t)\rangle_{[0,\tau]}}{\left\langle R_{iFFLpulse}(t)\right\rangle_{[0,\tau]}}$$

where $R_{iFFLbasal}(t)$ represents the temporal target RNA's concentration in the absence of the miRNA (i.e., its constitutive concentration under periodic synthesis driven by the hypothetical master regulator); $R_{iFFLpulse(t)}$ describes the temporal concentration of the target RNA repressed by the synchronously transcribed miRNA.

(ii). the target RNA is still transcribed in pulses by a hypothetical regulator, whereas the miRNA is independently expressed at a constant rate (i.e., it is not embedded in the iFFL). In this case, fold repression reads:

$$FR_{iFFLconst} = \frac{\langle R_{iFFLbasal}(t)\rangle_{[0,\tau]}}{\langle R_{iFFLconst}(t)\rangle_{[0,\tau]}}$$

where $R_{iFFLbasal}(t)$ represents the target RNA's concentration in the absence of the miRNA; $R_{iFFLconst(t)}$ denotes the temporal concentration of the target RNA repressed by a constantly transcribed miRNA, i.e. a miRNA which is not part of the iFFL. By comparing $FR_{iFFLpulse}$ to $FR_{iFFLconst}$, we aim to capture the difference - in terms of average target repression vs frequency - between an iFFL-embedded miRNA (i.e. one that is transcribed in pulses synchronous to the target) and a miRNA that is independently transcribed at a constant rate.

(iii). as in model M1, the target RNA is constantly transcribed, whereas miRNA synthesis is periodic. In this case, fold repression reads:

$$FR_{M1} = \frac{\langle R_{basal}(t)\rangle_{[0,\tau]}}{\left\langle R_{pulse}(t)\right\rangle_{[0,\tau]}}$$

where, matching the main text of the manuscript, $R_{basal}(t)$ represents the temporal target RNA concentration in the absence of the miRNA (i.e., its constitutive concentration under constant synthesis), and $R_{pulse}(t)$, describes the time-dependent concentration of the target RNA repressed by a periodically transcribed miRNA. By comparing $FR_{iFFLpulse}$ to FR_{M1}, we aim to compare the frequency-dependent behavior of the periodically forced iFFL to the one resulting from our main model M1 - where miRNA and target syntheses are independent and only the repressor is synthesized periodically.

(iv). as in the constant synthesis case of model M1, both miRNA and target are constantly transcribed. Fold repression thus reads:

$$FR_{M1const} = \frac{\langle R_{basal}(t)\rangle_{[0,\tau]}}{\langle R_{const}(t)\rangle_{[0,\tau]}}$$

where, matching the main text of the manuscript, $R_{basal}(t)$ represents the temporal target RNA concentration in the absence of the miRNA (i.e., its constitutive concentration under constant synthesis), and $R_{const}(t)$ describes the time-dependent concentration of the target RNA repressed by a constantly transcribed miRNA.

We adopt a similar approach to address how the dampening of target RNA oscillations depends on frequency: we compute the average reduction in amplitude, named RA, as the ratio between the average amplitude of target pulses in unrepressed (i.e., when the target is not interacting with the miRNA) and repressed cases. We are interested in comparing the RA vs frequency curves resulting from above scenarios (i) and (ii) (not (iii) nor (iv), as in those cases the target RNA is not periodically transcribed and thus the amplitude reduction observable loses its meaning):

(i). in this case, we define the amplitude reduction as:

$$RA_{iFFL\,pulse} = \frac{\langle a_{iFFLbasal}(t)\rangle_{[0,\tau]}}{\left\langle a_{iFFLpulse}(t)\right\rangle_{[0,\tau]}}$$

where $\langle a_{iFFLbasal}(t)\rangle_{[0,\tau]}$ is the average amplitude of target RNA pulses (i.e., the mean difference between consecutive minima and maxima across pulses) over the interval $[0,\tau]$, in the absence of the miRNA (i.e., for an unrepressed target RNA), and $\left\langle a_{iFFLpulse}(t)\right\rangle_{[0,\tau]}$ is the average amplitude of target RNA pulses when it interacts with its synchronously transcribed repressor.

(ii). in this case, the amplitude reduction is defined as:

$$RA_{iFFLconst} = \frac{\langle a_{iFFLbasal}(t)\rangle_{[0,\tau]}}{\langle a_{iFFLconst}(t)\rangle_{[0,\tau]}}$$

where $\langle a_{iFFLbasal}(t)\rangle_{[0,\tau]}$ is the average amplitude of target RNA pulses in the absence of the miRNA (i.e., for an unrepressed target RNA); and $\langle a_{iFFLcons}(t)\rangle_{[0,\tau]}$ is the mean amplitude of target RNA pulses when it interacts with a constantly synthesized repressor, i.e., a miRNA that is not embedded in the iFFL.

By computing $RA_{iFFLpulse}$ and $RA_{iFFLconst}$, we compare the capability to dampen RNA target oscillations of a periodically forced incoherent feed-forward loop (iFFL) versus an independently transcribed miRNA at a constant rate.

To compute the defined fold repression (FR) and amplitude reduction (RA) observables as functions of frequency, we chose a parameter set compatible with the ranges estimated for model M1 in Supplementary data section 5 ($\sigma = 0.5, \quad \gamma = 0.25, \quad \kappa_{on} = 650, \quad \kappa_{off} = 2.25, \quad \alpha = 2, \quad \beta = 1.5$) and we computed the time τ necessary for the target RNA to reach steady state in the constant synthesis case (i.e., when both miRNA and target are transcribed with equivalent constant rates, corresponding to $\sigma/2$ and 1). Then, following the usual approach, we defined the frequency range compatible with τ (i.e., yielding integer numbers of pulses in the interval $[0, \tau]$) as $[1/\tau, 10^2/\tau]$.

For each frequency, we computed both FR and RA under the described scenarios. The three plots in Figure S12a correspond to three different frequencies and show temporal trajectories of the periodically transcribed target within the iFFL: its constitutive concentration $R_{iFFLbasal}$, its concentration when repressed by a synchronously transcribed miRNA $R_{iFFLpulse}$, and its concentration when repressed by an independent constantly transcribed miRNA, $R_{iFFLcons}$. We found that in the $[0, \tau]$ interval the fold repression $FR_{iFFLpulse}$ presents a minimum for a given frequency of miRNA and target synthesis, in contrast to the frequency preference behavior characterizing model M1, FR_{M1}, which presents a maximum in line with results shown in the main text (Figure S12b, top left plot). Note that while the minimum is maintained also in the later stage $[\tau, 2\tau]$ for $FR_{iFFLpulse}$, FR_{M1} loses its frequency preference, coherent with what presented in our main results (Figure S12, top right plot). Concomitantly, $FR_{iFFLcons}$ exhibits a high-pass filtering behavior taking values lower than $FR_{iFFLpulse}$ for any frequency both in the system's transient interval $[0, \tau]$ and in the steady state $[\tau, 2\tau]$ (Figure S12, top plots). Predictably, this shows that an independent constantly transcribed miRNA is less efficient in repressing the periodically synthesized target with respect to an iFFL-embedded miRNA whose transcription is constrained to be synchronous to that of the target.

Interestingly, the amplitude reduction $RA_{iFFLpulse}$ is higher than $RA_{iFFLcons}$ and displays frequency preference, peaking at a specific synthesis frequency in both transient $[0, \tau]$ and steady state $[\tau, 2\tau]$ intervals (Figure S12b, bottom plots). Note that the same frequency generated an "inverse optimality" in fold repression $FR_{iFFLpulse}$ (Figure S12, top plots), appearing as the least efficient frequency in repressing the target. The fact that a synchronously transcribed miRNA exhibits optimality in dampening the target's oscillations for a given frequency - and while simultaneously maintaining the mildest repression extent - is well in line with the iFFL's known role in buffering fluctuations while exerting a modest repressive action on its target gene [18; 19]. Moreover, it is interesting to note that within the iFFL these frequency preference behaviors are conserved also at later time stages of the interaction, unlike those presented for the simple miRNA-target interaction in our main results. This preliminary result thus supports the capability of reducing target oscillation amplitudes as a key property of the periodically forced iFFL, as highlighted in [17]. It also suggests that the dampening capability may be frequency-selective. However, a deeper analysis and parameter sampling are needed to fully characterize this behavior and its relationship to the underlying reaction kinetics. It would be of interest to investigate whether specific oscillation frequencies - such as those experimentally observed during *C. elegans* development [17] - may have been subject to selection due to their effectiveness in reducing periodic fluctuations.

Notably, if we add on top of the iFFL a second target sharing the same miRNA (i.e., as in model M2) but constantly transcribed in an independent way, with identical affinity and half-life, we obtain opposed frequency-dependent fold repression behaviors (Figure S12c): while FR_1, which is relative to the iFFL-embedded target, exhibits a minimum, FR_2, which refers to the independently transcribed target, presents a maximum at a similar frequency value. This observation is in line with the possibility of exclusive repression on a selected target despite the miRNA being shared among multiple RNA species, as shown in the main results of the manuscript. While exclusive target regulation by an oscillatory miRNA was linked to differences in the two RNA's timescales, here we show a case where differential regulation can also arise even when targets exhibit similar kinetics. In a non-oscillatory context, both targets would experience a similar repression, however under dynamic conditions, the network architecture enables selective modulation.

Moreover, at the same frequency value where one target's fold repression peaks and the iFFL-embedded one

throughs, the latter also displays maximal amplitude reduction (Figure S12c, blue curves). This highlights a dual miRNA action: on one side, it buffers fluctuations of the iFFL-embedded target with minimal repression, while on the other side it effectively inhibits the mean expression of the independently transcribed target.

Therefore, it would be interesting to investigate more in depth whether embedding one of multiple miRNA-sharing targets in a periodically forced iFFL might represent a robust way to isolate it from excessive repression at a mean value level, while efficiently suppressing its fluctuations.

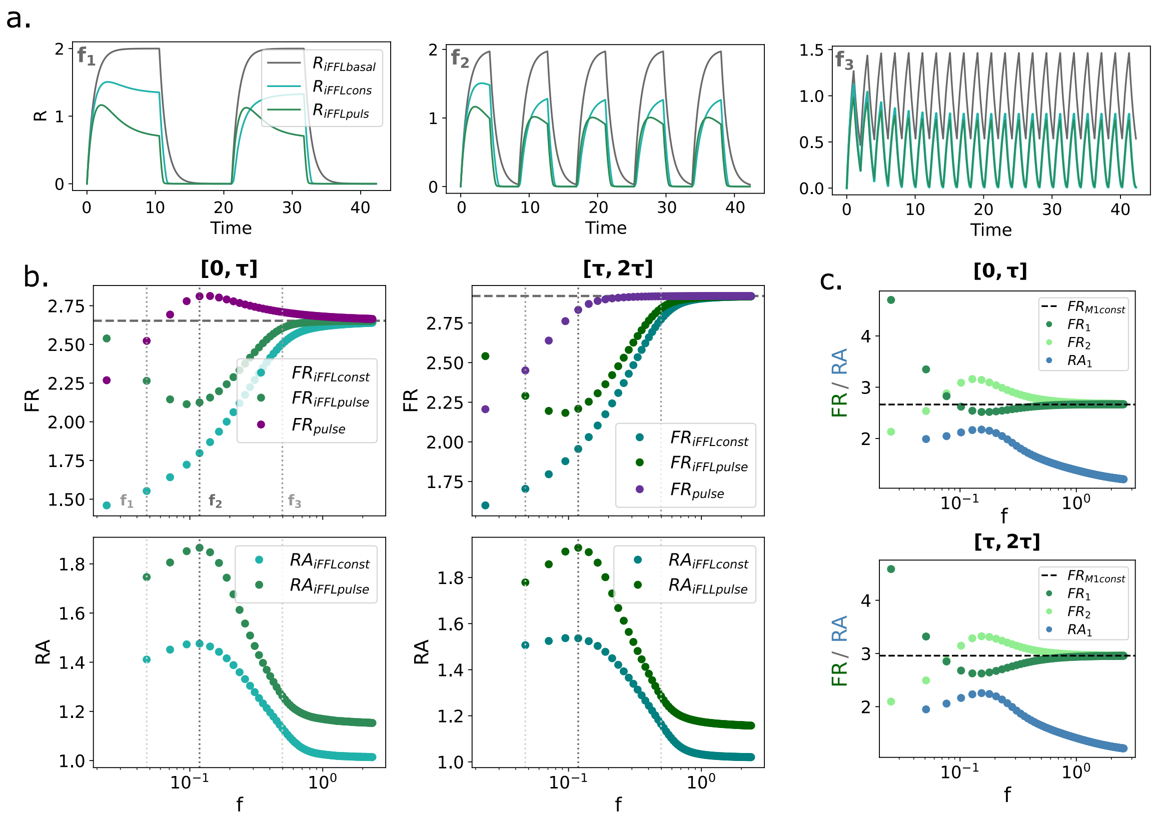

Figure S12: **Frequency preference behaviors generated by synchronous periodic synthesis of miRNA and target RNA.a.** Temporal trajectories of target RNA concentration within a periodically forced iFFL: target RNA in the absence of miRNA (i.e., constitutive concentration, grey curve); target RNA repressed by and independent constantly transcribed miRNA (i.e. a miRNA not embedded in the iFFL, light blue curve); target RNA repressed by a synchronously transcribed miRNA (i.e. an iFFL-embedded one, green curve). Each plot is referred to a different input frequency. **b.** Top plots: fold repression (FR) vs frequency. Green dots represent $FR_{iFFLpulse}$, i.e. fold repression computed in the $[0, \tau]$ interval (left panel) and in the $[\tau, 2\tau]$ interval (right panel) for a target repressed by an iFFL-embedded miRNA with synchronous periodic synthesis; Blue dots represent $FR_{iFFLconst}$, i.e. fold repression computed in the $[0, \tau]$ interval (left panel) and in the $[\tau, 2\tau]$ interval (right panel) for a target repressed by an independent constantly transcribed miRNA; Purple dots represent FR_{M1}, i.e. fold repression computed in the $[0, \tau]$ interval (left panel) and in the $[\tau, 2\tau]$ interval (right panel) for a constantly transcribed target repressed by an independent periodically transcribed miRNA (corresponding to model M1); the black dashed line represents $FR_{M1const}$, i.e. fold repression computed in the $[0, \tau]$ interval (left panel) and in the $[\tau, 2\tau]$ interval (right panel) with constantly transcribed miRNA and target (corresponding to the constant synthesis case in model M1). Bottom plots: amplitude reduction (RA) vs frequency. Green dots represent $RA_{iFFLpulse}$, i.e. amplitude reduction for a target repressed by an iFFL-embedded miRNA with synchronous periodic synthesis, computed in the $[0, \tau]$ interval (left plot) and in the $[\tau, 2\tau]$ interval (right plot); Blue dots represent $RA_{iFFLconst}$, i.e. amplitude reduction for a target repressed by an independent constantly transcribed miRNA, computed in the $[0, \tau]$ interval (left plot) and in the $[\tau, 2\tau]$ interval (right plot). Parameter values used are: $\sigma = 0.25$, $\gamma = 0.25$, $\kappa_{on} = 650$, $\kappa_{off} = 2.25$, $\alpha = 2, \beta = 1.5$. **c.** Dark and light green curves represent fold repression (FR) vs frequency computed in the $[0, \tau]$ interval (top plot) and in the $[\tau, 2\tau]$ interval (bottom plot) for two target RNA species that share the same miRNA: R_1 (dark green), which forms an iFFL with the miRNA (i.e. is expressed in periodic pulses synchronous to the miRNA), and R_2 (light green), which is independent from the iFFL and constantly transcribed. The blue curve represents amplitude reduction (RA) for target R_1 computed in the $[0, \tau]$ interval (top plot) and in the $[\tau, 2\tau]$ interval (bottom plot). Used parameter values are: $\sigma = 0.25$, $\gamma = 0.25$, $\kappa_{on1} = \kappa_{on2} = 650$, $\kappa_{off1} = \kappa_{off2} = 2.25$, $\alpha_1 = \alpha_2 = 2, \beta_1 = \beta_2 = 1.5$, $\epsilon = 1$, $\delta = 1$.

References

[1] Benjamin Nordick, Polly Y. Yu, Guangyuan Liao, and Tian Hong. Nonmodular oscillator and switch based on RNA decay drive regeneration of multimodal gene expression. *Nucleic Acids Research*, 50(7):3693–3708, 2022.

[2] Martin Feinberg. Chemical reaction network structure and the stability of complex isothermal reactors—ii. multiple steady states for networks of deficiency one. *Chem. Eng. Sci.*, 43:1–25, 1988.

[3] Marc Bodson. Explaining the routh–hurwitz criterion: A tutorial presentation [focus on education]. *IEEE Control Systems Magazine*, 40:45–51, 2020.

[4] Lioudmila V. Sharova, Alexei A. Sharov, Timur Nedorezov, Yulan Piao, Nabeebi Shaik, and Minoru S.H. Ko. Database for mRNA half-life of 19 977 genes obtained by DNA microarray analysis of pluripotent and differentiating mouse embryonic stem cells. *DNA Research*, 16(1):45–58, 2 2009.

[5] Ron Milo, Paul Jorgensen, Uri Moran, Griffin Weber, and Michael Springer. BioNumbers The database of key numbers in molecular and cell biology. *Nucleic Acids Research*, 38(SUPPL.1), 10 2009.

[6] Carla Bosia, Francesco Sgrò, Laura Conti, Carlo Baldassi, Davide Brusa, Federica Cavallo, Ferdinando Di Cunto, Emilia Turco, Andrea Pagnani, and Riccardo Zecchina. *RNAs competing for microRNAs mutually influence their fluctuations in a highly non-linear microRNA-dependent manner in single cells*, volume 18. 2017.

[7] Cem Albayrak, Christian A. Jordi, Christoph Zechner, Jing Lin, Colette A. Bichsel, Mustafa Khammash, and Savaş Tay. Digital Quantification of Proteins and mRNA in Single Mammalian Cells. *Molecular Cell*, 61(6):914–924, 3 2016.

[8] Brian Reichholf, Veronika A. Herzog, Nina Fasching, Raphael A. Manzenreither, Ivica Sowemimo, and Stefan L. Ameres. Time-Resolved Small RNA Sequencing Unravels the Molecular Principles of MicroRNA Homeostasis. *Molecular Cell*, 75(4):756–768, 8 2019.

[9] Matteo J. Marzi, Francesco Ghini, Benedetta Cerruti, Stefano De Pretis, Paola Bonetti, Chiara Giacomelli, Marcin M. Gorski, Theresia Kress, Mattia Pelizzola, Heiko Muller, Bruno Amati, and Francesco Nicassio. Degradation dynamics of micrornas revealed by a novel pulse-chase approach. *Genome Research*, 26(4):554–565, 4 2016.

[10] William E. Salomon, Samson M. Jolly, Melissa J. Moore, Phillip D. Zamore, and Victor Serebrov. Single-Molecule Imaging Reveals that Argonaute Reshapes the Binding Properties of Its Nucleic Acid Guides. *Cell*, 162(1):84–95, 7 2015.

[11] Liang Meng Wee, C. Fabián Flores-Jasso, William E. Salomon, and Phillip D. Zamore. Argonaute divides Its RNA guide into domains with distinct functions and RNA-binding properties. *Cell*, 151(5):1055–1067, 11 2012.

[12] Stephen W. Eichhorn, Huili Guo, Sean E. McGeary, Ricard A. Rodriguez-Mias, Chanseok Shin, Daehyun Baek, Shu hao Hsu, Kalpana Ghoshal, Judit Villén, and David P. Bartel. MRNA Destabilization Is the dominant effect of mammalian microRNAs by the time substantial repression ensues. *Molecular Cell*, 56(1):104–115, 2014.

[13] Manuel de la Mata, Dimos Gaidatzis, Mirela Vitanescu, Michael B Stadler, Corinna Wentzel, Peter Scheiffele, Witold Filipowicz, and Helge Großhans. Potent degradation of neuronal mi RNA s induced by highly complementary targets . *EMBO reports*, 16(4):500–511, 4 2015.

[14] Michael D McKay. Latin hypercube sampling as a tool in uncertainty analysis of computer models. In *Proceedings of the 24th conference on Winter simulation*, pages 557–564, 1992.

[15] Jingkui Wang, Laura Symul, Jake Yeung, Cédric Gobet, Jonathan Sobel, Sarah Lück, Pål O. Westermark, Nacho Molina, and Felix Naef. Circadian clock-dependent and -independent posttranscriptional regulation underlies temporal mrna accumulation in mouse liver. *Proceedings of the National Academy of Sciences*, 115(8):E1916–E1925, 2018.

[16] Björn Schwanhäusser, Dorothea Busse, Na Li, Johannes Schuchhardt, Jana Wolf, Wei Chen, and Matthias Selbach. Correction:corrigendum: Global quantification of mammalian gene expression control. *Nature*, (495):126–127, 2013.

[17] Dong Hyun Kim, Dominic Grün, and Alexander van Oudenaarden. Dampening of expression oscillations by synchronous regulation of a microrna and its target. *Nature genetics*, 45(11):1337–1344, 2013.

[18] Matteo Osella, Carla Bosia, Davide Corá, and Michele Caselle. The role of incoherent microrna-mediated feedforward loops in noise buffering. *PLoS computational biology*, 7(3):e1001101, 2011.

[19] Carla Bosia, Matteo Osella, Mariama El Baroudi, Davide Corà, and Michele Caselle. Gene autoregulation via intronic micrornas and its functions. *BMC systems biology*, 6:1–16, 2012.
